# Sulfenate anion catalyzed enantio- and diastereoselective aziridination

**DOI:** 10.1039/d5sc05077d

**Published:** 2025-09-01

**Authors:** Youge Pu, Anthony M. Smaldone, Javier Adrio, Patrick J. Walsh

**Affiliations:** a Roy and Diana Vagelos Laboratories, Department of Chemistry, University of Pennsylvania 231 South 34th Street Philadelphia PA USA pwalsh@sas.upenn.edu; b Department of Organic Chemistry, Institute for Advance Research in Chemical Sciences (IAdChem), Universidad Autónoma de Madrid Cantoblanco 28049-Madrid Spain javier.adrio@uam.es

## Abstract

The synthesis of enantioenriched aziridines is important for drug development due to their prevalence in bioactive molecules. Previous methods often use expensive catalysts, activated substrates, or show poor stereoselectivity. Herein, we report a novel organocatalytic approach using enantioenriched [2.2]paracyclophane (PCP)-based sulfenate anion catalysts, enabling the synthesis of 18 cyclopropanated aziridines from unactivated imines and commercially available benzyl chlorides in 50–99% yields with 73–99% ee and >20 : 1 dr. This approach fills a gap in the existing methods for aziridine synthesis, facilitating the generation of cyclopropyl-substituted aziridines with high stereoselectivity under mild and transition metal-free reaction conditions.

## Abstract 2

The first enantioselective sulfenate anion-catalyzed aziridination is developed. High enantio- and diastereoselectivities are obtained with cyclopropyl-substituted imines.

## Graphical abstract

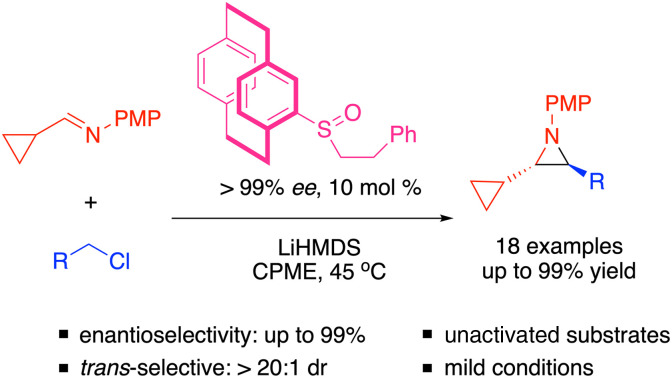

## Introduction

Enantioenriched aziridines serve as valuable scaffolds for various biologically active natural products, such as Mitomycins^1–3^ and Azinomycins.^4–6^ These structures are known for their antitumor, antibiotic, antimicrobial, neoplasm inhibiting, and glycosidase inhibitory properties.^7–9^ Due to the nature of their strained rings, aziridines serve as synthetic intermediates in ring-opening reactions^10,11^ to produce amine-derived products.^12^ They are also useful in ring-expansion reactions to form larger heterocycles,^13^ such as β-lactams,^14^ γ-lactams,^15^ pyrrolidines,^16^ and piperidines,^17^ which are essential in both organic synthesis and medicinal chemistry.

Classic methods for the stereoselective synthesis of aziridines^18–20^ often involve the addition of nitrenes to olefins,^21–23^ transfer of carbenes to imines,^24–27^ and the intramolecular cyclization of chiral 1,2-vicinal haloamines^28–30^ or amino alcohols.^31^ Many of these approaches use transition metal catalysts due to their efficiency and broad applicability. However, concerns regarding the high costs and sustainability of these catalysts have driven a shift toward more environmentally friendly methods. To address this, researchers have increasingly turned to organocatalysis^32,33^ as a promising alternative for aziridine synthesis.

**Scheme 1:**
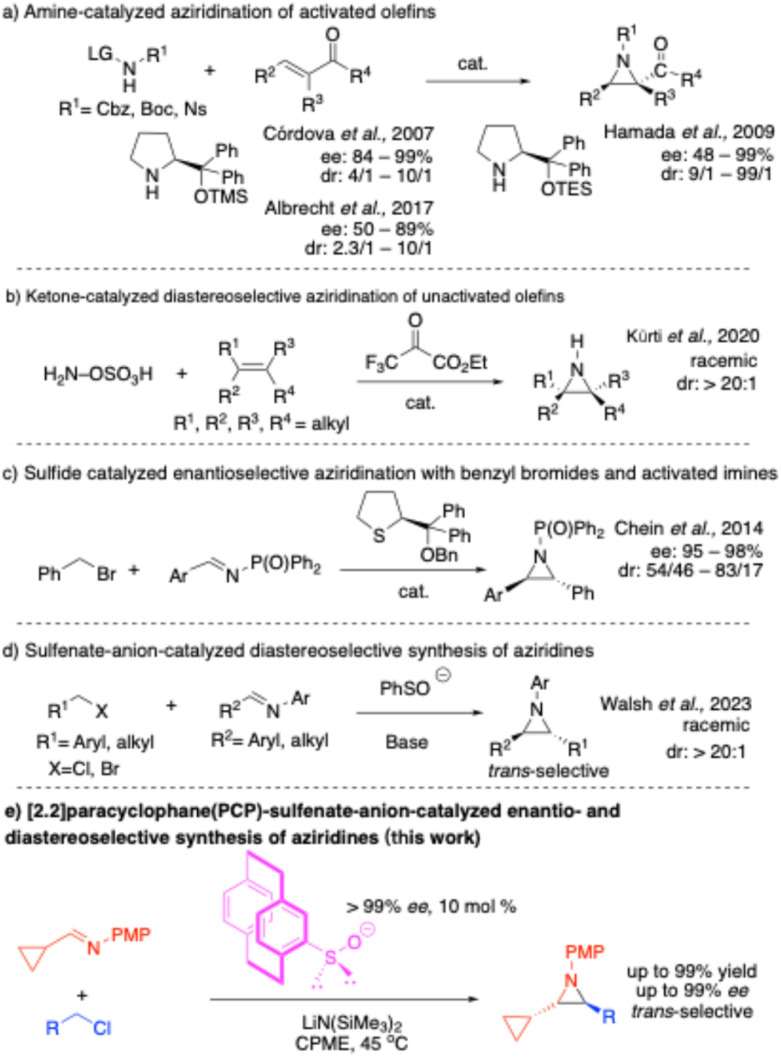
Recent advances in organocatalytic aziridination.

In this regard, the groups of Cordova,^34,35^ Hamada,^36^ and Albrecht^37^ have reported organocatalytic enantioselective aziridination reactions utilizing an aza-Michael-initiated ring-closing approach (Scheme 1a), which leads to good to excellent yields and stereoselectivities. However, these reactions, are typically limited to electronically activated substrates, such as α,β-unsaturated carbonyl compounds.

To expand the scope of this process, Kürti and coworkers developed a ketone catalyzed aziridination of unactivated olefins (Scheme 1b).^38^ This approach exploits an *in situ*-generated oxaziridine intermediate, enabling selective nitrogen transfer to unactivated carbon–carbon double bonds with excellent regio- and diastereoselectivity. This method has yet to be made enantioselective. Chein and coworkers^39^ developed an organocatalytic method for asymmetric aziridination with benzyl bromides and imines *via* the imino Corey–Chaykovsky reaction, using a tetrahydrothiophene-based chiral sulfide as the catalyst (Scheme 1c). This method achieved aziridination of *N*-phosphonate-activated benzaldimines with excellent enantioselectivities. Although these methods expand the range of accessible aziridines, the diastereoselectivities were moderate (dr = 54/46 to 83/17). Thus, complementary strategies for achieving high levels of enantio- and diastereoselectivity with unactivated substrates remain in demand.

Our group has been exploring sulfur-based organocatalysts and has successfully employed the sulfenate anion (RSO^−^), the conjugate base of sulfenic acids, in various catalytic reactions, including the synthesis of *trans*-stilbenes,^40,41^ stilbene-based polymers^42^ and a one-pot method to form all three bonds of diaryl alkynes.^43^ The nucleophilic nature of the sulfenate anion allows it to effectively attack electrophiles such as benzyl chlorides, while its ability to function as a leaving group facilitates the closure of the catalytic cycle. The sulfur changes oxidation state in the catalytic cycle^44–49^ and the intermediate sulfoxide activates the α-hydrogens toward deprotonation. Higher-valent sulfur species such as S(iv)^50^ and S(vi)^26,51–53^ have also been reported, illustrating the range of accessible oxidation states. Expanding on these advances, we have recently developed a diastereoselective method for the synthesis of racemic *trans*-aziridines from imines and benzylic or alkyl halides using sulfenate anion (PhSO^−^) catalysts^54^ (Scheme 1d). While this method affords good yields and high diastereoselectivities (*trans* : *cis* > 20 : 1), the catalyst is achiral, and the products are racemic.

To develop an enantioselective aziridination to couple the two electrophilic partners, we envisioned introducing an enantioenriched sulfenate anion catalyst. Herein, we report the first example of an asymmetric sulfenate anion catalyzed enantio- and diastereoselective aziridine formation (Scheme 1e) utilizing an enantioenriched [2.2]paracyclophane-substituted sulfoxide precatalyst. To our knowledge, this is the first example of asymmetric catalysis using an enantioenriched sulfenate anion.

## Results and discussion

### Proposed mechanism

**Fig. 1:**
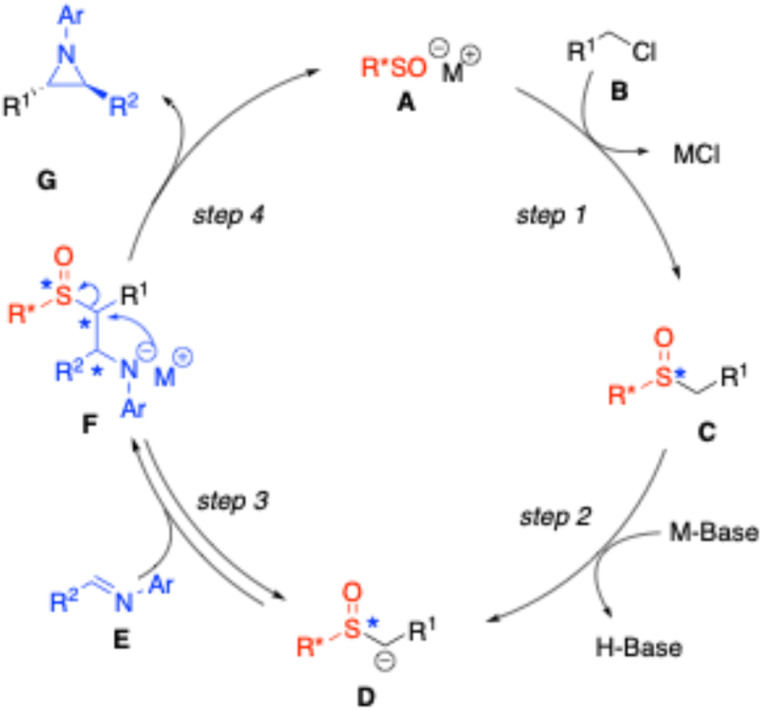
Proposed mechanism of enantio- and diastereoselective synthesis of aziridines.

Prior to discussing the specifics of catalyst design, we first outline the working mechanism (Fig. 1), as it informs the design process. The catalytic cycle begins with the generation of the sulfenate anion A.^54^ As a strong nucleophile, the sulfenate anion readily reacts with alkyl halides (B) to generate sulfoxide C with a change in oxidation state at sulfur. The resulting sulfoxide, in its more oxidized form, has activated α-hydrogens (p*K*_a_ ∼ 27.2 in DMSO)^55^ that can be deprotonated by moderately strong bases to yield the deprotonated intermediate D. Intermediate D is also a strong nucleophile but preferentially reacts with the imine E, leading to the formation of F. The basic nitrogen in F can then act as a nucleophile, displacing the sulfenate anion and closing the catalytic cycle with formation of the aziridine G.

The challenge in developing asymmetric sulfenate anion catalysts for aziridine synthesis is that there are two steps that form three new stereocenters in the catalytic reaction, as depicted in the proposed mechanism in Fig. 1. When the sulfenate anion R*SO^⊖^ (A) reacts with benzyl chloride, the sulfur lone pairs—previously enantiotopic in the achiral catalyst ArSO^⊖^—become diastereotopic in enantioenriched R*SO^⊖^. To generate a single diastereomer of the catalyst at the sulfoxide intermediate (C) the benzylation reaction must proceed with complete diastereoselectivity. We hypothesize that the configuration at sulfur in catalytic intermediate C will impact the formation of the two carbon-stereocenters in the addition adduct, D. Selecting an appropriate R* group presents a significant challenge, as the chirality of R*SO^⊖^ plays a role in the establishment of three contiguous stereocenters during the formation of intermediate F (one at sulfur and two at the carbons that will form the aziridine backbone).

### Reaction development

We envisioned catalysts R*SO^⊖^, where R* is a planar chiral *para*-cyclophane (PCP). Fortunately, beautiful work by the Perrio group on the *stoichiometric benzylation* of the *rac-para*-cyclophane, PCP–SO^⊖^, had been reported in 2008.^56^ This team demonstrated that under their conditions the planar chirality of the PCP group completely controlled the central chirality at sulfur during the S_N_2 reaction with benzyl bromide *via* the depicted conformation with the stereoselectivity shown (Table 1, entry 1–3). In this conformation (A0), one sulfur lone pair extends outward from the ring, while the other is buried between the two aromatic rings. This spatial arrangement directs nucleophilic attack to occur predominantly from the more exposed lone pair, ensuring high stereoselectivity. However, our conditions for the asymmetric aziridination are expected to differ. For example, we previously demonstrated that silyl amide bases were far more effective in racemic aziridine formation than the *tert-*butoxide base used by the Perrio group in the benzylation.^54^ Furthermore, the achiral sulfenate anion catalyst employed a Li^+^ counterion, rather than K^+^ and was performed in a different solvent. Given that main group counterions and solvents are well known to significantly impact reactivity and diastereoselectivity in organic reactions,^57,58^ these differences are likely to have significant implications in the benzylation at sulfur.

**Table 1:** Diastereoselective sulfenate salt alkylation

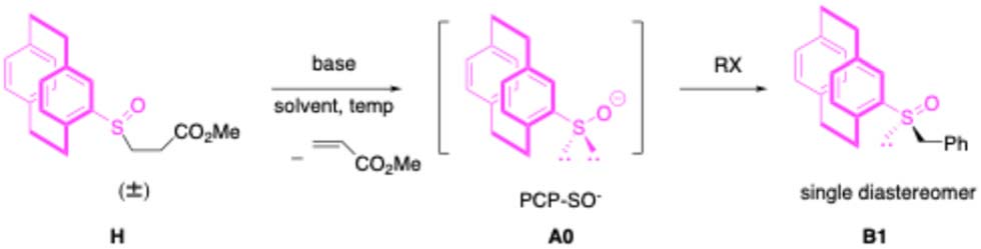					
Entry	RX	Base	Solvent	Temp	Yield^c^
1^a^	BnBr	^*t*^BuOK	THF	−78 °C	82%
2^a^	BnBr	^*t*^BuOK	THF	−40 °C	80%
3^a^	BnBr	^*t*^BuOK	THF	0 °C	77%
4^b^	BnCl	LiN(SiMe_3_)_2_	CPME	80 °C	78%

a Reactions performed by Perrio and coworkers.

b Reaction performed using 2 equiv. of BnCl, 2 equiv. of LiN(SiMe_3_)_2_ with CPME (0.1 M).

c Isolated yield.

With these considerations in mind, we chose conditions similar to our previous racemic aziridination studies,^54^ using LiN(SiMe_3_)_2_ to generate *rac-PCP*-SO^⊖^ at 80 °C in the presence of benzyl chloride. We were pleased to observe the formation of a single diastereomer of *PCP*–S(=O)CH_2_Ph (B1) in 78% yield (Table 1, entry 4). This result supports the formation of a single sulfoxide intermediate, maintaining the stereochemical integrity of the catalyst and preventing the generation of mixed diastereomers that could compromise the enantio- and diastereoselectivity in the aziridine forming steps.

**Scheme 2:**
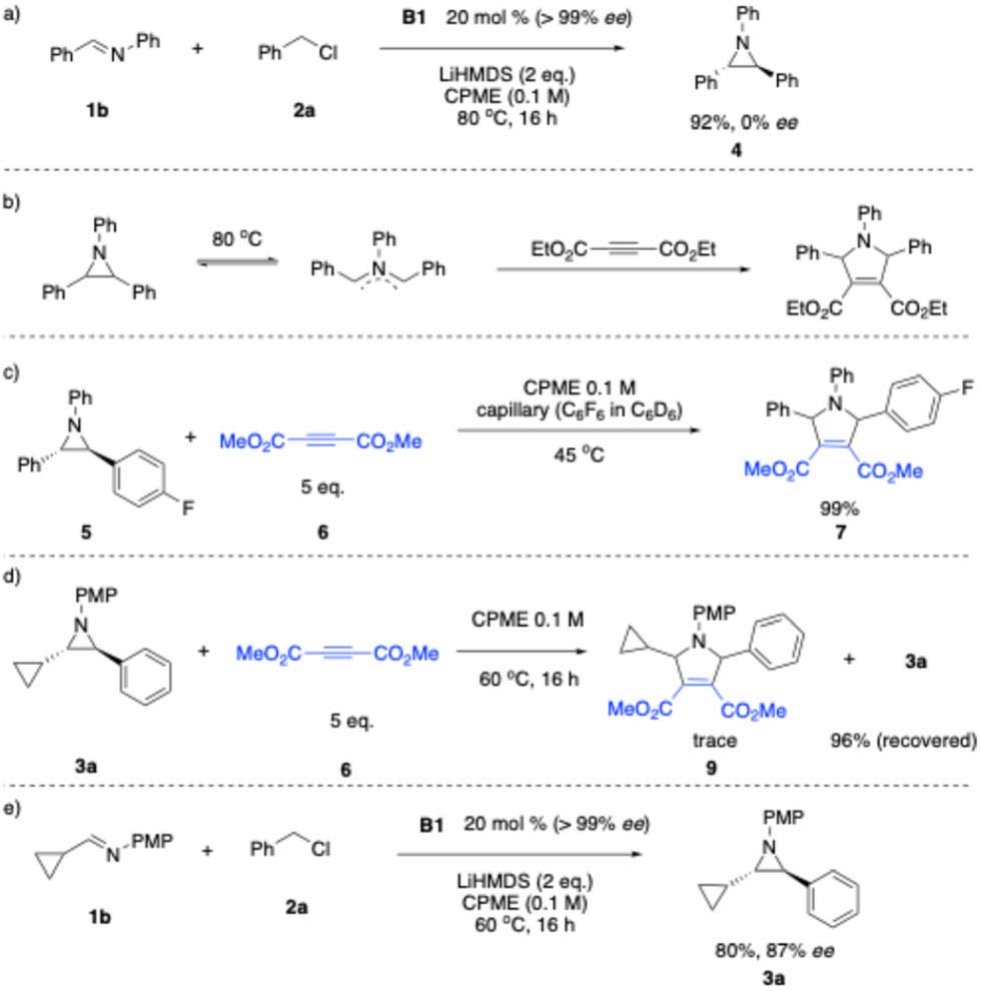
Enantioselective aziridination and studies on reversible aziridine ring-opening.

For proof-of-principle studies, we resolved racemic B1 into its enantiomers using preparative chiral phase HPLC on a small scale. We then employed the enantioenriched B1 (20 mol%) as catalyst in the presence of 1 equiv. (*E*)-*N*,1-Diphenylmethanimine (1b), benzyl chloride (2a) and 2 equiv. LiN(SiMe_3_)_2_ at 80 °C with the goal of preparing enantioenriched triphenyl aziridine (Scheme 2a). Despite achieving 92% yield of the desired product 4, it was found to be racemic.

The observation of 0% ee in asymmetric catalysis is quite informative.^59^ It usually indicates either that the enantioenriched “catalyst” is not actually catalyzing the reaction or there is a path for rapid racemization of the enantioenriched product. To investigate the origin of the observed 0% ee, we considered the possibility of aziridine racemization *via* thermal ring-opening. Aziridines are known to undergo thermally induced ring-opening to form ylide intermediates under certain conditions (Scheme 2b).^60,61^ To assess whether such a process contributes to racemization in our system, we examined the reactivity of fluorinated triphenyl aziridine 5 with dipolarophile 6 (5 equiv.) in CPME (0.1 M) at 45 °C in the presence of an internal standard (C_6_F_6_ in C_6_D_6_). The reaction was monitored by ^19^F{^1^H} NMR spectroscopy (Scheme 2c). After 20 h at 45 °C, full conversion of the aziridine to the corresponding cycloadduct was observed (99% assay yield, determined by ^19^F{^1^H} NMR). These results suggest that under the aziridine-forming reaction conditions in Scheme 2a, the aziridine product undergoes reversible ring-opening to form the higher energy achiral azomethine ylide, leading to racemization.

We hypothesized that an alkyl substituent on the imine would destabilize the azomethine ylide, thereby increasing the energy barrier for the ring opening/racemization process. To test this hypothesis, we examined the reactivity of cyclopropanated aziridine 3a with 6 at slightly lower temperature (CPME, 60 °C; Scheme 2d). Notably, only trace amounts of the corresponding 3-pyrroline product were observed, while 96% of the starting aziridine was recovered. This experiment indicates that alkyl-substituted azomethine ylides are significantly less prone to racemization through the azomethine ylide than their aryl-substituted counterparts.

Encouraged by these findings, we next examined the reaction of imine 1b and benzyl chloride using enantioenriched B1 as the catalyst (20 mol%, Scheme 2e). Notably, the desired product was obtained in 80% yield with 87% ee, demonstrating that the presence of an alkyl group is critical for suppressing the reversible ring-opening process and preserving enantioselectivity.

### Pre-catalyst design and synthesis

**Scheme 3:**
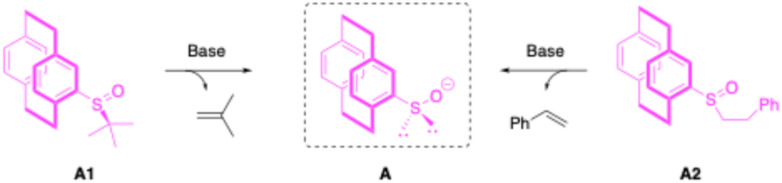
Pre-catalysts for the generation of sulfenate anions through base-promoted elimination.

Next, we prepared sulfoxide pre-catalysts that enable direct entry into the catalytic cycle *via* the sulfenate anion.^41^ Based on our prior experience, we hypothesized that both pre-catalysts A1 and A2 (Scheme 3) would be synthetically accessible and capable of generating the PCP-substituted sulfenate anion *via* base-promoted elimination of isobutene or styrene, respectively.

Both A1 (ref. 62 and 63) and A2 (ref. 64 and 65) were synthesized from *rac*-PCP-Br, followed by lithiation, and addition to enantioenriched sulfinyl reagents, yielding enantioenriched diastereomers that were separated by chromatography (see the SI for details). The ee of both catalysts were determined to be up to 99% by chiral stationary phase supercritical fluid chromatography (SFC).

### Reaction optimization

We then employed both pre-catalysts A1 and A2 in reaction optimization studies (Table 2). We began by optimizing the reaction conditions on a 0.1 mmol scale using model substrates: (*E*)-1-cyclopropyl-*N*-(4-methoxyphenyl)methanimine (1a) and benzyl chloride (2a), with a catalyst loading of 10 mol% (unless otherwise stated). A1 was selected for initial screening due to its more straightforward and efficient synthetic preparation. In a base screening with LiN(SiMe_3_)_2_, NaN(SiMe_3_)_2_, and KN(SiMe_3_)_2_ (entries 1–3), only LiN(SiMe_3_)_2_ showed high diastereoselectivity, yielding exclusively the *trans* product with an assay yield (AY) of 55% (determined by ^1^H NMR using dibromomethane as an internal standard, Table 2). NaN(SiMe_3_)_2_, and KN(SiMe_3_)_2_ both produced a significant amount of the *cis* product, and no enantioselectivity was observed.

**Table 2:** Optimization of the aziridination reaction with (*E*)-1-cyclopropyl-*N*-(4-methoxyphenyl)methanimine (1a) and benzyl chloride (2a)

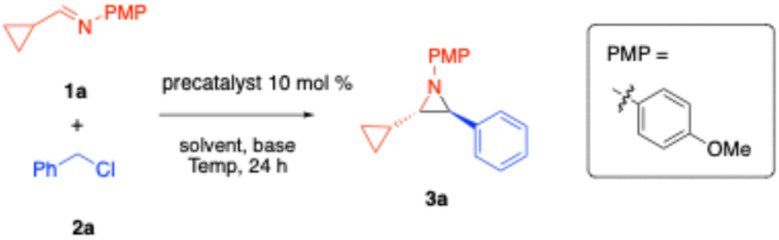					
Entry	Pre-cat.	Solvent	Temp.	Yield^a^^,^^b^	Ee^c^, dr^b^
1	A1	1,4-Dioxane	80 °C	55%	80%, >20 : 1
2^d^	A1	1,4-Dioxane	80 °C	41%	0%, 3 : 2
3^e^	A1	1,4-Dioxane	80 °C	76%	0%, 1 : 1
4^f^	A1	1,4-Dioxane	110 °C 30 min, then 80 °C	99%	76%, >20 : 1
5^f^	A1	1,4-Dioxane	110 °C 30 min, then 60 °C	99%	80%, >20 : 1
6^f^	A1	1,4-Dioxane	110 °C 30 min, then 45 °C	66%	83%, >20 : 1
7^f^	A1	CPME	110 °C 30 min, then 60 °C	28%	81%, >20 : 1
8^f^	A1	Toluene	110 °C 30 min, then 60 °C	Trace	N/A
9^f^	A1	^n^Bu_2_O	110 °C 30 min, then 60 °C	16%	N/A
10	A2	1,4-Dioxane	60 °C	70%	62%, >20 : 1
11	A2	CPME	60 °C	66%	88%, >20 : 1
12	A2	CPME	45 °C	70%	96%, >20 : 1
13	A2	CPME	25 °C	18%	94%, >20 : 1
14^g^	A2	CPME	45 °C	84%	97%, >20 : 1
15^h^	A2	CPME	45 °C	92%	97%, >20 : 1
16^i^	A2	CPME	45 °C	76%	94%, >20 : 1

a Reaction conditions: 1a (0.10 mmol, 1.0 equiv.), 2a (0.20 mmol, 2.0 equiv.), solvent (1.0 mL, 0.1 M), 24 h.

b Assay yield and dr's were determined by ^1^H NMR using CH_2_Br_2_ as the internal standard.

c ee was determined by SFC using column Chiralcel OJ3 with 5% MeOH and 95% CO_2_.

d NaN(SiMe_3_)_2_ was used as base.

e KN(SiMe_3_)_2_ was used as base.

f Pre-catalyst A1, base, and solvent were preheated at 110 °C for 30 min.

g 0.2 M in CPME.

h 0.4 M in CPME; 2 equiv. of 1a and 1 equiv. of 2a were used and the same yield and ee were observed.

i 0.4 M in CPME and 5 mol% A2 was used.

Recognizing that higher temperatures might be required to facilitate the elimination of isobutene from A1 and generate the sulfenate anion,^41^ we then started the reaction by pre-stirring the precatalyst, solvent, and base at 110 °C for 30 min. The reaction mixture was cooled to 80 °C, 60 °C, or 45 °C (entries 4–6), at which point the remaining reagents were added and the reaction was allowed to proceed for an additional 24 h. This pre-activation step significantly improved the yield from 55% (entry 1) to 99% (entries 4–5). Notably, entry 5 provided a higher ee of 80%. Further lowering the temperature did not significantly increase the ee but instead resulted in a substantial yield reduction (entry 6). Next, we evaluated a series of solvents, including CPME, toluene, and ^*n*^Bu_2_O (entries 7–9). While the reaction in CPME provided an enantioselectivity comparable to that achieved with dioxane, the yield decreased significantly. Toluene produced only trace amounts of the product (entry 8), and ^*n*^Bu_2_O resulted in a 16% yield (entry 9), both too low to obtain reliable ee and dr data. Integrating the results from base, temperature, and solvent screenings, the optimal conditions for precatalyst A1 were determined to be 10 mol% catalyst loading, 2 equiv. of LiN(SiMe_3_)_2_, and 1,4-dioxane (0.1 M), with pre-stirring at 110 °C for 30 min, followed by 60 °C for 24 h. Under these conditions, the final product 3a was obtained in 99% yield with 80% ee (entry 5). Attempts to further enhance the enantioselectivity, including the addition of various additives were unsuccessful (see the SI for details).

Given that precatalyst A2 was expected to generate the sulfenate anion more efficiently and under milder conditions, due to the greater acidity of its β-hydrogens and the stabilization of the styrene elimination product, it was subsequently used in the reaction optimization. Using the optimized conditions for pre-catalyst A1, we evaluated the two top-performing solvents with A2 as the pre-catalyst. While 1,4-dioxane achieved a slightly higher yield of 70% (entry 10), CPME provided a comparable yield of 66% (entry 11) but with a significantly improved enantiomeric excess (88% *vs.* 62% for 1,4-dioxane). Based on these results, CPME was selected as the solvent for further optimization. Considering that lower temperatures might enhance the enantioselectivity by favoring the pathway with the lowest activation energy, we decreased the temperature from 60 °C to 45 °C (entry 12) and then to 25 °C (entry 13). We were pleased to observe enantioselectivities of 96% and 94%, respectively. Since 45 °C maintained the yield (70%) while 25 °C resulted in only 18% yield, we selected 45 °C as the reaction temperature for further optimization. Increasing the solution concentration from 0.1 M to 0.2 M and 0.4 M resulted in a notable yield improvement (from 70% to 84% and 92%, entries 14 and 15) while holding the ee at 97%. Reducing the precatalyst loading from 10 mol% to 5 mol% led to a decrease in yield from 92% to 76% (entry 16). Adjusting the ratio of imine to benzyl chloride from 1 : 2 to 2 : 1 yielded identical results in terms of yield and ee (entry 15). However, the 2 : 1 ratio provided higher yields for other substrates, likely due to reduced byproduct formation from coupling of benzyl chlorides to form *trans*-stilbenes.^40^ As a result, the optimized reaction conditions for the aziridination employed 2 equiv. LiN(SiMe_3_)_2_ in CPME (0.4 M) with the pre-catalyst loading of 10 mol% at 60 °C and with a ratio of 2 : 1 imine to benzyl chloride (entry 15).

### Substrate scope

**Scheme 4:**
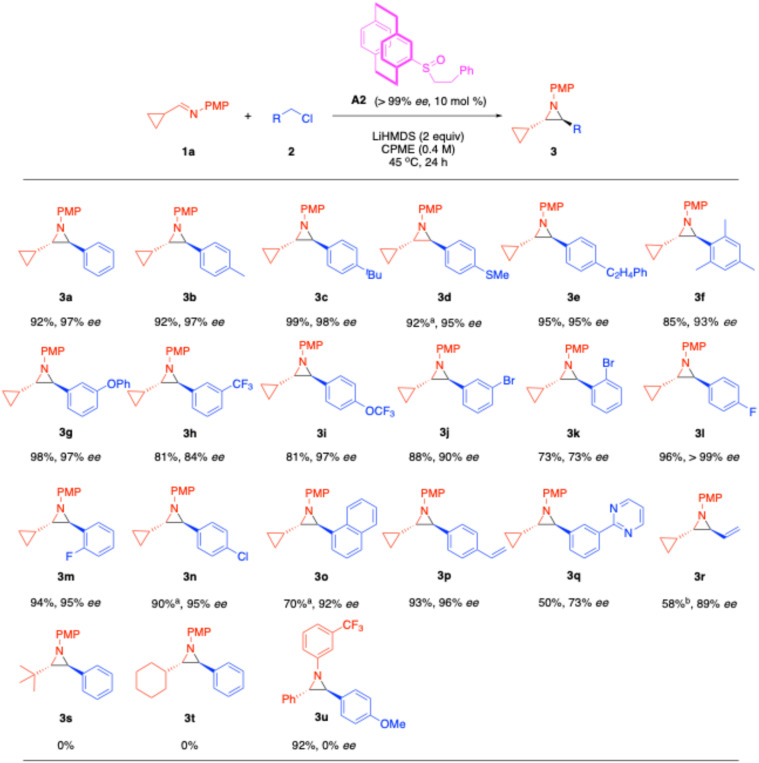
Substrate scope (dr's > 20 : 1). ^a^Reaction was conducted for 36 hours. ^b^Reaction was conducted with 4 equiv. of 2r.

With the optimized conditions in hand, we investigated the substrate scope with commercially available benzyl chlorides. As shown in Scheme 4, benzyl chlorides bearing electron-donating aryl groups such as 4-Me (2b), 4-^*t*^Bu (2c), 4-SMe (2d), 4-C_2_H_4_Ph (2e), and 1,3,5-Me_3_ (2f) provided *trans*-aziridines in 81–99% yield and 87–98% ee. Benzyl chlorides bearing electron-withdrawing substituents with 3-OPh (2g), 3-CF_3_ (2h) or 4-OCF_3_ (2l) cleanly afforded *trans*-aziridines 3g (98% yield, 97% ee), 3h (81% yield, 84% ee) and 3i (81% yield, 97% ee).

We were particularly interested in halogen-substituted aziridines due to their potential for further functionalization *via* transition metal-catalyzed cross-coupling reactions. Accordingly, using benzyl chlorides substituted with 3-Br (2j) and 2-Br (2k) groups led to the formation of *trans*-aziridine 3j in 88% yield with 90% ee and *trans*-aziridine 3k in 73% yield with 73% ee. The lower yield of 3k is attributed to the steric hindrance caused by the 2-bromo substituent. For benzyl chlorides bearing 4-F or 2-F, the enantioenriched *trans*-aziridine 3i and 3m were obtained in 96% with >99% ee and 94% yield with 95% ee, respectively. Similarly, 4-chlorobenzyl chloride was efficiently converted to *trans*-aziridine 3n in 90% yield and 95% ee.

We tested 1-(chloromethyl)naphthalene, with extended conjugation, which successfully yielded *trans*-aziridine 3o in 70% yield and 92% ee. Carbon–carbon double bonds are also of interest due to their potential for further elaboration, such as hydrofunctionalization,^66^ oxidation,^67^ or cross-coupling reactions.^68^ Accordingly, 4-vinylbenzyl chloride (2p) was used to deliver *trans*-aziridine 3p in 93% yield with 96% ee. Aziridines containing heterocycles are frequently used in drug molecules. As shown in Scheme 4, a pyrimidine-containing aziridine 3q was obtained in 50% yield with 73% ee.

We were interested to determine if electrophiles other than benzyl chlorides were suitable. Allyl chloride was attractive because the product would be a vinyl aziridine. In the event, the use of allyl chloride (2r) furnished the vinyl-substituted aziridine 3r in 58% yield with 89% ee, highlighting the potential of this method to make highly functionalized building blocks.^69–71^

Other alkyl aldehydes were evaluated for imine formation, including pivaldehyde and cyclohexanecarbaldehyde, to generate (*E*)-*N*-(4-methoxyphenyl)-2,2-dimethylpropan-1-imine (2s), (*E*)-1-cyclohexyl-*N*-(4-methoxyphenyl)methanimine (2t), and (*E*)-1-phenyl-*N*-(3-(trifluoromethyl)phenyl)methanimine (2u), separately. However, imine 2s did not yield any product likely due to increased steric hindrance. For imines with α-C–H's, like 2t, tautomerization to the enamine occurred at room temperature, resulting in no formation of the desired product 3t. Unfortunately, substrates that readily form enamines are not viable under our reaction conditions. In the case of 2u, we attempted to install an electron-withdrawing 3-C_6_H_4_-CF_3_ group on the imine nitrogen to reduce the stability of the possible ring-opening azomethine ylide intermediate (Scheme 2b). However, the resulting aziridine 3u was obtained in 92% yield with 0% ee, indicating that racemization is still occurring under our reaction conditions due to the ring-opening process.

### Gram-scale and X-ray structure determination

**Scheme 5:**
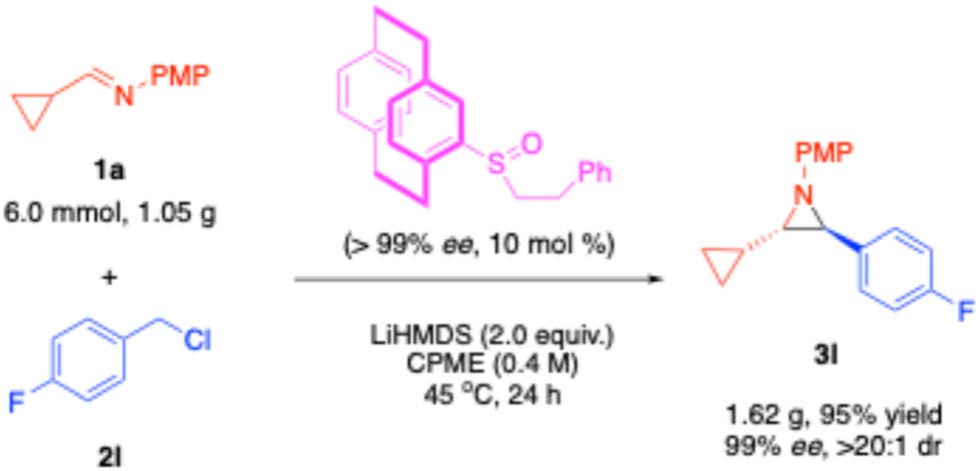
Gram-scale synthesis of aziridine 3l.

**Fig. 2:**
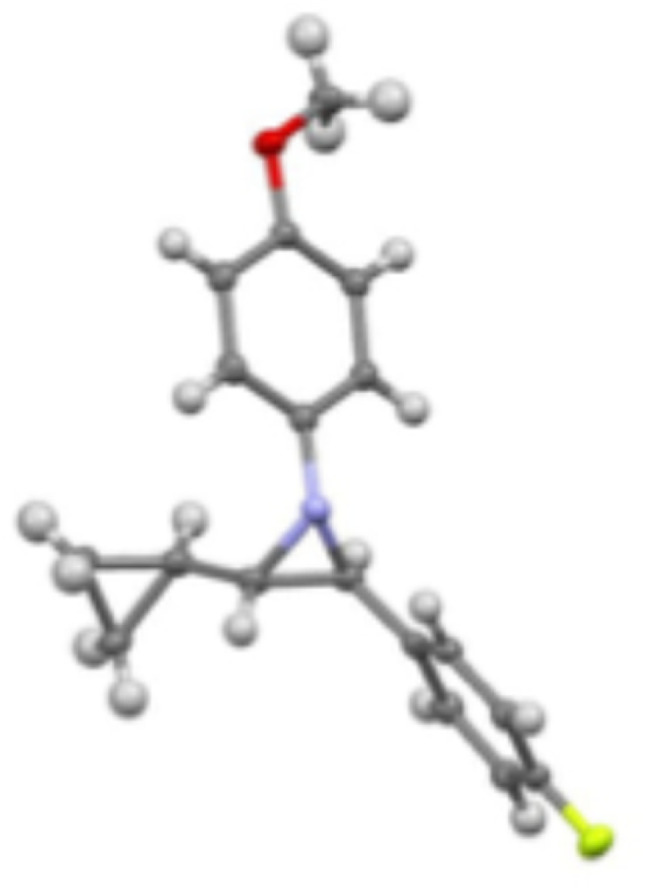
X-ray structure of (2*S*,3*S*) aziridine 3l (CCDC: 2422017).

To illustrate the scalability of this aziridine synthesis, compound 3l was prepared on a 6 mmol scale, yielding a 95% isolated yield (1.62 g) with 99% ee and >20 : 1 dr (Scheme 5). To determine the absolute configuration of the aziridines, a single crystal of the aziridine 3l was obtained through cooling 3l in a solution in hexanes from 40 °C to −16 °C. The X-ray crystal structure (Fig. 2, CCDC: 2422017) confirmed that the aziridine was the (2*S*,3*S*) isomer (see the SI for crystallographic data), which was obtained by using the precatalyst A2 with a R_*p*_ configuration.

## Conclusions

In conclusion, we have developed the first enantioselective sulfenate anion-catalyzed method, which has been showcased in the highly enantioselective synthesis of *trans*-aziridines from simple benzyl chlorides and imines. The reaction proceeds with good to excellent yields and enantioselectivities. The [2.2]paracyclophane (PCP) scaffold is instrumental in providing excellent stereocontrol, not only in the formation of the sulfur stereocenter, but also in the generation of the two stereocenters in the aziridine backbone. Although the current scope is somewhat restricted due to ring opening of certain aziridines to achiral azomethane ylides, it provides proof-of-concept that this strategy is viable and enables the synthesis of enantioenriched cyclopropyl aziridines, a class of compounds not previously accessible with high levels of enantioselectivity. Further studies to develop more efficient oxidation state altering main group catalysts are under way in our laboratories.

## Author contributions

P. J. W. conceived the project and supervised the research. J. A. conducted preliminary experiments and Y. P. conducted the experimental work and data analysis with A. S. P. J. W and Y. P. wrote the original draft of the manuscript. All authors contributed to the discussion and revision of the manuscript.

## Conflicts of interest

The authors declare no competing financial interest.

## Supplementary Material

SC-016-D5SC05077D-s001

## Data Availability

CCDC 2422017 contains the supplementary crystallographic data for this paper.^72^ All data for this manuscript is included in the SI. Supporting information: The experimental procedures, characterization data and crystallographic data. Deposition Number 2422017 contains the supplementary crystallographic data for this paper. See DOI: https://doi.org/10.1039/d5sc05077d.
