# Supplementary material for: Sulfenate anion catalyzed enantio- and diastereoselective aziridination
Source: Chem Sci. 2025 Sep 1;16(39):18167–75. doi: 10.1039/d5sc05077d (PMC12419035; doi:10.1039/d5sc05077d)
Supplement: SC-016-D5SC05077D-s001 [file SC-016-D5SC05077D-s001.pdf]

Supplementary Information (SI) for Chemical Science.
This journal is © The Royal Society of Chemistry 2025

Supporting Information

Sulfenate Anion-Catalyzed Enantio- and Diastereoselective Synthesis of Aziridines

Youge Pu,[1] Anthony M. Smaldone,[1] Javier Adrio,[1,2,]* and Patrick Walsh[1,]*
[1]Roy and Diana Vagelos Laboratories, Department of Chemistry, University of Pennsylvania, Philadelphia, PA, USA
[2]Department of Organic Chemistry, Institute for Advance Research in Chemical Sciences (IAdChem), Universidad Autónoma de Madrid, Cantoblanco, 28049-Madrid, Spain

Table of contents

1. General methods, synthesis of imines, and the sulfoxide precatalyst..S3
2. Procedure and characterization for the aziridines catalyzed by sulfenate anion................................S7
3. Procedure for gram scale synthesis of aziridines **3l**..S13
4. Studies on reversible aziridine ring-opening/trapping.. S14
5. Crystallographic data of aziridine **3l**………………………………...S15
6. References...S16
7. SFC analysis and spectra..S17
8. NMR spectra...S32

General Methods: All aziridination reactions were carried out under dry nitrogen. Anhydrous solvents including cyclopentymethyl ether (CPME), 1,4-dioxane, dibutyl ether, toluene was purchased from Acros and directly used without further purification. Lithium bis(trimethylsilyl)amide was purchased from Thermo Scientific Chemicals and was recrystallized in hexanes. Unless otherwise stated, reagents were commercially available and used as purchased. Chemicals were purchased from Sigma-Aldrich, Acros, Alfa Aesar or Matrix Scientific. TLC was performed with Merck TLC Silicagel60 F254 plates and detection was under UV light at 220 nm. Flash chromatography was performed with silica gel (230–400 mesh, Silicycle). The ^{1}H and ^{13}C{^{1}H} and^{19}F{^{1}H} NMR spectra were obtained using a Bruker Model Advance DMX 400 Spectrometer (^{1}H 400 MHz, ^{19}F{^{1}H} 375 MHz, and ^{13}C{^{1}H} 101 MHz, respectively). Chemical shifts are reported in units of parts per million (ppm) downfield from tetramethylsilane (TMS), and all coupling constants are reported in hertz (Hz). High resolution mass spectrometry (HRMS) data were obtained on a Waters LC-TOF mass spectrometer (model LCT-XE Premier) using chemical ionization (CI) or electrospray ionization (ESI) in positive or negative mode, depending on the analyte. Enantiomeric excess (*ee*) of the compounds were obtained on Supercritical Fluid Chromatography (SFC) using Daicel Chiralcel® columns (250 x 4.6 mm) OJ-3, IA-3, IG-3 or IF-3 column, with a flow of 2.5 mL min–1.

Preparation of imines: Imine **2b** was commercially available from Alfa Aesar and imine **2a** were prepared according to a modified literature procedure.[1] To an oven-dried 20 mL vial charged with a stir bar were added cyclopropanecarboxaldehyde (10 mmol, 1.0 equiv., 746 uL), *p*-anisidine amine (10 mmol, 1.0 equiv., 1.23 g), and 4 Å molecular sieves (2.0 g) in anhydrous THF (10 mL) in a dry box under a nitrogen atmosphere. The vial was capped with a screw cap, and the reaction mixture was moved out of the glovebox and then stirred at 50 ºC in a reaction block for 16 h. The reaction mixture was then filtrated over Celite packed in a glass frit funnel, after which the solvent of the filtrate was removed under reduced pressure. The residue was recrystallized using hexanes to give the product as a white solid (1.629 g, 93%). The spectroscopic data match the previously reported data.[1]

Preparation of the sulfoxide precatalyst A1: Sulfoxide precatalyst **A1** was prepared in the following 3 steps according to the literature procedures with slightly modification.[2]

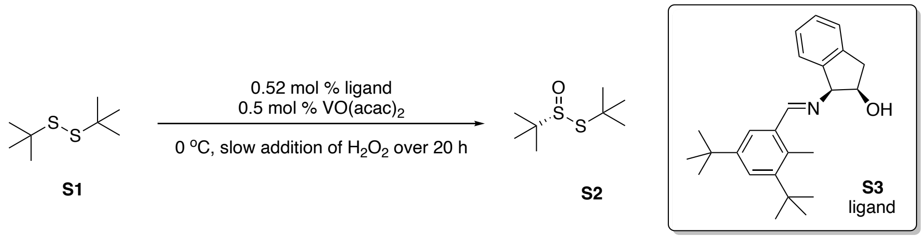

***R*-(*tert*-butyl) *tert*-butanethiosulfinate** (**S2**): Following the procedure,[3] to a 250 mL two neck flask was charged a stir bar, vanadyl acetylacetone (292 mg, 1.1 mmol) and ligand **S3** (414 mg, 1.14 mmol) in acetone (60 mL) were stirred vigorously in air for 30 min. To the resulting dark green solution was added di-*tert*-butyl disulfide **S1** (43 mL, 0.22 mol) and the resulting mixture was cooled to 0 ºC in an ice bath. The reaction mixture was stirred vigorously, and 30% aq. hydrogen peroxide was added over 20 h using a syringe pump. During the addition, the color of reaction mixture changed from dark green to dark brown. The resulting mixture was quenched at 0 ºC with saturated aqueous $Na_2S_2O_3$ (12 mL) added over 30 min via syringe pump. The mixture was diluted with hexanes (55 mL) and transferred to a separatory funnel. The layers were separated, and the aqueous layer was washed with hexanes (2 x 55 mL) and the combined organic layers were washed with brine (2 x 12 mL). The combined organic layers were then dried over Na_2SO_4, filtered, and the solvent was removed under reduced pressure to yield an orange-colored oil. The crude product was purified by flash chromatography on silica gel (eluted with hexanes: ethyl acetate = 4:1) to give the product **S2** as a yellow solid (2.90 g, 70%, 86% *ee*). The spectroscopic data match the previously

reported data.[3] Enantioselectivity was determined by SFC analysis (Chiralcel-IG-3, 2.5 mL/min, 254 nm, CO_2/MeOH, 99/1); t_{R1} = 3.6 min, t_{R2} = 5.0 min.

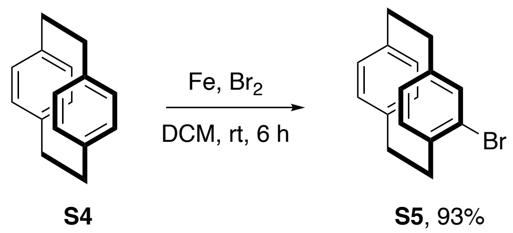

(±)-4-Bromo[2.2]paracyclophane (S5): To a 20 mL vial charged with a stir bar was added bromine (263 uL, 5.1 mmol, 1.02 equiv.) and DCM (5.6 mL) by syringe in air. 347 uL of the bromine solution was transferred to a separate 4 mL vial containing iron filings (5.6 mg, 0.1 mmol, 2 mol %) and a stir bar. The mixture was stirred at rt for 1 h. The reaction mixture was then transferred to a 100 mL round-bottom flask, diluted with DCM (17.4 mL), and [2.2]paracyclophane (1.04 g, 5 mmol, 1.0 equiv.) was added at room temperature. The mixture was stirred for an additional 20 min before the remaining bromine solution in DCM was added via syringe pump over 5 h. The resulting dark brown mixture was quenched at rt with saturated aqueous $Na_2S_2O_3$ (20 mL). The aqueous phase was extracted with DCM (3 x 15 mL) and the combined organic layers were washed with brine (15 mL). The combined organic layers were then dried over Na_2SO_4, filtered, and the solvent was removed under reduced pressure. The crude product was purified by flash chromatography on silica gel (eluted with hexanes : ethyl acetate = 99:1 – 90:1) to give the product as a white solid (1.33 g, 93%). The spectroscopic data match the previously reported data.[2]

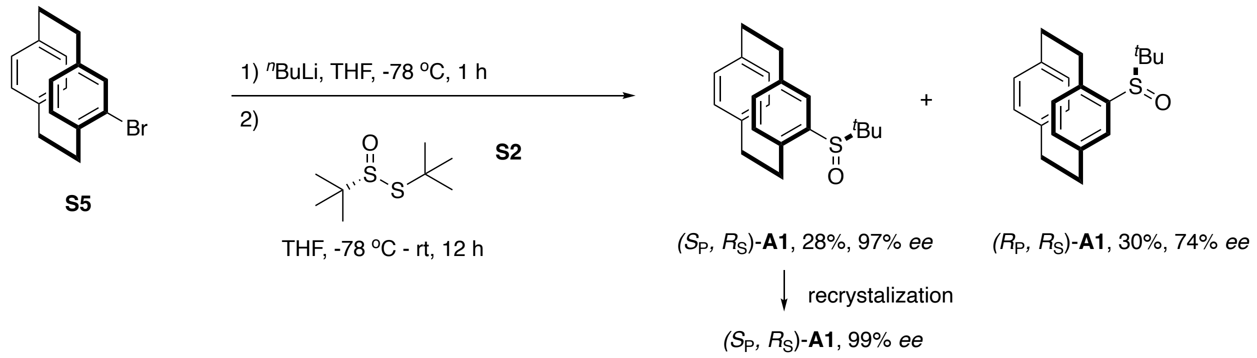

(S_P,R_S)-(–)-4-*tert*-Butylsulfinyl[2.2]paracyclophane [(S_P,R_S)-A1] and (R_P,R_S)-(–)-4-*tert*-Butylsulfinyl[2.2]paracyclophane [(R_P,R_S)-A1]: A 250 mL Schlenk flask charged with a stir bar was connected to a Schlenk line under a nitrogen atmosphere. (±)-4-Bromo[2.2]paracyclophane (**S5**) (1.33 g, 4.63 mmol, 1.0 equiv.) was added and dissolved in THF (50 mL) at room temperature. The mixture was then cooled to -78 °C in an acetone/dry ice bath, and *n*-BuLi (1.6 M in hexanes, 3.18 mL, 5.09 mmol, 1.1 equiv.) was added dropwise over 15 min. The color of the reaction mixture changed from colorless to orange. After 45 min, a 25 mL round-bottom flask containing R-(*tert*-butyl) *tert*-butanethiosulfinate **S2** (1.35 g, 6.95 mmol, 1.5 equiv.) was degassed under vacuum and backfilled with nitrogen on the Schlenk line. 10 mL dry THF was then added, resulting in a light-yellow solution, which was subsequently added to the prior solution at -78 °C. The reaction mixture was stirred at -78 °C for an additional hour, then gradually warmed to room temperature, and stirred for additional 12 h. The of color of reaction mixture then changed from orange to light yellow. The reaction mixture was then opened to air and the solvent was removed under reduced pressure. The crude product was purified by flash chromatography on silica gel (eluted with hexanes : ethyl acetate = 95:5 – 80:20) to give both (S_P,R_S)-**A1** (405 mg, 28%, 97% *ee*) and (R_P,R_S)-**A1**(434 mg, 30%, 74%

ee) as white solids. The spectroscopic data match the previously reported data.[2] (S_P, R_S)-**A1** was recrystallized from $CHCl_3$/hexanes (1:9) from rt to -16 °C in a freezer over 16 h, affording a colorless crystal (330 mg, 81%, 99% *ee*). Enantioselectivity was determined by SFC analysis (Chiralcel-OJ-3, 2.5 mL/min, 254 nm, CO_2/MeOH, 97.5/2.5); t_{R1} = 2.3 min, t_{R2} = 7.2 min.

Preparation of the sulfoxide precatalyst A2: Sulfoxide precatalyst **A2** was prepared in the following 4 steps according to the literature procedures with slightly modification.[4]

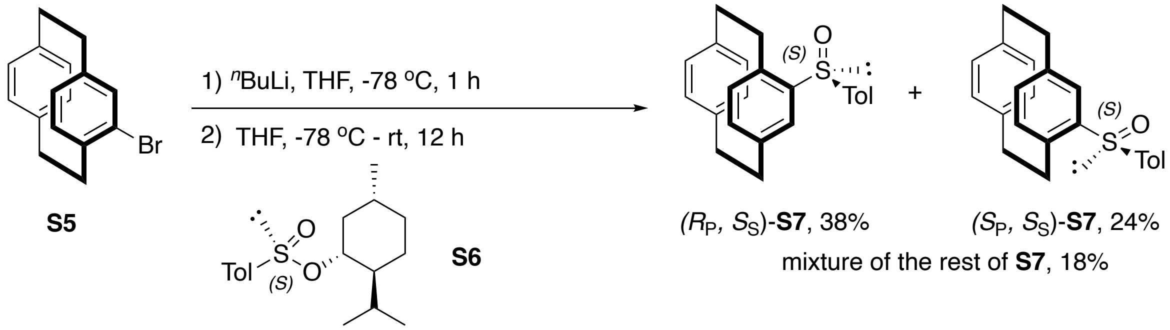

(R_p,S_S)-4-*p*-Toluenesulfinyl[2.2]paracyclophane, [(R_p,S_S)-S7] and (S_p,S_S)-4-*p*-toluenesulfinyl[2.2]paracyclophane [(S_p,S_S)-S7]: A 250 mL Schlenk flask charged with a stir bar was connected to a Schlenk line under a nitrogen atmosphere. (±)-4-Bromo[2.2]paracyclophane (**S5**) (1.436 g, 5.0 mmol, 1.0 equiv.) was added and dissolved in THF (25 mL). The mixture was then cooled to -78 °C in an acetone/dry ice bath and *n*-BuLi (1.6 M in hexanes, 3.28 mL, 5.25 mmol, 1.05 equiv.) was added dropwise over 15 min. The resulting yellow solution was stirred for 2 h before being added in one portion to a solution of (1*R*, 2*S*, 5*R*)-(−)-menthyl(*S*)-*p*-toluenesulfinate **S6** (1.545 g, 5.25 mmol, 1.05 equiv.) in THF (26 mL) at −78 °C. The resulting orange solution was warmed to room temperature and stirred for 12 h. The color of the reaction mixture then changed from orange to light yellow. The reaction mixture was exposed to air, quenched with saturated aqueous ammonium chloride solution (20 mL) and extracted with ethyl acetate (3 x 20 mL). The combined organic layers were then dried over $MgSO_4$, filtered, and the solvent was removed under reduced pressure. The crude product was purified by flash chromatography on silica gel (eluted with hexanes : ethyl acetate = 4:1) to afford (R_P, S_S)-**S7** (650 mg, 38%), (S_P, S_S)-**S7** (413 mg, 24%), and a non-separable fraction of the product **S7** (308 mg, 18%) as white solids, achieving a total yield of 80%. The spectroscopic data match the previously reported data.[4]

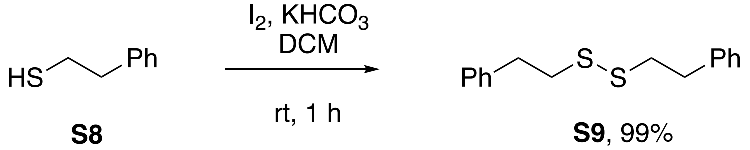

1,2-diphenethyldisulfane (S9): Following a modified procedure,[5] to a 250 mL round-bottom flask with a stir bar was add 2-phenylethanethiol **S8** (1.34 mL, 10 mmol, 1 equiv.), potassium bicarbonate (2 g, 20 mmol, 2 equiv.), and 10 mL DCM in air at room temperature. A solution of iodine (951.8 mg, 7.5 mmol, 0.75 mmol) in 40 mL DCM was added slowly over 3 min to the flask. The flask was open to air to release the pressure built up during this process. The color of the reaction mixture changed from clear to dark brown and the mixture was stirred for 1 h at room temperature. A saturated sodium sulfite solution (30 mL) was then added to the reaction mixture at room temperature to decompose the excess iodine and the mixture was then stirred at room temperature for 15 min until the color turned to transparent. The crude product was transferred to a separatory funnel and extracted with DCM (3 x 30 mL), then the organic phase was combined and washed with brine (50 mL). The combined organic layers were dried over $MgSO_4$, filtered and the volatile materials were removed using a rotary evaporator. The crude product (2.71 g, 99%) was

dried under reduced pressure on the Schlenk line to afford a yellow oil that was used for the next step without further purification. ^{1}H NMR (400 MHz, $CDCl_3$) δ 7.38 – 7.30 (m, 4H), 7.30 – 7.21 (m, 6H), 3.07 – 3.00 (m, 4H), 3.00 – 2.95 (m, 4H). $^{13}C\{^1H\}$ NMR (101 MHz, $CDCl_3$) δ 140.07, 128.66, 128.56, 126.45, 40.27, 35.78. HRMS calculated for $C_{16}H_{19}S_2$: 275.0928, observed: 275.0922 $[M+H]^+$.

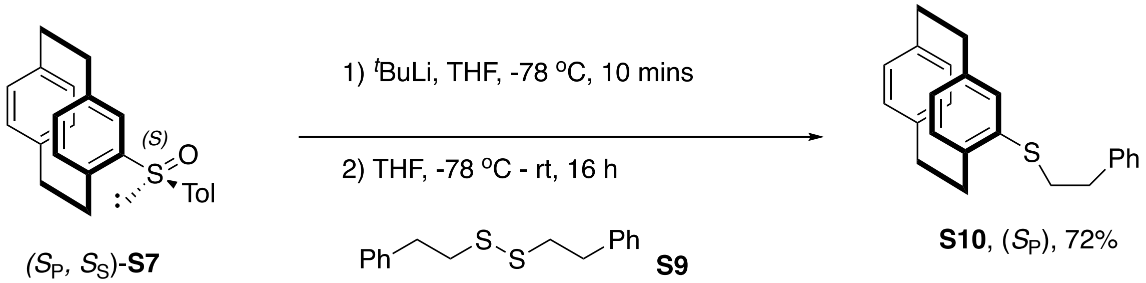

(S_P, S_S)-**S7** **S9** **S10**, (S_P), 72%

(S_P)-4-*p*-phenylethylsulfanyl[2.2]paracyclophane [(S_P)-S10]: To a 100 mL Schlenk flask under nitrogen atmosphere, a solution of *tert*-butyllithium (1.7 M in hexane; 4.53 mL, 7.7 mmol, 4.0 equiv.) was carefully added dropwise to a solution of (S_P, S_S)-**S7** (667.3 mg, 1.93 mmol, 1.0 eq.) in THF (20 mL) at -78 °C in an acetone/dry ice bath. The resulting orange solution was stirred for 10 min and then a solution of **S9** (4.23 g, 15.4 mmol, 8.0 equiv.) in THF (5.2 mL) was added by syringe in one portion. The reaction mixture was stirred at -78 °C for another 15 minutes, warmed to room temperature and stirred for another 16 h. The reaction flask was opened to air, poured into brine (25 mL) and extracted with ethyl acetate (3 x 25 mL). The combined organic layers were dried over $MgSO_4$, filtered, and the volatile materials were removed using a rotary evaporator. The crude product was purified by flash chromatography on silica gel (eluted with hexanes : dichloromethane = 5:1) to afford **S10** (482 mg, 72% IY) as a white solid. ^{1}H NMR (400 MHz, $CDCl_3$) δ 7.42 – 7.34 (m, 2H), 7.34 – 7.22 (m, 3H), 7.02 (dd, J = 7.9, 2.0 Hz, 1H), 6.61 (dd, J = 7.8, 2.0 Hz, 1H), 6.57 – 6.44 (m, 3H), 6.47 – 6.38 (m, 2H), 3.58 (ddd, J = 13.3, 10.1, 2.2 Hz, 1H), 3.28 (ddd, J = 13.0, 10.1, 5.9 Hz, 1H), 3.20 – 2.95 (m, 7H), 2.97 – 2.80 (m, 3H). $^{13}C\{^1H\}$ NMR (101 MHz, $CDCl_3$) δ 140.54, 140.37, 139.91, 139.52, 139.19, 137.32, 134.76, 134.39, 133.37, 132.87, 131.93, 130.78, 128.84, 128.58, 128.55, 126.46, 35.87, 35.47, 35.20, 35.13, 34.43, 33.82. HRMS calculated for $C_{24}H_{25}S$: 345.1677, observed: 345.1668 $[M+H]^+$.

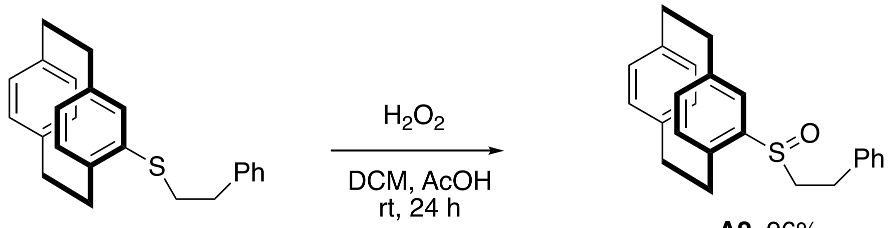

S10, (S_P) **A2**, 96%
(S_P, S_S) and (S_P, R_S) (dr = 1:1)

(S_P, S_S)-4-*p*-phenylethylsulfinyl[2.2]paracyclophane [(S_p,S_S)-A2] and (S_P, R_S)-4-*p*-phenylethylsulfinyl[2.2]paracyclophane [(S_p,R_S)-A2]: To a 20 mL vial charged with a stir bar, sulfide ***S_P*)-S10** (482 mg, 1.4 mmol, 1.0 equiv.), 2 mL of acetic acid, and 1 mL of DCM was added, followed by the dropwise addition of an H_2O_2 solution (30 % (w/w) in H_2O, 157 μL, 1.54 mmol, 1.1 equiv.) at 0 °C in an ice bath. The reaction mixture was then warmed to room temperature and stirred for 24 h. Water (3 mL) was added, and the mixture was extracted with DCM (3 x 3 mL). The combined organic layers were dried over $MgSO_4$, filtered and the volatile materials were removed using a rotary evaporator. The crude product was purified by flash chromatography on silica gel (eluted with hexanes : ethyl acetate = 4:1) to afford **A2** (484 mg, 96% yield, 99% *ee*, dr = 1:1) as a yellow oil. **A2** was used directly for aziridinations without further separation of the diastereomers. First diastereomer **A2**-1: ^{1}H NMR (500 MHz, $CDCl_3$) δ 7.34 – 7.26 (m, 2H), 7.26 – 7.20 (m, 1H), 7.20 – 7.12 (m, 2H), 6.89 (dd, J = 8.0, 1.8 Hz, 1H), 6.69 – 6.52 (m, 5H), 6.48 (dd, J =

7.9, 1.8 Hz, 1H), 4.25 (ddd, J = 12.8, 10.5, 2.4 Hz, 1H), 3.38 (ddd, J = 12.7, 10.5, 5.2 Hz, 1H), 3.33 – 2.89 (m, 10H). $^{13}C\{^1H\}$ NMR (126 MHz, $CDCl_3$) δ 142.97, 141.90, 139.65, 139.09, 138.85, 135.84, 135.76, 135.25, 133.10, 133.05, 133.00, 131.18, 128.68, 128.53, 128.45, 126.59, 56.75, 35.27, 35.19, 34.50, 32.61, 28.13. Second diastereomer **A2**-2: ^1H NMR (500 MHz, $CDCl_3$) δ 7.29 (t, J = 7.4 Hz, 2H), 7.26 – 7.17 (m, 1H), 7.20 – 7.13 (m, 2H), 7.05 (d, J = 1.9 Hz, 1H), 6.87 (dd, J = 7.8, 1.9 Hz, 1H), 6.67 (dd, J = 7.8, 1.9 Hz, 1H), 6.59 (dd, J = 7.8, 1.9 Hz, 1H), 6.58 – 6.47 (m, 3H), 3.25 – 2.96 (m, 9H), 2.92 – 2.70 (m, 3H). $^{13}C\{^1H\}$ NMR (126 MHz, $CDCl_3$) δ 142.97, 141.90, 139.65, 139.09, 138.85, 135.84, 135.76, 135.25, 133.10, 133.05, 133.00, 131.18, 128.68, 128.53, 128.45, 126.59, 56.75, 35.27, 35.19, 34.50, 32.61, 28.13. HRMS calculated for $C_{24}H_{25}OS$: 361.1626, observed: 361.1620 $[M+H]^+$. Enantioselectivity was determined by SFC analysis (Chiralcel-OJ-3, 2.5 mL/min, 254 nm, CO_2/MeOH, 95/5); t_{R1} = 4.1 min, t_{R2} = 8.3 min.

General Procedure A for the synthesis of aziridines from benzyl chlorides: An oven-dried 4 mL reaction vial equipped with a stir bar was brought into the nitrogen filled glovebox. To the vial was added (E)-1-cyclopropyl-N-(4-methoxyphenyl)methanimine (**1a**, 0.2 mmol, 2.0 equiv.), pre-catalyst (**A2**, 0.01 mmol, 10 mol %), $LiN(SiMe_3)_2$ (33.5 mg, 0.20 mmol, 2.0 equiv.), and CPME (0.25 mL). Next, the benzyl chloride (**1**, 0.10 mmol, 1.0 equiv.) was added through a single channel pipette. The reaction vial was capped with a screw cap with a septum and transferred out of the glove box. The reaction mixture was heated at 45 °C and stirred for 24 h. The resulting cloudy yellow reaction mixture was cooled to room temperature, opened to air and quenched with 3 drops of water. The reaction mixture was then passed through a short pad of Celite packed into a syringe. The pad was then rinsed with 10 mL ethyl acetate and the solvent was removed under reduced pressure. The crude product was purified by flash chromatography on silica gel (deactivated with triethylamine by flashing 3% triethylamine in hexanes through the column, loading the sample and eluted with hexanes and ethyl acetate) to give the product.

(2*S*,3*S*)-2-cyclopropyl-1-(4-methoxyphenyl)-3-phenylaziridine (3a)

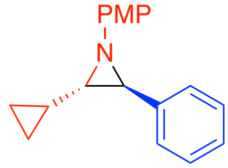

The reaction was performed following General Procedure A with (E)-1-cyclopropyl-N-(4-methoxyphenyl)methanimine (**1a**) and benzyl chloride (**2a**). The crude product was purified by flash chromatography on deactivated silica gel (eluted with hexanes: ethyl acetate = 98:2) to give the product as a pale-yellow oil (24.5 mg, 92% yield, 97% ee). The spectroscopic data match the previously reported data.[1] ^1H NMR (400 MHz, CDCl3) δ 7.38 – 7.23 (m, 5H), 7.01 – 6.93 (m, 2H), 6.86 – 6.77 (m, 2H), 3.79 (s, 3H), 3.14 (d, J = 3.1 Hz, 1H), 2.15 (dd, J = 7.0, 3.1 Hz, 1H), 0.75 – 0.63 (m, 1H), 0.62 – 0.47 (m, 3H), 0.42 (m, 1H). $^{13}C\{^1H\}$ NMR (101 MHz, $CDCl_3$) δ 154.83, 143.43, 138.60, 128.29, 127.12, 126.63, 121.78, 114.07, 55.51, 52.33, 46.75, 10.10, 3.99, 3.04. Enantioselectivity was determined by SFC analysis (Chiralcel-OJ-3, 2.5 mL/min, 254 nm, CO_2/MeOH, 95/5); t_{R1} = 2.4 min, t_{R2} = 3.1 min.

(2*S*,3*S*)-2-cyclopropyl-1-(4-methoxyphenyl)-3-(*p*-tolyl)aziridine (3b)

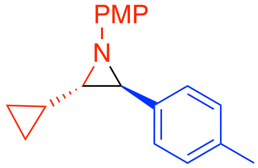

The reaction was performed following General Procedure A with (E)-1-cyclopropyl-N-(4-methoxyphenyl)methanimine (**1a**) and 1-(chloromethyl)-4-methylbenzene (**2b**). The crude product was purified by flash chromatography on deactivated silica gel (eluted with hexanes: ethyl acetate = 98:2) to give the product as a white solid (25.7 mg, 92% yield, 97% ee). ^1H NMR (400 MHz, $CDCl_3$) δ 7.15 (m, 4H), 6.99 – 6.90 (m, 2H), 6.85 – 6.71 (m, 2H), 3.78 (s, 3H), 3.10 (d, J = 3.1 Hz, 1H), 2.35 (s, 3H), 2.13 (dd, J =

6.2 Hz, J = 3.1 Hz, 1H), 0.67 (dt, J = 6.0, 2.9 Hz, 1H), 0.61 – 0.46 (m, 3H), 0.49 – 0.36 (m, 1H). $^{13}C\{^1H\}$ NMR (101 MHz, $CDCl_3$) δ 154.76, 143.50, 136.79, 135.42, 128.97, 126.57, 121.78, 114.03, 55.51, 52.11, 46.61, 21.10, 10.14, 3.89, 3.01. HRMS calculated for $C_{19}H_{22}NO$: 280.1701, observed: 280.1713 $[M+H]^+$. Enantioselectivity was determined by SFC analysis (Chiralcel-OJ-3, 2.5 mL/min, 254 nm, CO_2/MeOH, 95/5); t_{R1} = 2.4 min, t_{R2} = 2.8 min.

(2*S*,3*S*)-2-(4-(*tert*-butyl)phenyl)-3-cyclopropyl-1-(4-methoxyphenyl)aziridine (3c)

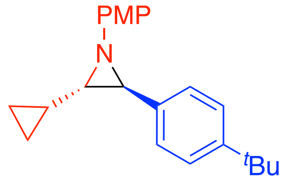

The reaction was performed following General Procedure A with (E)-1-cyclopropyl-N-(4-methoxyphenyl)methanimine (**1a**) and 1-(*tert*-butyl)-4-(chloromethyl)benzene (**2c**). The crude product was purified by flash chromatography on deactivated silica gel (eluted with hexanes: ethyl acetate = 98:2) to give the product as a pale-yellow oil (31.8 mg, 99% yield, 98% ee). ^1H NMR (400 MHz, $CDCl_3$) δ 7.41 – 7.34 (d, J = 8.9 Hz, 2H), 7.24 (d, J = 8.9 Hz, 2H), 7.02 – 6.94 (d, J = 8.1 Hz, 2H), 6.86 – 6.78 (d, J = 8.1 Hz, 2H), 3.80 (s, 3H), 3.12 (d, J = 3.1 Hz, 1H), 2.14 (dd, J = 7.2, 3.1 Hz, 1H), 1.35 (s, 9H), 0.68 (ddd, J = 8.6, 7.1, 4.4 Hz, 1H), 0.53 (m, 3H), 0.49 – 0.35 (m, 1H). $^{13}C\{^1H\}$ NMR (101 MHz, $CDCl_3$) δ 154.77, 150.13, 143.61, 135.68, 126.29, 125.24, 121.81, 114.04, 55.52, 52.35, 46.55, 34.52, 31.40, 10.08, 4.04, 3.03. HRMS calculated for $C_{22}H_{27}NO$: 321.2093, observed: 321.2094 $[M]^+$. Enantioselectivity was determined by SFC analysis (Chiralcel-IA-3, 2.5 mL/min, 254 nm, CO_2/MeOH, 97.5/2.5); t_{R1} = 2.4 min, t_{R2} = 7.2 min.

(2*S*,3*S*)-2-cyclopropyl-1-(4-methoxyphenyl)-3-(4-(methylthio)phenyl)aziridine (3d)

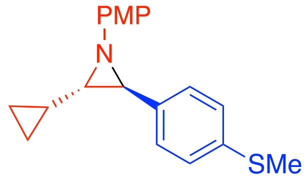

The reaction was performed following General Procedure A with (E)-1-cyclopropyl-N-(4-methoxyphenyl)methanimine (**1a**) and (4-(chloromethyl)phenyl)(methyl)sulfane (**2d**). The crude product was purified by flash chromatography on deactivated silica gel (eluted with hexanes: ethyl acetate = 98:2) to give the product as a pale-yellow oil (28.8 mg, 92% yield, 95% ee). ^1H NMR (400 MHz, $CDCl_3$) δ 7.27 – 7.15 (m, 4H), 6.98 – 6.89 (m, 2H), 6.87 – 6.74 (m, 2H), 3.79 (s, 3H), 3.10 (d, J = 3.0 Hz, 1H), 2.49 (s, 3H), 2.16 – 2.09 (dd, J = 7.6 Hz, J = 2.8 Hz, 1H), 0.74 – 0.62 (m, 1H), 0.62 – 0.46 (m, 3H), 0.48 – 0.36 (m, 1H). $^{13}C\{^1H\}$ NMR (101 MHz, $CDCl_3$) δ 154.84, 143.28, 137.10, 135.51, 127.17, 126.76, 121.75, 114.09, 55.51, 52.25, 46.38, 16.13, 10.14, 3.91, 3.05. HRMS calculated for $C_{19}H_{22}NOS$: 312.1422, observed: 312.1398 $[M+H]^+$. Enantioselectivity was determined by SFC analysis (Chiralcel-OJ-3, 2.5 mL/min, 254 nm, CO_2/MeOH, 90/10); t_{R1} = 3.1 min, t_{R2} = 3.4 min.

(2*S*,3*S*)-2-cyclopropyl-1-(4-methoxyphenyl)-3-(4-phenethylphenyl)aziridine (3e)

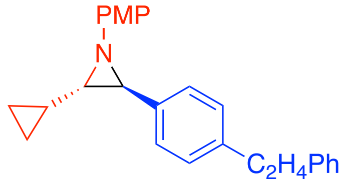

The reaction was performed following General Procedure A with (E)-1-cyclopropyl-N-(4-methoxyphenyl)methanimine (**1a**) and 1-(chloromethyl)-4-phenethylbenzene (**2e**). The crude product was purified by flash chromatography on deactivated silica gel (eluted with hexanes: ethyl acetate = 98:2) to give the product as a pale-yellow oil (34.8 mg, 94% yield, 95% ee). ^1H NMR (400 MHz, $CDCl_3$) δ 7.32 (m, 2H), 7.28 – 7.18 (m, 5H), 7.16 (d, J = 8.2 Hz, 2H), 7.00 – 6.92 (d, J = 8.2 Hz, 2H), 6.86 – 6.78 (m, 2H), 3.80 (s, 3H), 3.13 (d, J = 3.1 Hz, 1H), 2.94 (s, 4H), 2.20 – 2.13 (m, 1H), 0.71 – 0.66 (m, 1H), 0.59 – 0.50 (m, 3H), 0.45 – 0.39 (m,1H). $^{13}C\{^1H\}$ NMR (101 MHz, $CDCl_3$) δ 154.80, 143.48, 141.77, 140.76, 136.02, 128.47,

128.40, 128.36, 126.70, 125.94, 121.82, 114.05, 55.52, 52.11, 46.63, 37.94, 37.60, 10.16, 3.93, 3.05. HRMS calculated for $C_{26}H_{28}NO$: 370.2171, observed: 370.2165 $[M+H]^+$. Enantioselectivity was determined by SFC analysis (Chiralcel-IA-3, 2.5 mL/min, 254 nm, CO_2/MeOH, 90/10); t_{R1} = 2.8 min, t_{R2} = 7.4 min.

(2*S*,3*S*)-2-cyclopropyl-3-mesityl-1-(4-methoxyphenyl)aziridine (3f)

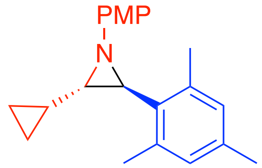

The reaction was performed following General Procedure A with (E)-1-cyclopropyl-N-(4-methoxyphenyl)methanimine (**1a**) and 2-(chloromethyl)-1,3,5-trimethylbenzene (**2f**). The crude product was purified by flash chromatography on deactivated silica gel (eluted with hexanes: ethyl acetate = 98:2) to give the product as a white solid (26.2 mg, 85% yield, 93% *ee*). ^{1}H NMR (400 MHz, $CDCl_3$) δ 7.16 – 7.07 (m, 2H), 6.94 – 6.83 (m, 4H), 3.83 (s, 3H), 3.12 (d, *J* = 3.0 Hz, 1H), 2.44 (s, 6H), 2.29 (s, 3H), 1.80 (dd, *J* = 9.4, 3.1 Hz, 1H), 0.71 – 0.56 (m, 2H), 0.56 – 0.42 (m, 2H), 0.23 – 0.10 (m, 1H). $^{13}C\{^1H\}$ NMR (101 MHz, $CDCl_3$) δ 154.94, 143.35, 137.38, 136.20, 132.20, 129.02, 122.58, 114.00, 55.52, 52.16, 44.20, 20.85, 20.12, 9.82, 4.03, 3.34. HRMS calculated for $C_{21}H_{26}NO$: 308.2014, observed: 308.2024 $[M+H]^+$. Enantioselectivity was determined by SFC analysis (Chiralcel-IF-3, 2.5 mL/min, 254 nm, CO_2/MeOH, 95/5); t_{R1} = 2.5 min, t_{R2} = 3.6 min.

(2*S*,3*S*)-2-cyclopropyl-1-(4-methoxyphenyl)-3-(4-phenoxyphenyl)aziridine (3g)

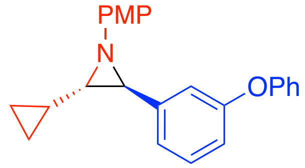

The reaction was performed following General Procedure A with (E)-1-cyclopropyl-N-(4-methoxyphenyl)methanimine (**1a**) and 1-(chloromethyl)-4-phenoxybenzene (**2g**). The crude product was purified by flash chromatography on deactivated silica gel (eluted with hexanes: ethyl acetate = 98:2) to give the product as a white solid (35.0 mg, 98% yield, 97% *ee*). ^{1}H NMR (400 MHz, $CDCl_3$) δ 7.42 – 7.24 (m, 3H), 7.16 – 7.03 (m, 2H), 7.03 – 6.87 (m, 6H), 6.87 – 6.71 (m, 2H), 3.80 (s, 3H), 3.13 (d, *J* = 3.0 Hz, 1H), 2.23 – 2.07 (m, 1H), 0.74 – 0.63 (m, 1H), 0.59 – 0.49 (m, 3H), 0.45 – 0.40 (m, 1H). $^{13}C\{^1H\}$ NMR (101 MHz, $CDCl_3$) δ 157.25, 157.21, 154.90, 143.18, 140.63, 129.74, 129.62, 123.16, 121.85, 121.75, 118.78, 117.74, 117.16, 114.11, 55.51, 52.08, 46.52, 10.14, 3.89, 3.09. HRMS calculated for $C_{24}H_{24}NO_2$: 358.1807, observed: 358.1800 $[M+H]^+$. Enantioselectivity was determined by SFC analysis of its ring opening product **3ga** and **3gb** (Chiralcel-IC-3, 2.5 mL/min, 254 nm, CO_2/MeOH, 90/10); t_{R1} = 4.0 min, t_{R2} = 4.7 min, t_{R3} = 5.9 min, t_{R4} = 6.5 min.

(2*S*,3*S*)-2-cyclopropyl-1-(4-methoxyphenyl)-3-(3-(trifluoromethyl)phenyl)aziridine (3h)

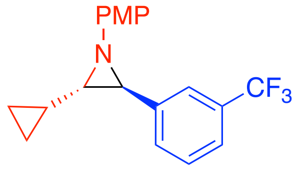

The reaction was performed following General Procedure A with (E)-1-cyclopropyl-N-(4-methoxyphenyl)methanimine (**1a**) and 1-(chloromethyl)-3-(trifluoromethyl)benzene (**2h**). The crude product was purified by flash chromatography on deactivated silica gel (eluted with hexanes: ethyl acetate = 98:2) to give the product as a pale-yellow oil (27.1 mg, 81% yield, 84% *ee*). ^{1}H NMR (400 MHz, $CDCl_3$) δ 7.61 (s, 1H), 7.52 (dd, *J* = 14.5, 7.7 Hz, 2H), 7.45 (t, *J* = 7.6 Hz, 1H), 7.03 – 6.95 (m, 2H), 6.89 – 6.79 (m, 2H), 3.80 (s, 3H), 3.18 (d, *J* = 2.9 Hz, 1H), 2.10 (dd, *J* = 7.7, 2.9 Hz, 1H), 0.77 – 0.66 (m, 1H), 0.65 – 0.38 (m, 4H). $^{19}F\{^1H\}$ NMR (376 MHz, $CDCl_3$) δ -62.55. $^{13}C\{^1H\}$ NMR (101 MHz, $CDCl_3$) δ 155.09, 142.95, 140.16, 130.78 (q, *J* = 32 Hz), 129.76, 128.75, 124.18 (q, *J* = 273 Hz), 123.88 (q, *J* = 3.6 Hz), 123.28 (q, *J* = 3.6 Hz), 121.71, 114.21, 55.52, 53.15, 46.14, 9.88, 4.21, 3.07. HRMS calculated for $C_{19}H_{19}F_3NO$: 334.1419, observed:

334.1424 [M+H]+. Enantioselectivity was determined by SFC analysis (Chiralcel-OJ-3, 2.5 mL/min, 254 nm, CO_2/MeOH, 97.5/2.5); t_{R1} = 1.3 min, t_{R2} = 1.6 min.

(2*S*,3*S*)-2-cyclopropyl-1-(4-methoxyphenyl)-3-(4-(trifluoromethoxy)phenyl)aziridine (3i)

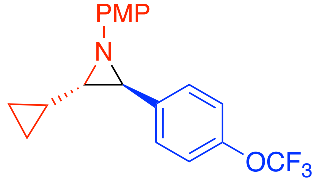

The reaction was performed following General Procedure A with (E)-1-cyclopropyl-N-(4-methoxyphenyl)methanimine (**1a**) and 1-(chloromethyl)-4-(trifluoromethoxy)benzene (**2i**). The crude product was purified by flash chromatography on deactivated silica gel (eluted with hexanes: ethyl acetate = 98:2) to give the product as a white solid (28.5 mg, 81% yield, 97% *ee*). ^{1}H NMR (400 MHz, $CDCl_3$) δ 7.36 – 7.25 (m, 2H), 7.22 – 7.14 (m, 2H), 7.00 – 6.92 (m, 2H), 6.86 – 6.77 (m, 2H), 3.79 (s, 3H), 3.12 (d, *J* = 3.0 Hz, 1H), 2.08 (dd, *J* = 7.6, 3.0 Hz, 1H), 0.75 – 0.63 (m, 1H), 0.60 – 0.46 (m, 3H), 0.45 – 0.39 (m, 1H). ^{19}F{^{1}H} NMR (376 MHz, $CDCl_3$) δ -57.92. ^{13}C{^{1}H} NMR (101 MHz, $CDCl_3$) δ 154.99, 148.34 (q, *J* = 2 Hz), 143.05, 137.61, 127.80, 121.69, 120.90, 120.50 (q, *J* = 258 Hz), 114.14, 55.50, 52.77, 45.92, 9.93, 4.08, 3.03. HRMS calculated for $C_{19}H_{19}F_3NO_2$: 350.1368, observed: 350.1381 [M+H]+. Enantioselectivity was determined by SFC analysis (Chiralcel-OJ-3, 2.5 mL/min, 254 nm, CO_2/MeOH, 95/5); t_{R1} = 2.2 min, t_{R2} = 2.5 min.

(2*S*,3*S*)-2-(3-bromophenyl)-3-cyclopropyl-1-(4-methoxyphenyl)aziridine (3j)

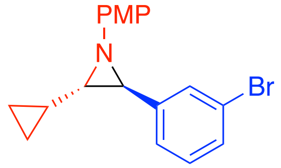

The reaction was performed following General Procedure A with (E)-1-cyclopropyl-N-(4-methoxyphenyl)methanimine (**1a**) and 1-bromo-3-(chloromethyl)benzene (**2j**). The crude product was purified by flash chromatography on deactivated silica gel (eluted with hexanes: ethyl acetate = 98:2) to give the product as a white solid (30.2 mg, 88% yield, 90% *ee*). ^{1}H NMR (400 MHz, $CDCl_3$) δ 7.49 (t, *J* = 1.8 Hz, 1H), 7.40 (dt, *J* = 7.6, 1.7 Hz, 1H), 7.27 – 7.13 (m, 2H), 7.01 – 6.93 (m, 2H), 6.87 – 6.77 (m, 2H), 3.79 (s, 3H), 3.07 (d, *J* = 2.9 Hz, 1H), 2.06 (dd, *J* = 7.7, 3.0 Hz, 1H), 0.72 – 0.76 (m, 1H), 0.62 – 0.36 (m, 4H). ^{13}C{^{1}H} NMR (101 MHz, $CDCl_3$) δ 155.01, 143.02, 141.47, 130.17, 129.83, 129.48, 125.10, 122.60, 121.69, 114.16, 55.53, 52.98, 46.01, 9.85, 4.22, 3.05. HRMS calculated for $C_{18}H_{19}BrNO$: 344.0650, observed: 344.0645 [M+H]+. Enantioselectivity was determined by SFC analysis (Chiralcel-OJ-3, 2.5 mL/min, 254 nm, CO_2/MeOH, 95/5); t_{R1} = 3.0 min, t_{R2} = 3.3 min.

(2*S*,3*S*)-2-(2-bromophenyl)-3-cyclopropyl-1-(4-methoxyphenyl)aziridine (3k)

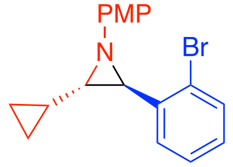

The reaction was performed following General Procedure A with (E)-1-cyclopropyl-N-(4-methoxyphenyl)methanimine (**1a**) and 1-bromo-2-(chloromethyl)benzene (**2k**). The crude product was purified by flash chromatography on deactivated silica gel (eluted with hexanes: ethyl acetate = 98:2) to give the product as a pale-yellow oil (25.1 mg, 73% yield, 73% *ee*). ^{1}H NMR (400 MHz, $CDCl_3$) δ 7.58 (dd, *J* = 7.9, 1.2 Hz, 1H), 7.42 (dd, *J* = 7.7, 1.8 Hz, 1H), 7.35 – 7.24 (m, 1H), 7.15 (td, *J* = 7.7, 1.8 Hz, 1H), 7.09 – 7.00 (m, 2H), 6.89 – 6.81 (m, 2H), 3.81 (s, 3H), 3.37 (d, *J* = 3.0 Hz, 1H), 1.99 (dd, *J* = 7.8, 3.0 Hz, 1H), 0.76 – 0.64 (m, 1H), 0.64 – 0.48 (m, 2H), 0.52 – 0.39 (m, 2H). ^{13}C{^{1}H} NMR (101 MHz, $CDCl_3$) δ 155.02, 143.36, 138.59, 132.16, 128.52, 127.81, 127.52, 123.59, 121.76, 114.17, 55.55, 52.66, 46.90, 9.62, 4.43, 3.28. HRMS calculated for $C_{18}H_{19}BrNO$: 344.0650, observed: 344.0625 [M+H]+. Enantioselectivity was

determined by SFC analysis (Chiralcel-IA-3, 2.5 mL/min, 254 nm, CO_2/MeOH, 95/5); t_{R1} = 2.2 min, t_{R2} = 3.2 min.

(2*S*,3*S*)-2-cyclopropyl-3-(4-fluorophenyl)-1-(4-methoxyphenyl)aziridine (3l)

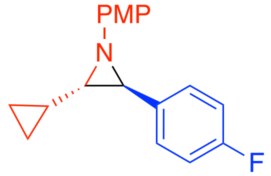

The reaction was performed following General Procedure A with (E)-1-cyclopropyl-N-(4-methoxyphenyl)methanimine (**1a**) and 1-(chloromethyl)-4-fluorobenzene (**2l**). The crude product was purified by flash chromatography on deactivated silica gel (eluted with hexanes: ethyl acetate = 98:2) to give the product as a white solid (27.2 mg, 96% yield, >99% *ee*). ^{1}H NMR (400 MHz, $CDCl_3$) δ 7.28 – 7.19 (m, 2H), 7.08 – 6.88 (m, 4H), 6.85 – 6.76 (m, 2H), 3.79 (s, 3H), 3.11 (d, *J* = 3.0 Hz, 1H), 2.09 (dd, *J* = 7.2, 3.0 Hz, 1H), 0.74 – 0.62 (m, 1H), 0.62 – 0.46 (m, 3H), 0.44 – 0.38 (m, 1H). ^{19}F{^{1}H} NMR (376 MHz, $CDCl_3$) δ -115.62. ^{13}C{^{1}H} NMR (101 MHz, $CDCl_3$) δ 162.13 (d, *J* = 245 Hz), 154.90, 143.18, 134.24, 128.12 (d, *J* = 8 Hz), 121.74, 115.15 (d, *J* = 22 Hz), 114.11, 55.51, 52.26, 46.04, 10.03, 3.95, 3.04. HRMS calculated for $C_{18}H_{18}FNO$: 283.1372, observed: 283.1378 $[M]^+$. Enantioselectivity was determined by SFC analysis (Chiralcel-OJ-3, 2.5 mL/min, 254 nm, CO_2/MeOH, 99/1); t_{R1} = 5.3 min, t_{R2} = 6.5 min.

(2*S*,3*S*)-2-cyclopropyl-3-(2-fluorophenyl)-1-(4-methoxyphenyl)aziridine (3m)

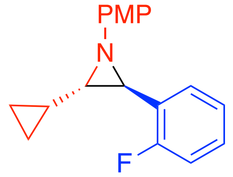

The reaction was performed following General Procedure A with (E)-1-cyclopropyl-N-(4-methoxyphenyl)methanimine (**1a**) and 1-(chloromethyl)-2-fluorobenzene (**2m**). The crude product was purified by flash chromatography on deactivated silica gel (eluted with hexanes: ethyl acetate = 98:2) to give the product as a pale-yellow oil (26.4 mg, 94% yield, 95% *ee*). ^{1}H NMR (400 MHz, $CDCl_3$) δ 7.26 – 7.19 (m, 2H), 7.09 (td, *J* = 8.2, 1.1 Hz, 2H), 7.05 – 6.97 (m, 2H), 6.87 – 6.77 (m, 2H), 3.79 (s, 3H), 3.38 (d, *J* = 3.0 Hz, 1H), 2.16 (dd, *J* = 7.3, 3.1 Hz, 1H), 0.75 – 0.64 (m, 1H), 0.62 – 0.36 (m, 4H). ^{19}F{^{1}H} NMR (376 MHz, $CDCl_3$) δ -120.35. ^{13}C{^{1}H} NMR (101 MHz, $CDCl_3$) δ 161.77f (d, *J* = 246 Hz), 154.98, 143.26, 128.44 (d, *J* = 8 Hz), 127.51 (d, *J* = 4 Hz), 126.02 (d, *J* = 13 Hz), 124.11 (d, *J* = 3 Hz), 121.80, 114.99 (d, *J* = 21 Hz), 114.11, 55.51, 51.50, 40.30 (d, *J* = 19 Hz), 9.74, 4.16, 3.06. HRMS calculated for $C_{18}H_{18}FNO$: 283.1372, observed: 283.1383 $[M]^+$. Enantioselectivity was determined by SFC analysis (Chiralcel-OJ-3, 2.5 mL/min, 254 nm, CO_2/MeOH, 97.5/2.5); t_{R1} = 1.9 min, t_{R2} = 2.2 min.

(2*S*,3*S*)-2-(4-chlorophenyl)-3-cyclopropyl-1-(4-methoxyphenyl)aziridine (3n)

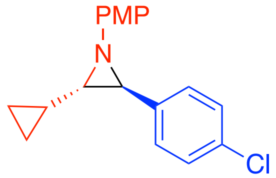

The reaction was performed following General Procedure A with (E)-1-cyclopropyl-N-(4-methoxyphenyl)methanimine (**1a**) and 1-chloro-4-(chloromethyl)benzene (**2n**). The crude product was purified by flash chromatography on deactivated silica gel (eluted with hexanes: ethyl acetate = 98:2) to give the product as a white solid (21.3 mg, 77% yield, 95% *ee*). ^{1}H NMR (400 MHz, $CDCl_3$) δ 7.34 – 7.26 (m, 2H), 7.22 (d, *J* = 8.5 Hz, 2H), 6.95 (d, *J* = 9.0 Hz, 2H), 6.82 (d, *J* = 9.0 Hz, 2H), 3.79 (s, 3H), 3.11 (d, *J* = 3.0 Hz, 1H), 2.10 (dd, *J* = 7.2, 3.0 Hz, 1H), 0.71 – 0.66 (m, 1H), 0.63 – 0.46 (m, 3H), 0.45 – 0.39 (m, 1H). ^{13}C{^{1}H} NMR (101 MHz, $CDCl_3$) δ 154.96, 143.07, 137.21, 132.85, 128.43, 127.94, 121.71, 114.15, 55.51, 52.55, 46.05, 10.04, 4.01, 3.08. HRMS calculated for $C_{18}H_{19}ClNO$: 300.1155, observed: 300.1185 $[M+H]^+$.

Enantioselectivity was determined by SFC analysis (Chiralcel-OJ-3, 2.5 mL/min, 254 nm, CO_2/MeOH, 95/5); t_{R1} = 3.0 min, t_{R2} = 3.4 min.

(2*S*,3*S*)-2-cyclopropyl-1-(4-methoxyphenyl)-3-(naphthalen-1-yl)aziridine (3o)

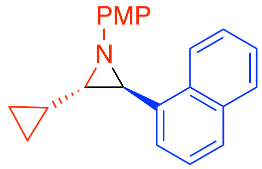

The reaction was performed following General Procedure A with (E)-1-cyclopropyl-N-(4-methoxyphenyl)methanimine (**1a**) and 1-(chloromethyl)naphthalene (**2o**). The crude product was purified by flash chromatography on deactivated silica gel (eluted with hexanes: ethyl acetate = 98:2) to give the product as a white solid (16.1 mg, 51% yield, 92% *ee*). ^{1}H NMR (400 MHz, $CDCl_3$) δ 8.31 (d, *J* = 8.3 Hz, 1H), 7.99 – 7.92 (m, 1H), 7.83 (d, *J* = 8.2 Hz, 1H), 7.72 (d, *J* = 7.0 Hz, 1H), 7.68 – 7.51 (m, 2H), 7.50 (t, *J* = 7.7 Hz, 1H), 7.19 – 7.11 (m, 2H), 6.94 – 6.86 (m, 2H), 3.84 (s, 3H), 3.69 (d, *J* = 3.2 Hz, 1H), 2.03 (dd, *J* = 8.1, 3.4 Hz, 1H), 0.86 – 0.69 (m, 2H), 0.63 – 0.50 (m, 3H). ^{13}C{^{1}H} NMR (101 MHz, $CDCl_3$) δ 155.03, 143.96, 135.16, 133.47, 131.91, 128.78, 127.47, 126.16, 125.80, 125.76, 123.70, 123.41, 121.84, 114.25, 55.58, 52.28, 45.34, 9.93, 4.49, 2.98. HRMS calculated for $C_{22}H_{22}NO$: 316.1701, observed: 316.1711 $[M+H]^+$. Enantioselectivity was determined by SFC analysis (Chiralcel-OJ-3, 2.5 mL/min, 254 nm, CO_2/MeOH, 95/5); t_{R1} = 3.5 min, t_{R2} = 5.4 min.

(2*S*,3*S*)-2-cyclopropyl-1-(4-methoxyphenyl)-3-(4-vinylphenyl)aziridine (3p)

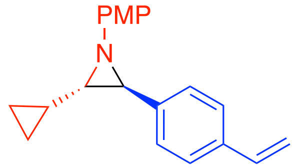

The reaction was performed following General Procedure A with (E)-1-cyclopropyl-N-(4-methoxyphenyl)methanimine (**1a**) and 1-(chloromethyl)-4-vinylbenzene (**2p**). The crude product was purified by flash chromatography on deactivated silica gel (eluted with hexanes: ethyl acetate = 98:2) to give the product as a pale yellow oil (27.1 mg, 93% yield, 96% *ee*). ^{1}H NMR (400 MHz, $CDCl_3$) δ 7.46 – 7.34 (m, 2H), 7.23 (d, *J* = 7.9 Hz, 2H), 7.02 – 6.91 (m, 2H), 6.86 – 6.77 (m, 2H), 6.72 (dd, *J* = 17.6, 10.9 Hz, 1H), 5.75 (dd, *J* = 17.6, 1.0 Hz, 1H), 5.25 (dd, *J* = 10.9, 1.0 Hz, 1H), 3.79 (s, 3H), 3.13 (d, *J* = 3.0 Hz, 1H), 2.18 – 2.11 (m, 1H), 0.70 – 0.64 (m, 1H), 0.59 – 0.48 (m, 3H), 0.47 – 0.37 (m, 1H). ^{13}C{^{1}H} NMR (101 MHz, $CDCl_3$) δ 154.84, 143.34, 138.15, 136.56, 136.53, 126.82, 126.14, 121.75, 114.08, 113.56, 55.51, 52.38, 46.60, 10.15, 3.92, 3.05. HRMS calculated for $C_{20}H_{22}NO$: 292.1701, observed: 292.1714 $[M+H]^+$. Enantioselectivity was determined by SFC analysis (Chiralcel-OJ-3, 2.5 mL/min, 254 nm, CO_2/MeOH, 97.5/2.5); t_{R1} = 5.6 min, t_{R2} = 6.5 min.

2-(3-((2*S*,3*S*)-3-cyclopropyl-1-(4-methoxyphenyl)aziridin-2-yl)phenyl)pyrimidine (3q)

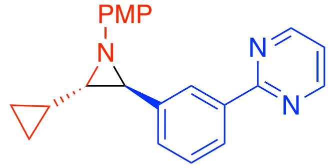

The reaction was performed following General Procedure A with (E)-1-cyclopropyl-N-(4-methoxyphenyl)methanimine (**1a**) and 2-(3-(chloromethyl)phenyl)pyrimidine (**2q**). The crude product was purified by flash chromatography on deactivated silica gel (eluted with hexanes: ethyl acetate = 98:2) to give the product as a pale yellow solid (34.0 mg, 50% yield, 73% *ee*). ^{1}H NMR (400 MHz, $CDCl_3$) δ 8.82 (d, *J* = 4.9 Hz, 2H), 8.44 (t, *J* = 1.8 Hz, 1H), 8.33 (dt, *J* = 7.7, 1.5 Hz, 1H), 7.43 (t, *J* = 7.7 Hz, 1H), 7.35 (d, *J* = 7.7 Hz, 1H), 7.20 (t, *J* = 4.8 Hz, 1H), 7.07 – 6.93 (m, 2H), 6.87 – 6.74 (m, 2H), 3.77 (s, 3H), 3.22 (d, *J* = 3.0 Hz, 1H), 2.17 (dd, *J* = 7.6, 2.9 Hz, 1H), 0.74 – 0.62 (m, 1H), 0.58 – 0.36 (m, 4H). ^{13}C{^{1}H} NMR (101 MHz, $CDCl_3$) δ 164.66, 157.24, 154.84, 143.47, 139.42, 137.64, 128.72, 128.48, 127.00, 126.72, 121.82, 119.15, 114.07, 55.52, 52.67, 46.76, 10.02, 4.14, 3.07. HRMS calculated for $C_{22}H_{22}N_3O$: 344.1763, observed:

344.1768 [M+H]$^+$. Enantioselectivity was determined by SFC analysis (Chiralcel-OJ-3, 2.5 mL/min, 254 nm, CO_2/MeOH, 95/5); t_{R1} = 10.3 min, t_{R2} = 12.0 min.

(2*S*,3*S*)-2-cyclopropyl-1-(4-methoxyphenyl)-3-vinylaziridine (3r)

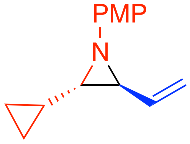

The reaction was performed following a slightly modified General Procedure A, with the modification being use of 1 equiv. of (E)-1-cyclopropyl-N-(4-methoxyphenyl)methanimine (**1a**) and 4 equiv. of 3-chloroprop-1-ene (**2r**). The crude product was purified by flash chromatography on deactivated silica gel (eluted with hexanes: ethyl acetate = 98:2) to give the product as a pale-yellow oil (12.5 mg, 58% yield, 89% *ee*). ^{1}H NMR (400 MHz, $CDCl_3$) δ 6.88 – 6.79 (m, 2H), 6.82 – 6.71 (m, 2H), 5.47 – 5.38 (m, 1H), 5.25 – 5.13 (m, 2H), 3.75 (s, 3H), 2.78 – 2.71 (m, 1H), 2.16 – 2.04 (m, 1H), 0.87 (m, 1H), 0.62 – 0.33 (m, 4H). ^{13}C{^{1}H} NMR (101 MHz, $CDCl_3$) δ 154.70, 143.48, 135.80, 121.41, 117.78, 113.97, 55.38, 48.55, 46.13, 10.77, 3.01, 2.14. HRMS calculated for $C_{14}H_{18}NO$: 216.1388, observed: 216.1375 [M+H]$^+$. Enantioselectivity was determined by SFC analysis (Chiralcel-OJ-3, 2.5 mL/min, 254 nm, CO_2/MeOH, 97.5/2.5); t_{R1} = 1.4 min, t_{R2} = 1.8 min.

Procedure for the gram-scale synthesis of (2*S*,3*S*)-2-cyclopropyl-3-(4-fluorophenyl)-1-(4-methoxyphenyl)aziridine (3l)

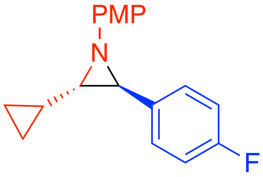

To an oven-dried 40 mL reaction vial equipped with a stir bar was brought into the nitrogen filled glovebox. To the vial was added (E)-1-cyclopropyl-N-(4-methoxyphenyl)methanimine (**1a**, 12 mmol, 2.0 equiv., 1.05 g), sulfoxide pre-catalyst (**A2**, 0.6 mmol, 216 mg), $LiN(SiMe_3)_2$ (2.01 g, 12 mmol, 2.0 equiv.), and CPME (15 mL) at room temperature. Next, 1-(chloromethyl)-4-fluorobenzene (**2l**, 6 mmol, 1.0 equiv., 719 uL) was added through pipette. The reaction vial was capped with a screw cap with septum and transferred out of the glove box. The reaction mixture was heated at 45 °C and stirred for 24 h. The resulting cloudy yellow reaction mixture was cooled to room temperature and opened to air. Water (0.5 mL) was added to the reaction solution, and it was stirred at room temperature for 5 min. The reaction mixture was vacuumed filtered through a plug of Celite packed in a 15 mL Buchner funnel into a 250 mL round bottom flask. The pad was then rinsed with ethyl acetate (30 mL x 2) and the solvent was removed under reduced pressure. The crude product was purified by flash chromatography on silica gel (deactivated with triethylamine by flashing 3% triethylamine in hexanes through the column). The product was eluted with hexanes : ethyl acetate = 98:2) to give the product (1.62 g, 95% yield, 99% *ee*) as a white solid.

Procedure of synthesizing aziridine ring-opening product

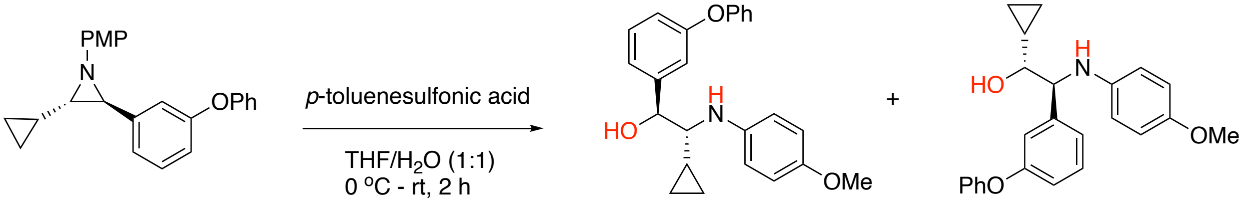

3g **3ga** (major) **3gb** (minor)

***(1S, 2R)*-2-cyclopropyl-2-((4-methoxyphenyl)amino)-1-(3-phenoxyphenyl)ethan-1-ol (3ga) and *(1R, 2S)*-1-cyclopropyl-2-((4-methoxyphenyl)amino)-2-(3-phenoxyphenyl)ethan-1-ol (3gb)**: Following a modified procedure,[1] to a precooled (0 °C) solution of aziridine **3g** (172.0 mg, 0.48 mmol, 1.0 equiv.) in THF/H_2O (v:v = 1:1, 2.4 mL) was added *p*-toluenesulfonic acid (92.3 mg, 0.48 mmol, 1.0 equiv.) under air at 0 °C in an ice bath. The solution was stirred at 0 °C for 30 min and then slowly warmed up to room temperature and kept stirring for 2 h at room temperature. The color of the solution changed from colorless to orange. Next, 24 mL water was added to the resulting orange solution, which was extracted with Et_2O (2 x 20 mL) and separated. The organic layer was collected, dried over Na_2SO_4 and filtered. The volatile materials were removed under reduced pressure. The crude product was purified by flash chromatography on silica gel (eluted with hexanes : ethyl acetate = 9:1) to give a non-separable mixture of **3ga** and **3gb** (170 mg, 94%, 97% *ee*) at a ratio of 7:4 as a white solid. Enantioselectivity was determined by SFC analysis (Chiralcel-IC-3, 2.5 mL/min, 254 nm, CO_2/MeOH, 90/10); t_{R1} = 4.0 min, t_{R2} = 4.7 min, t_{R3} = 5.9 min, t_{R4} = 6.5 min. This result was utilized to determine the enantioselectivity of **3g**, as no suitable separation method was identified for **3g** using SFC. **3ga**: ^{1}H NMR (400 MHz, $CDCl_3$) δ 7.44 – 7.36 (m, 3H), 7.26 – 7.21 (m, 1H), 7.21 – 7.14 (m, 2H), 7.10 – 7.04 (m, 2H), 7.04 – 6.99 (m, 1H), 6.85 (d, *J* = 8.9 Hz, 2H), 6.74 (d, *J* = 8.9 Hz, 2H), 5.04 (d, *J* = 3.5 Hz, 1H), 3.83 (s, 3H), 2.92 (dd, *J* = 8.8, 3.6 Hz, 1H), 0.88 – 0.84 (dt, *J* = 8.5, 5.0 Hz, 1H), 0.52 – 0.33 (m, 2H), 0.25 – 0.12 (m, 1H), 0.02 – 0.01 (m, 1H). ^{13}C{^{1}H} NMR (101 MHz, $CDCl_3$) δ 157.50, 157.01, 152.98, 143.53, 141.68, 129.76, 129.36, 123.15, 121.35, 118.64, 117.99, 117.30, 116.24, 114.92, 73.87, 65.95, 55.76, 11.99, 4.10, 2.67. HRMS calculated for $C_{24}H_{26}NO_3$: 376.1913, observed: 376.1904 [M+H]$^+$. **3gb**: ^{1}H NMR (400 MHz, $CDCl_3$) δ 7.37 – 7.25 (m, 3H), 7.19 – 7.17 (m, 1H), 7.13 – 7.04 (m, 2H), 6.98 – 6.84 (m, 3H), 6.76 – 6.67 (m, 2H), 6.58 – 6.48 (m, 2H), 4.49 (d, *J* = 3.5 Hz, 1H), 3.73 (s, 3H), 3.28 (dd, *J* = 8.7, 3.5 Hz, 1H), 0.75 (dt, *J* = 13.3, 9.9 Hz, 1H), 0.58 – 0.47 (m, 2H), 0.42 – 0.30 (m, 1H), 0.25 (m, 1H). ^{13}C{^{1}H} NMR (101 MHz, $CDCl_3$) δ 157.28, 157.09, 152.22, 141.68, 141.00, 129.70, 129.60, 123.10, 123.02, 118.82, 118.59, 117.89, 115.20, 114.76, 78.85, 62.76, 55.74, 14.34, 3.12, 2.83. HRMS calculated for $C_{24}H_{26}NO_3$: 376.1913, observed: 376.1902 [M+H]$^+$.

Studies on reversible aziridine ring-opening/trapping

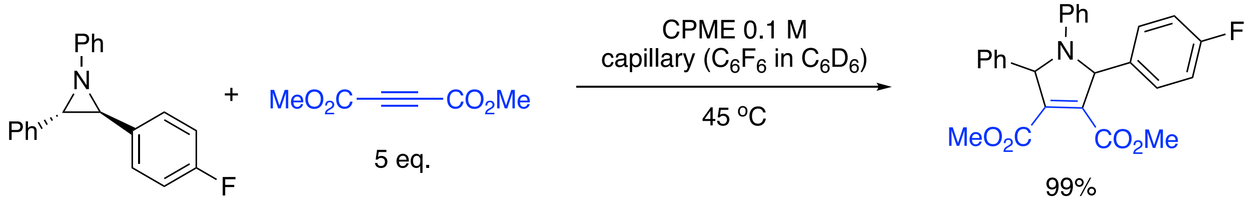

5 **6** **7**

To an oven-dried J-Young NMR tube was added *rac-trans*-2-(4-fluorophenyl)-1,3-diphenylaziridine **5** (14.5 mg, 0.05 mmol, 1 equiv.), dimethyl acetylenedicarboxylate **6** (31 uL, 0.25 mmol, 5 equiv.), CPME (0.5 mL) and a capillary of C_6F_6 in C_6D_6 as internal standard in the dry box under the nitrogen atmosphere. Reaction was heated at 45 °C and monitored by ^{19}F{^{1}H} NMR spectroscopy. After 20 h, full conversion of the aziridine **5** to the corresponding cycloadduct **7** was observed as shown in Scheme S1 (99% assay yield, determined by ^{19}F{^{1}H} NMR). ^{1}H NMR (400 MHz, $CDCl_3$) δ 7.58 – 7.46 (m, 4H), 7.36 (m, 2H), 7.29 (m, 1H), 7.09 – 6.96 (m, 4H), 6.72 – 6.64 (m, 1H), 6.58 – 6.49 (m, 2H), 5.75 (q, *J* = 4.0 Hz, 2H), 3.62 (d, *J* = 1.7 Hz, 6H). ^{13}C{^{1}H} NMR (101 MHz, $CDCl_3$) δ 163.75 (*J* = 245 Hz), 163.43 (*J* = 25 Hz), 146.46, 139.37, 138.32, 136.76, 135.51 (*J* = 3 Hz), 129.30 (*J* = 8 Hz), 128.98 (*J* = 19 Hz), 128.31, 127.38, 118.68, 115.79 (*J* = 21 Hz), 113.67, 72.23, 71.39, 52.37. ^{19}F{^{1}H} NMR (376 MHz, $CDCl_3$) δ -113.94. HRMS calculated for $C_{26}H_{23}FNO_4$: 432.1611, observed: 432.1604 [M+H]$^+$.

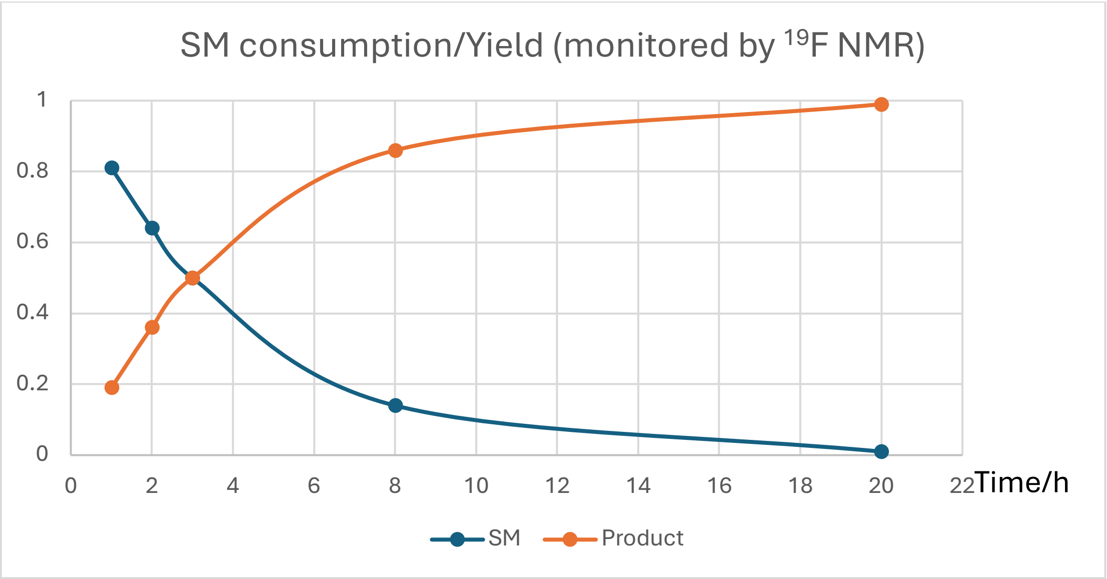

Scheme S1. Time course of ring-opening monitored by $^{19}F\{^1H\}$ NMR

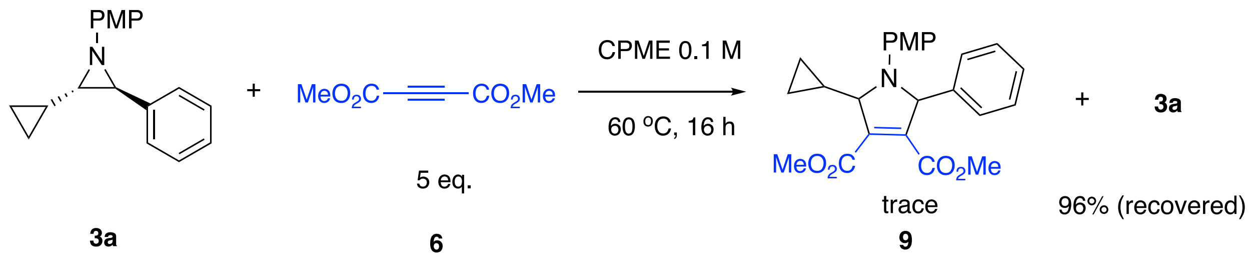

To an oven-dried 4 mL vial was added *rac-trans*-2-cyclopropyl-1-(4-methoxyphenyl)-3-phenylaziridine **3a** (26.6 mg, 0.1 mmol, 1 equiv.), dimethyl acetylenedicarboxylate **6** (62 uL, 0.5 mmol, 5 equiv.), and CPME (1.0 mL) under nitrogen atmosphere. The reaction was heated at 60 °C and stirred for 16 h. The reaction mixture was cooled to room temperature, opened to air, and the solvent was removed under reduced pressure. The crude product was purified by flash chromatography on deactivated silica gel (eluted with hexanes: ethyl acetate = 98:2) to recover the starting aziridine as a pale-yellow oil (25.5 mg, 96% yield). Only trace amount of the cycloadduct **9** was observed on HPLC-MS.

Crystallographic data of (2*S*,3*S*)-2-cyclopropyl-3-(4-fluorophenyl)-1-(4-methoxyphenyl)aziridine (3l)

Single crystals of aziridine **3l** (CCDC: 2422017)[6] was obtained through cooling the colorless clear solution of **3l** (50 mg) in hexanes (2 mL) from 40 ºC to -16 ºC over two days. A suitable crystal for X-ray analysis of **3l** was obtained and placed on the end of a Cryoloop coated in immersion oil.

Compound **3l** crystallizes in the orthorhombic space group $P2_12_12_1$ (systematic absences h00: h=odd, 0k0: k=odd, 00l: l=odd) with a=5.56376(3)Å, b=15.14867(7)Å, c=17.40929(10)Å, α=90°, β=90°, γ=90°, V=1467.317(13)Å^3, Z=4, and d_{calc}=1.283 g/cm^3. X-ray intensity data were collected on a Rigaku XtaLAB Synergy-S diffractometer [1] equipped with an HPC area detector (HyPix-6000HE) and employing confocal multilayer optic-monochromated Cu-Kα radiation (λ=1.54184 Å) at a temperature of 100K. Preliminary indexing was performed from a series of sixty 0.5° rotation frames with exposures of 0.25 seconds for θ = ±47.658° and 1 second for θ = 113.25°. A total of 9802 frames (136 runs) were collected employing ω scans with a crystal to detector distance of 34.0 mm, rotation widths of 0.5° and exposures of 0.25 seconds.

Rotation frames were integrated using CrysAlisPro [2], producing a listing of unaveraged F^2 and $\sigma(F^2)$ values. A total of 51050 reflections were measured over the ranges $7.736 \leq 2\theta \leq 148.968°$, $-6 \leq h \leq 6$, $-18 \leq k \leq 18$, $-21 \leq l \leq 18$ yielding 2966 unique reflections (R_{int} = 0.0381). The intensity data were corrected for Lorentz and polarization effects and for absorption using SCALE3 ABSPACK [3] (minimum and maximum transmission 0.88068, 1.00000). The structure was solved by dual space methods - SHELXT [4]. Refinement was by full-matrix least squares based on F^2 using SHELXL [5]. All reflections were used during refinement. The weighting scheme used was $w=1/[\sigma^2(F_o^2)+(0.0421P)^2 + 0.2240P]$ where $P = (F_o^2 + 2F_c^2)/3$. Non-hydrogen atoms were refined anisotropically and hydrogen atoms were refined using a riding model. Refinement converged to R1=0.0270 and wR2=0.0701 for 2932 observed reflections for which $F > 4\sigma(F)$ and R1=0.0278 and wR2=0.0724 and GOF =1.057 for all 2966 unique, non-zero reflections and 191 variables. The maximum Δ/σ in the final cycle of least squares was 0.001 and the two most prominent peaks in the final difference Fourier were +0.11 and -0.17 e/Å^3.

Table S1. Summary of Structure Determination of Compound 3l

Empirical formula	$C_{18}H_{18}FNO$
Formula weight	283.33
Diffractometer	Rigaku XtaLAB Synergy-S (HyPix-6000HE)
Temperature/K	100(2)
Crystal system	orthorhombic
Space group	$P2_12_12_1$
a	5.56376(3)Å
b	15.14867(7)Å
c	17.40929(10)Å
α	90°
β	90°
γ	90°
Volume	1467.317(13)Å^3
Z	4
d_{calc}	1.283 g/cm^3
μ	0.710 mm^{-1}
F(000)	600.0
Crystal size, mm	0.46 × 0.16 × 0.09
2θ range for data collection	7.736 - 148.968°
Index ranges	$-6 \leq h \leq 6$, $-18 \leq k \leq 18$, $-21 \leq l \leq 18$
Reflections collected	51050
Independent reflections	2966[R(int) = 0.0381]
Data/restraints/parameters	2966/0/191
Goodness-of-fit on F^2	1.057
Final R indexes [I>=2σ (I)]	R_1 = 0.0270, wR_2 = 0.0701
Final R indexes [all data]	R_1 = 0.0278, wR_2 = 0.0724
Largest diff. peak/hole	0.11/-0.17 eÅ^{-3}
Flack parameter	-0.02(3)

Reference:

[1] Z. Zheng, Y. Pu, J. Adrio, P. J. Walsh, *Angew. Chem. Int. Ed. Engl.* **2023**, *62*, e202303069.

[2] G. J. Rowlands, R. J. Seacome, *Beilstein J. Org. Chem.* **2009**, *5*, 9.

[3] D. J. Weix, J. A. Ellman, *Org. Lett.* **2003**, *5*, 1317-1320.

[4] P. B. Hitchcock, G. J. Rowlands, R. Parmar, *Chem. Commun. (Cambridge, U. K.)* **2005**, 4219-4221.

[5] D. Jishkariani, B. T. Diroll, M. Cargnello, D. R. Klein, L. A. Hough, C. B. Murray, B. Donnio, *J. Am. Chem. Soc.* **2015**, *137*, 10728-10734.

[6] Deposition Number 2422017 (**3l**) contains the supplementary crystallographic data for this paper. These data are provided free of charge by the joint Cambridge Crystallographic Data Centre and Fachinformationszentrum Karlsruhe Access Structures services.

SFC analysis

***R*-(*tert*-butyl) *tert*-butanethiosulfinate** (**S2**)

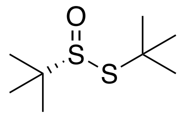

Chiral SFC conditions: Chiralcel-IG-3, 2.5 mL/min, 254 nm, CO_2/MeOH, 99/1)

S2, 86% *ee*

#	Time/Min	Area%
**1**	3.628	92.86%
**2**	5.015	7.14%

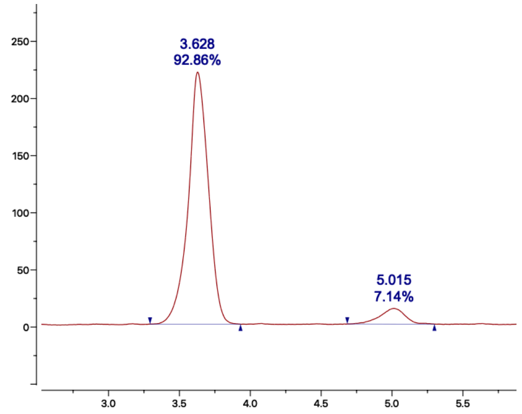

Figure S1. SFC spectrum of S2

(*S*P, *R*S)-(–)-4-*tert*-Butylsulfinyl[2.2]paracyclophane [(*S*P, *R*S)-A1]

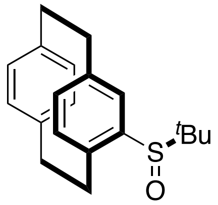

Chiral SFC conditions: Chiralcel-OJ-3, 2.5 mL/min, 254 nm, CO_2/MeOH, 95/5)

(*S*P, *R*S)-A1 and (*R*P, *R*S)-A1

#	Time/Min	Area%
**1**	2.668	50.49%
**2**	6.502	49.51%

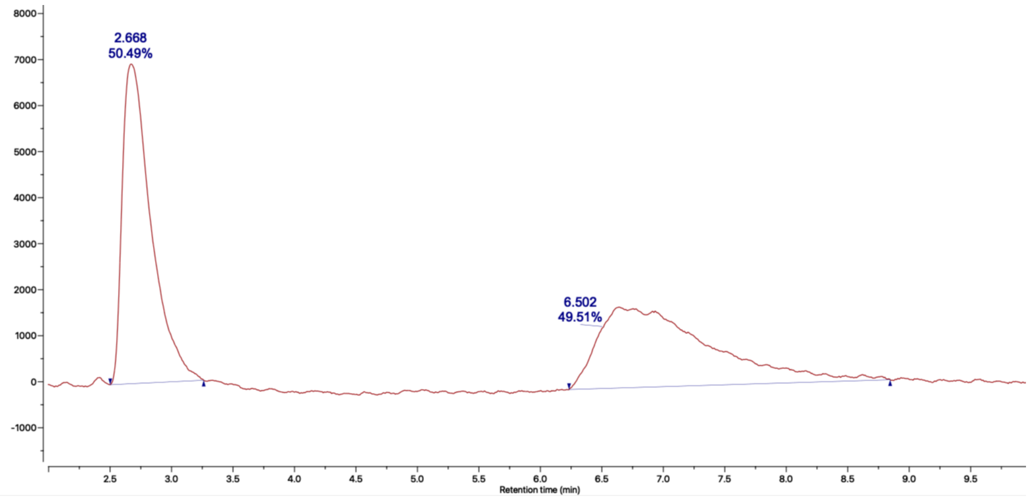

Figure S2. SFC spectrum of (*S*P, *R*S)-A1 and (*R*P, *R*S)-A1

(*S*P, *R*S)-A1, 99% *ee*

#	Time/Min	Area%
**1**	2.307	99.28%
**2**	7.163	0.72%

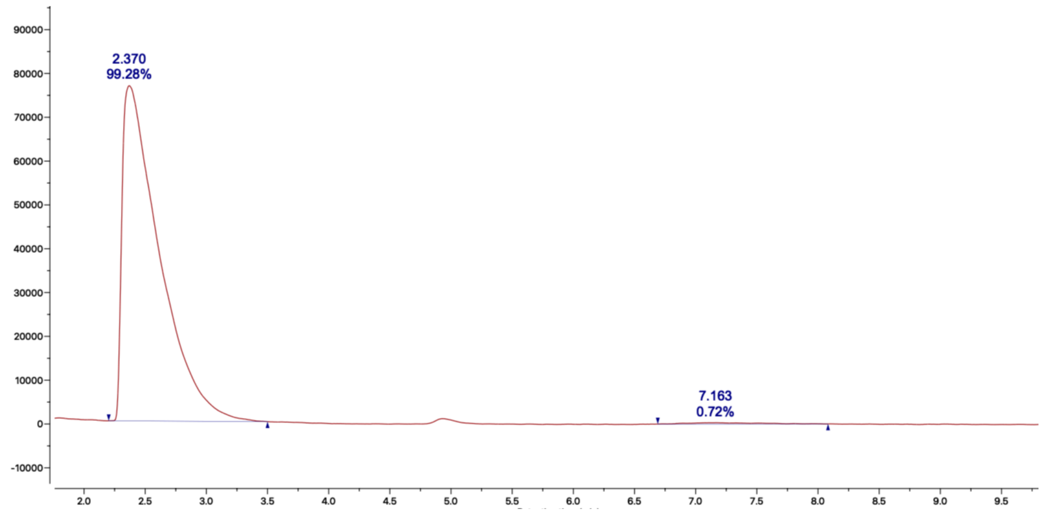

Figure S3. SFC spectrum of (S_P, R_S)-A1

(S_P, S_S)-4-*p*-phenylethylsulfinyl[2.2]paracyclophane [(S_p,S_S)-A2] and (S_P, R_S)-4-*p*-phenylethylsulfinyl[2.2]paracyclophane [(S_p,R_S)-A2]

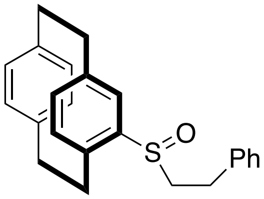

Chiral SFC conditions: Chiralcel-OJ-3, 2.5 mL/min, 254 nm, CO_2/MeOH, 95/5)

Racemic A2

#	Time/Min	Area%
**1**	4.085	35.00%
**2**	5.020	34.68%
**3**	7.901	15.01%
**4**	8.406	15.31%

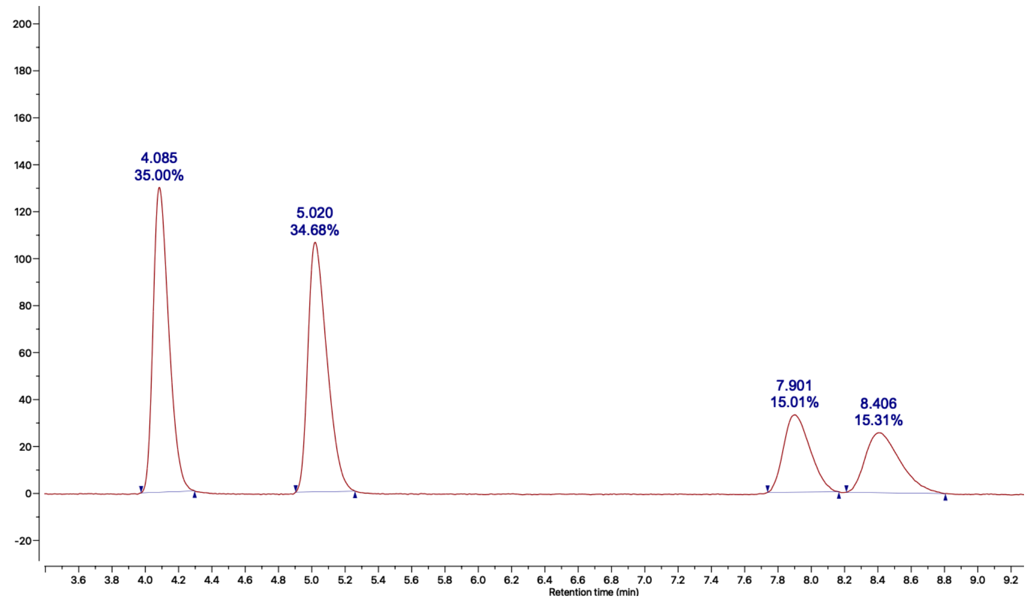

Figure S4. SFC spectrum of racemic A2

(S_p,S_S)-A2 and (S_p,R_S)-A2, >99% *ee*

#	Time/Min	Area%
**1**	4.053	64.88%
**2**	8.309	35.12%

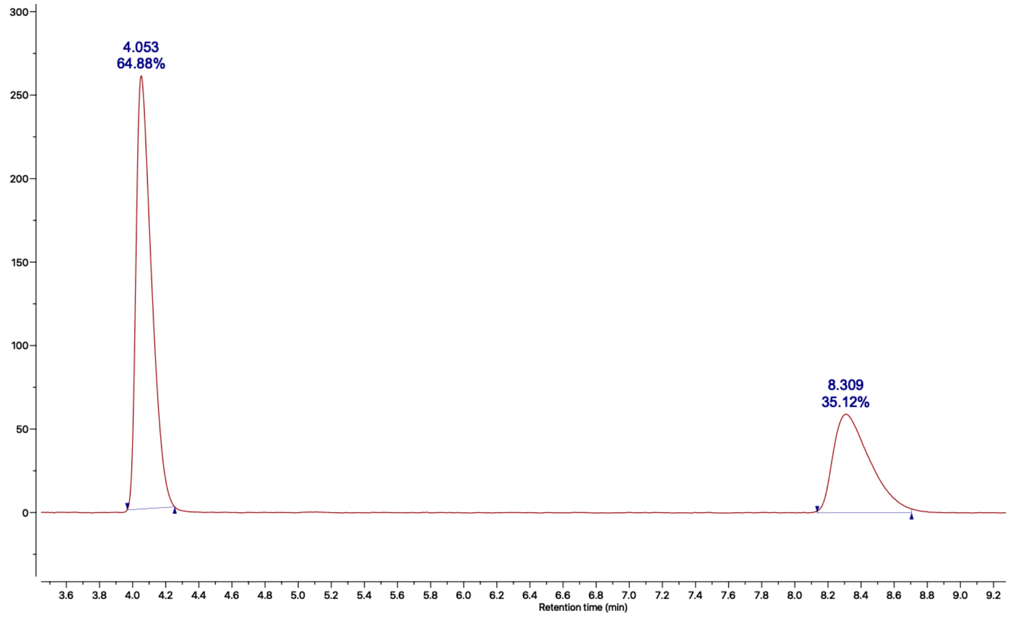

Figure S5. SFC spectrum of (S_p,S_S)-A2 and (S_p,R_S)-A2

(2*S*,3*S*)-2-cyclopropyl-1-(4-methoxyphenyl)-3-phenylaziridine (3a)

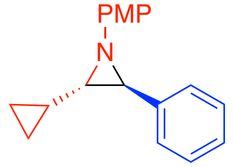

Chiral SFC conditions: Chiralcel-OJ-3, 2.5 mL/min, 254 nm, CO_2/MeOH, 95/5)

Racemic 3a

#	Time/Min	Area%
**1**	2.379	49.32%
**2**	3.045	50.68%

(2*S*,3*S*) 3a, 97% *ee*

#	Time/Min	Area%
**1**	2.426	1.50%
**2**	3.057	98.50%

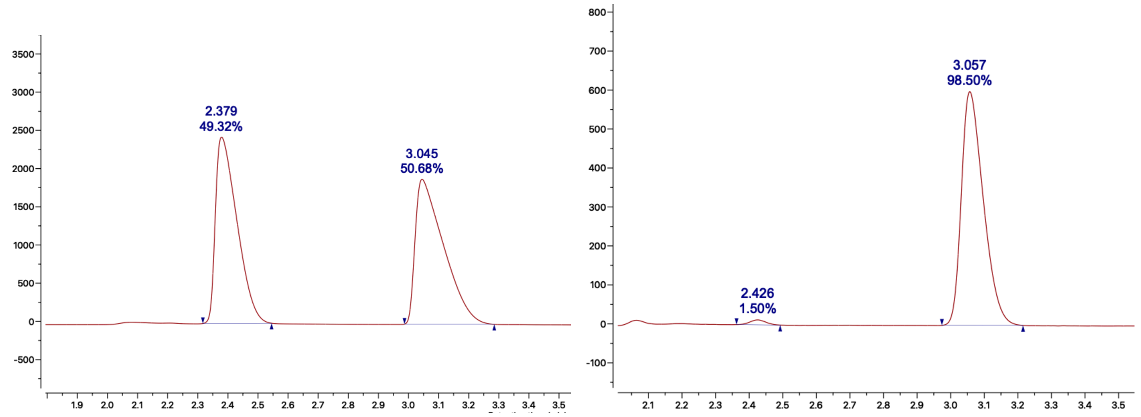

Figure S6. SFC spectrum of racemic 3a and (2*S*,3*S*) 3a

(2*S*,3*S*)-2-cyclopropyl-1-(4-methoxyphenyl)-3-(*p*-tolyl)aziridine (3b)

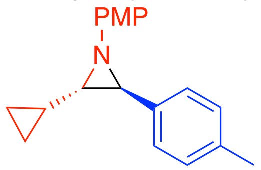

Chiral SFC conditions: Chiralcel-OJ-3, 2.5 mL/min, 254 nm, CO_2/MeOH, 95/5

Racemic 3b

#	Time/Min	Area%
**1**	2.384	49.86%
**2**	2.859	50.14%

(2*S*,3*S*) 3b, 97% *ee*

#	Time/Min	Area%
**1**	2.371	1.67%
**2**	2.806	98.33%

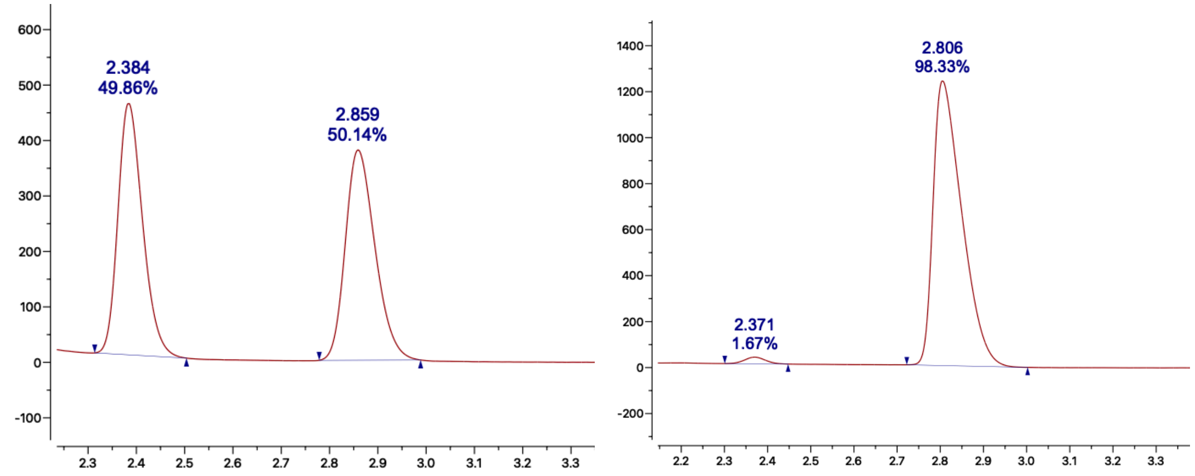

Figure S7. SFC spectrum of racemic 3b and (2*S*,3*S*) 3b

(2*S*,3*S*)-2-(4-(*tert*-butyl)phenyl)-3-cyclopropyl-1-(4-methoxyphenyl)aziridine (3c)

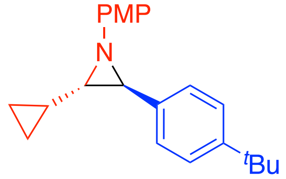

Chiral SFC conditions: Chiralcel-IA-3, 2.5 mL/min, 254 nm, CO_2/MeOH, 97.5/2.5

Racemic 3c

#	Time/Min	Area%
**1**	2.668	51.45%
**2**	6.859	48.55%

(2*S*,3*S*) 3c, 98% *ee*

#	Time/Min	Area%
**1**	2.370	98.95%
**2**	7.168	1.05%

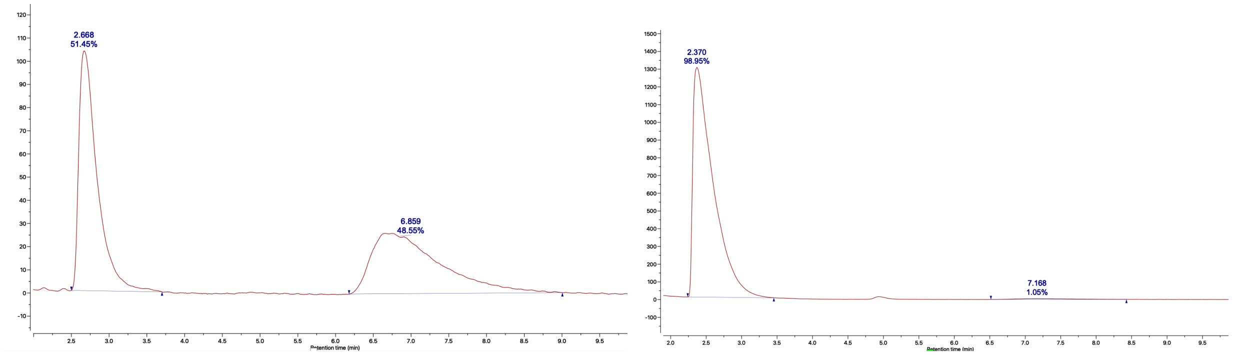

Figure S8. SFC spectrum of racemic 3c and (2*S*,3*S*) 3c

(2*S*,3*S*)-2-cyclopropyl-1-(4-methoxyphenyl)-3-(4-(methylthio)phenyl)aziridine (3d)

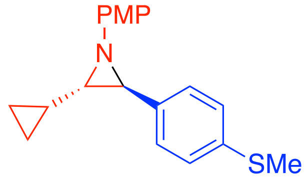

Chiral SFC conditions: Chiralcel-OJ-3, 2.5 mL/min, 254 nm, CO_2/MeOH, 90/10

Racemic 3d

#	Time/Min	Area%
**1**	3.182	49.75%
**2**	3.607	50.25%

(2*S*,3*S*) 3d, 95% *ee*

#	Time/Min	Area%
**1**	3.134	2.71%
**2**	3.406	97.29%

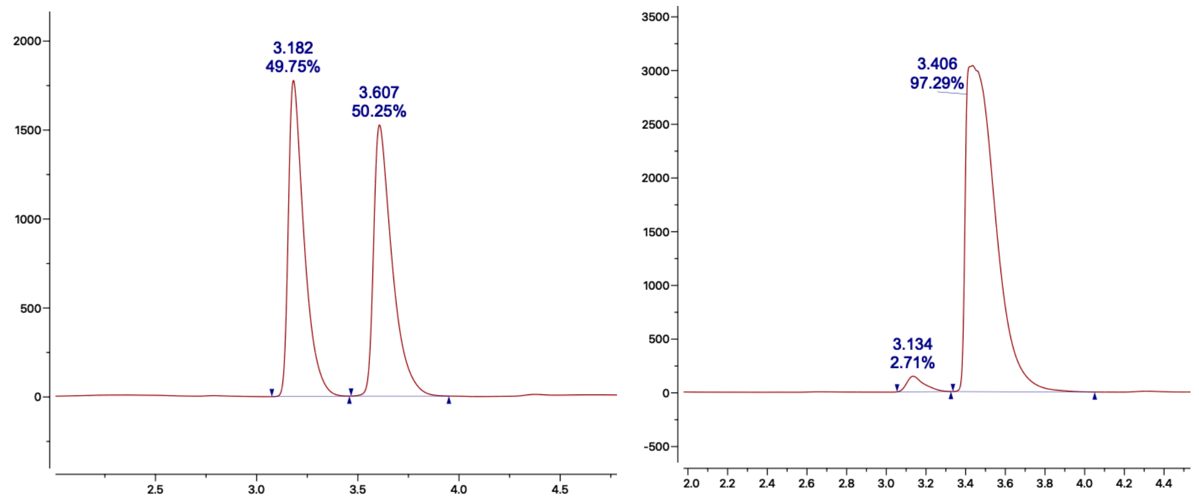

Figure S9. SFC spectrum of racemic 3d and (2*S*,3*S*) 3d

(2*S*,3*S*)-2-cyclopropyl-1-(4-methoxyphenyl)-3-(4-phenethylphenyl)aziridine (3e)

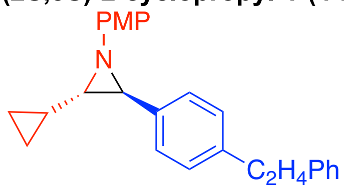

Chiral SFC conditions: Chiralcel-IA-3, 2.5 mL/min, 254 nm, CO_2/MeOH, 90/10

Racemic 3e

#	Time/Min	Area%
**1**	2.620	50.12%
**2**	6.744	49.88%

(2*S*,3*S*) 3e, 95% *ee*

#	Time/Min	Area%
**1**	2.698	97.58%
**2**	7.412	2.42%

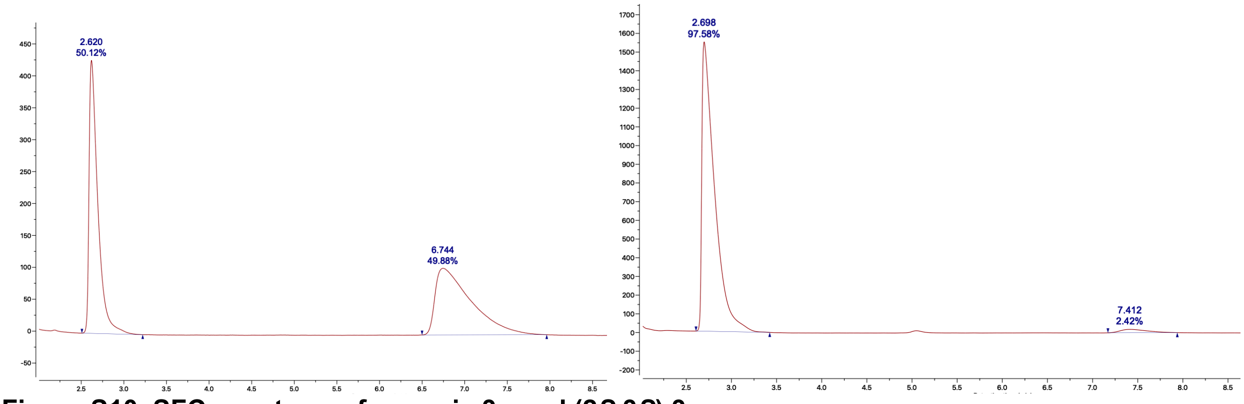

Figure S10. SFC spectrum of racemic 3e and (2*S*,3*S*) 3e

(2*S*,3*S*)-2-cyclopropyl-3-mesityl-1-(4-methoxyphenyl)aziridine (3f)

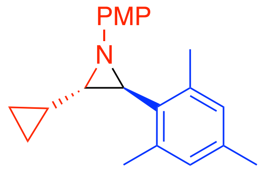

Chiral SFC conditions: Chiralcel-IF-3, 2.5 mL/min, 254 nm, CO_2/MeOH, 95/5

Racemic 3f

#	Time/Min	Area%
**1**	2.768	49.73%
**2**	3.297	50.27%

(2*S*,3*S*) 3f, 93% *ee*

#	Time/Min	Area%
**1**	2.523	96.33%
**2**	3.582	3.67%

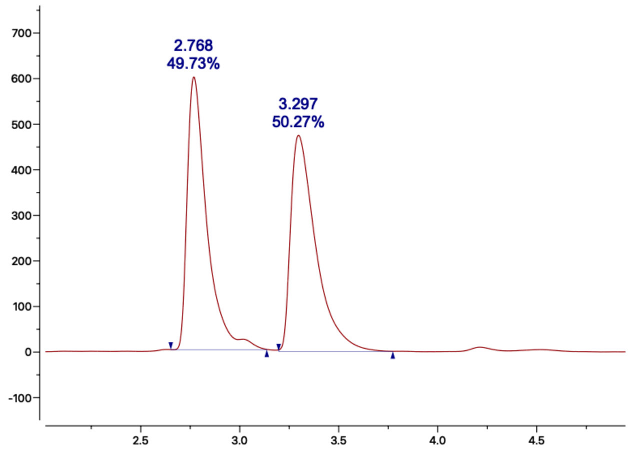

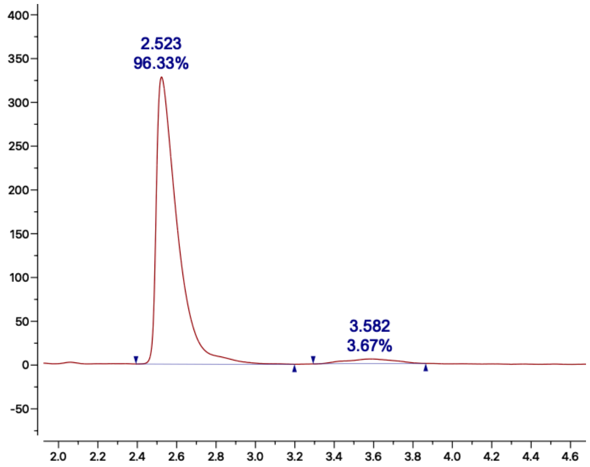

Figure S11. SFC spectrum of racemic 3f and (2*S*,3*S*) 3f

(2*S*,3*S*)-2-cyclopropyl-1-(4-methoxyphenyl)-3-(4-phenoxyphenyl)aziridine (3g)

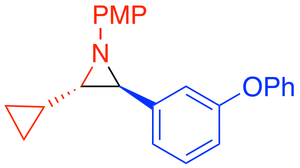

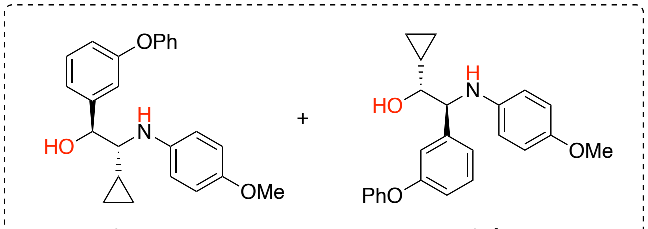

Chiral SFC conditions: Chiralcel-IC-3, 2.5 mL/min, 254 nm, CO_2/MeOH, 90/10

Racemic 3ga and 3gb

#	Time/Min	Area%
**1**	3.930	26.15%
**2**	4.540	25.07%
**3**	5.513	23.41%
**4**	6.357	25.37%

(1*S*,2*R*) 3ga and (1*R*,2*S*) 3gb, 97% *ee*

#	Time/Min	Area%
**1**	4.038	46.25%
**2**	4.735	1.85%
**3**	5.885	1.81%
**4**	6.496	50.09%

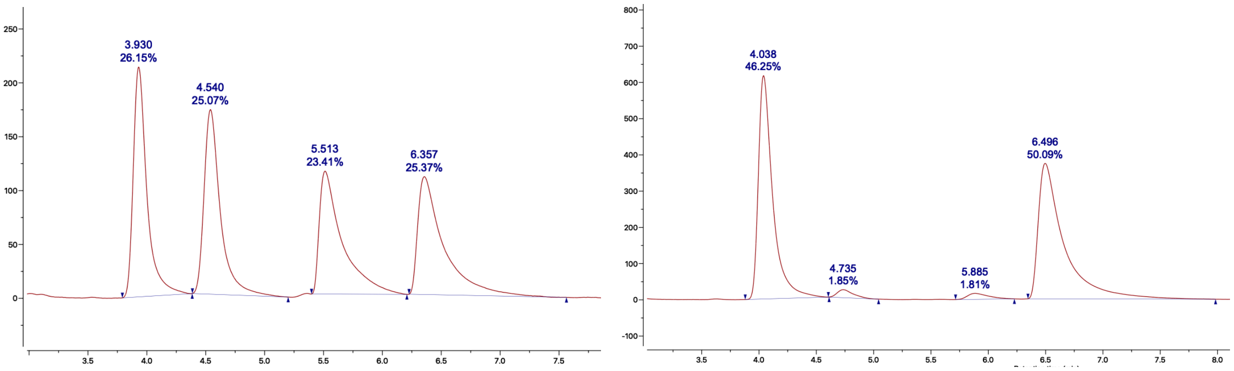

Figure S12. SFC spectrum of racemic 3ga and 3gb, and (1*S*,2*R*) 3ga and (1*R*,2*S*) 3gb

(2*S*,3*S*)-2-cyclopropyl-1-(4-methoxyphenyl)-3-(3-(trifluoromethyl)phenyl)aziridine (3h)

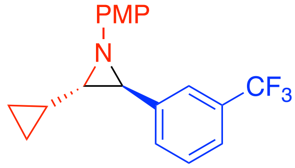

Chiral SFC conditions: Chiralcel-OJ-3, 2.5 mL/min, 254 nm, CO_2/MeOH, 97.5/2.5

Racemic 3h

#	Time/Min	Area%
**1**	1.282	49.82%
**2**	1.646	50.18%

(2*S*,3*S*) 3h, 84% *ee*

#	Time/Min	Area%
**1**	1.287	7.82%
**2**	1.633	92.18%

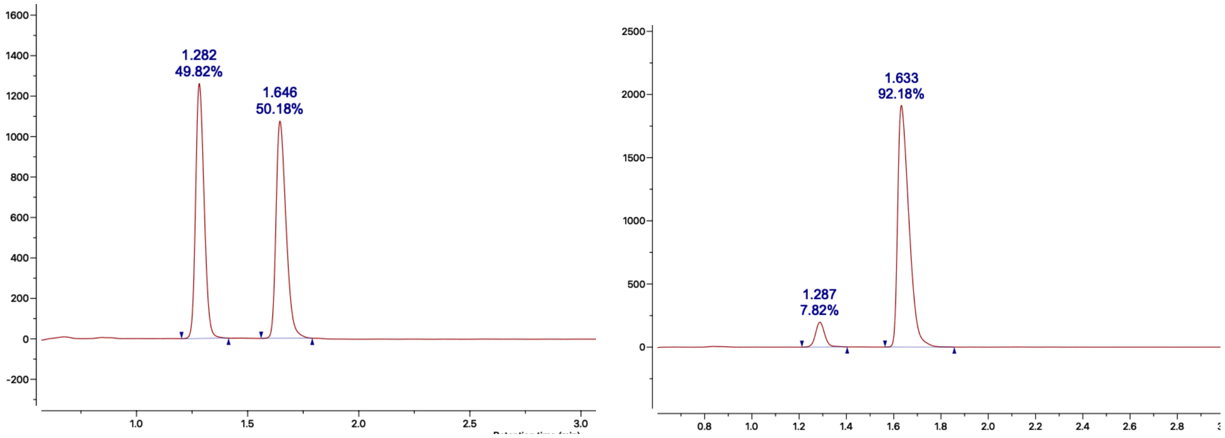

Figure S13. SFC spectrum of racemic 3h and (2*S*,3*S*) 3h

(2*S*,3*S*)-2-cyclopropyl-1-(4-methoxyphenyl)-3-(4-(trifluoromethoxy)phenyl)aziridine (3i)

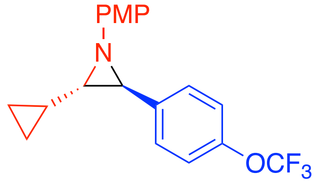

Chiral SFC conditions: Chiralcel-OJ-3, 2.5 mL/min, 254 nm, CO_2/MeOH, 95/5

Racemic 3i

#	Time/Min	Area%

(2*S*,3*S*) 3i, 97% *ee*

#	Time/Min	Area%

**1**	2.167	49.63%
**2**	2.475	50.37%

**1**	2.154	98.32%
**2**	2.522	1.68%

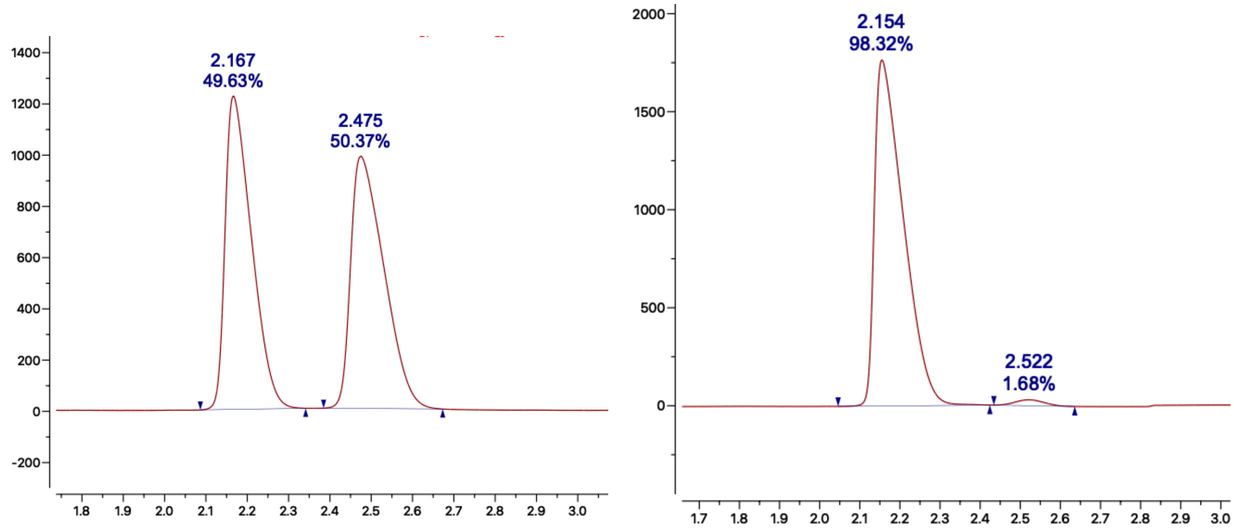

Figure S14. SFC spectrum of racemic 3i and (2*S*,3*S*) 3i

(2*S*,3*S*)-2-(3-bromophenyl)-3-cyclopropyl-1-(4-methoxyphenyl)aziridine (3j)

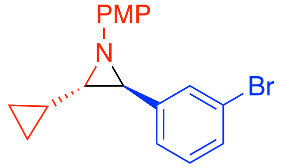

Chiral SFC conditions: Chiralcel-OJ-3, 2.5 mL/min, 254 nm, CO_2/MeOH, 95/5

Racemic 3j

#	Time/Min	Area%
**1**	3.029	50.22%
**2**	3.270	49.78%

(2*S*,3*S*) 3j, 90% *ee*

#	Time/Min	Area%
**1**	2.998	94.75%
**2**	3.301	5.25%

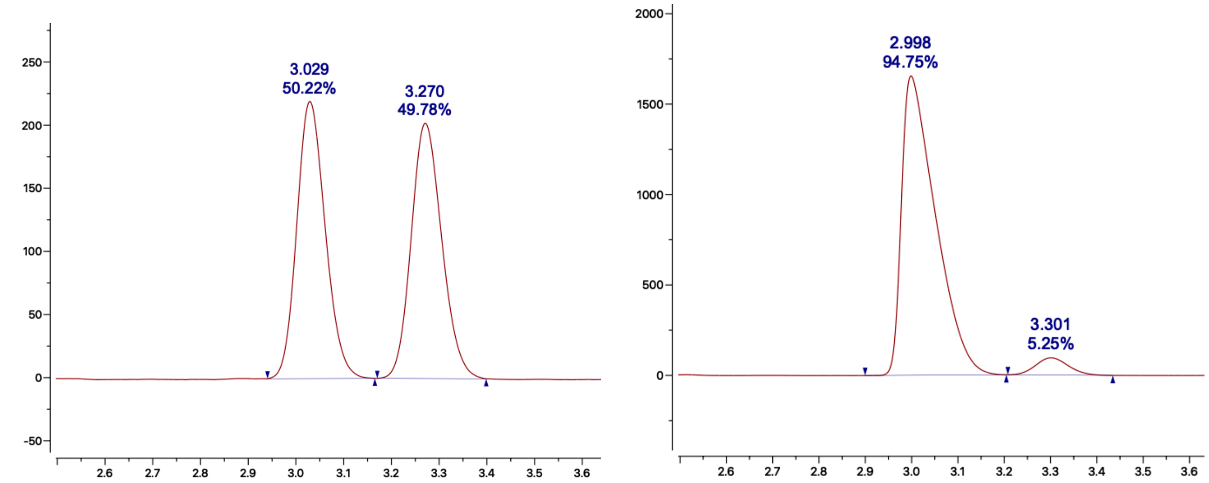

Figure S15. SFC spectrum of racemic 3j and (2*S*,3*S*) 3j

(2*S*,3*S*)-2-(2-bromophenyl)-3-cyclopropyl-1-(4-methoxyphenyl)aziridine (3k)

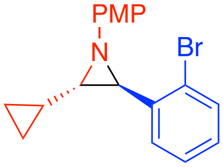

Chiral SFC conditions: Chiralcel-IA-3, 2.5 mL/min, 254 nm, CO_2/MeOH, 95/5

Racemic 3k

#	Time/Min	Area%
**1**	2.267	50.54%
**2**	3.060	49.46%

(2*S*,3*S*) 3k, 73% *ee*

#	Time/Min	Area%
**1**	2.183	86.28%
**2**	3.203	13.72%

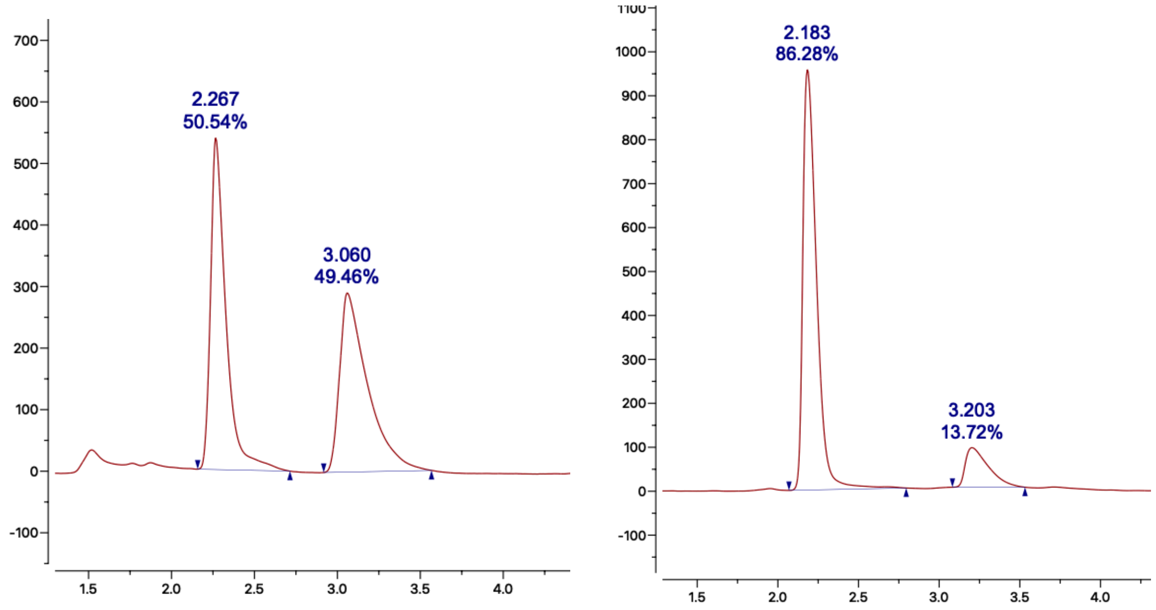

Figure S16. SFC spectrum of racemic 3k and (2*S*,3*S*) 3k

(2*S*,3*S*)-2-cyclopropyl-3-(4-fluorophenyl)-1-(4-methoxyphenyl)aziridine (3l)

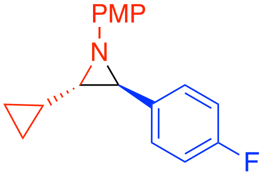

Chiral SFC conditions: Chiralcel-OJ-3, 2.5 mL/min, 254 nm, CO_2/MeOH, 99/1

Racemic 3l

#	Time/Min	Area%
**1**	5.314	50.30%
**2**	6.494	49.70%

(2*S*,3*S*) 3l, >99% *ee*

#	Time/Min	Area%
**1**	5.213	100.00%

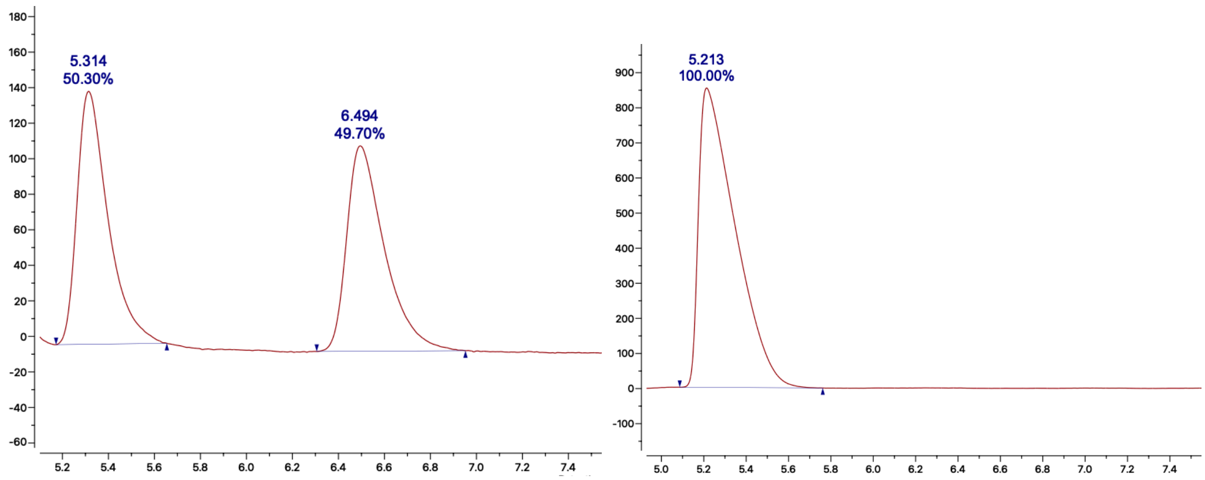

Figure S17. SFC spectrum of racemic 3l and (2*S*,3*S*) 3l

(2*S*,3*S*)-2-cyclopropyl-3-(2-fluorophenyl)-1-(4-methoxyphenyl)aziridine (3m)

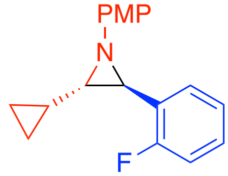

Chiral SFC conditions: Chiralcel-OJ-3, 2.5 mL/min, 254 nm, CO_2/MeOH, 97.5/2.5

Racemic 3m

#	Time/Min	Area%
**1**	2.025	49.22%
**2**	2.438	50.78%

(2*S*,3*S*) 3m, 95% *ee*

#	Time/Min	Area%
**1**	1.900	2.52%
**2**	2.236	97.48%

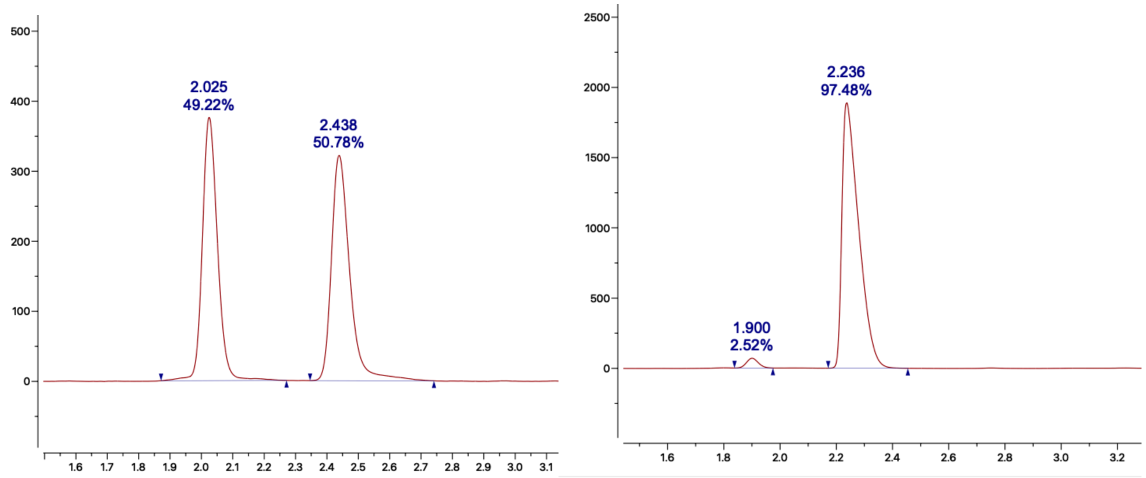

Figure S18. SFC spectrum of racemic 3m and (2*S*,3*S*) 3m

(2*S*,3*S*)-2-(4-chlorophenyl)-3-cyclopropyl-1-(4-methoxyphenyl)aziridine (3n)

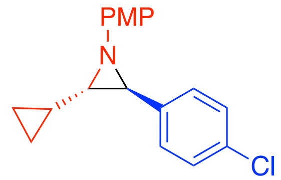

Chiral SFC conditions: Chiralcel-OJ-3, 2.5 mL/min, 254 nm, CO_2/MeOH, 95/5

Racemic 3n

#	Time/Min	Area%
**1**	3.055	50.55%
**2**	3.350	49.45%

(2*S*,3*S*) 3n, 95% *ee*

#	Time/Min	Area%
**1**	3.045	97.72%
**2**	3.392	2.28%

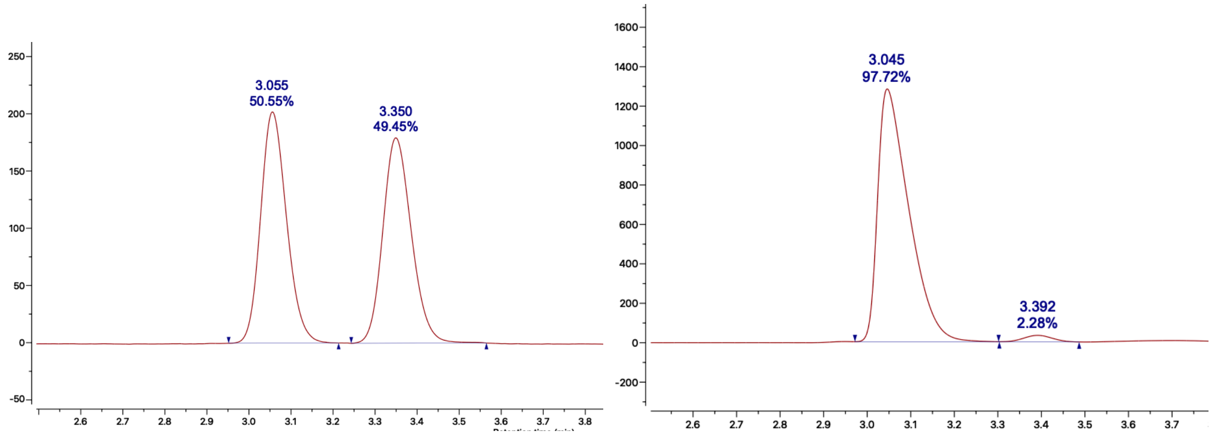

Figure S19. SFC spectrum of racemic 3n and (2*S*,3*S*) 3n

(2*S*,3*S*)-2-cyclopropyl-1-(4-methoxyphenyl)-3-(naphthalen-1-yl)aziridine (3o)

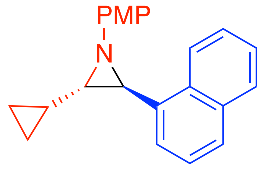

Chiral SFC conditions: Chiralcel-OJ-3, 2.5 mL/min, 254 nm, CO_2/MeOH, 95/5

Racemic 3o

#	Time/Min	Area%
**1**	3.515	49.89%
**2**	5.431	50.11%

(2*S*,3*S*) 3o, 92% *ee*

#	Time/Min	Area%
**1**	3.522	3.78%
**2**	5.410	96.22%

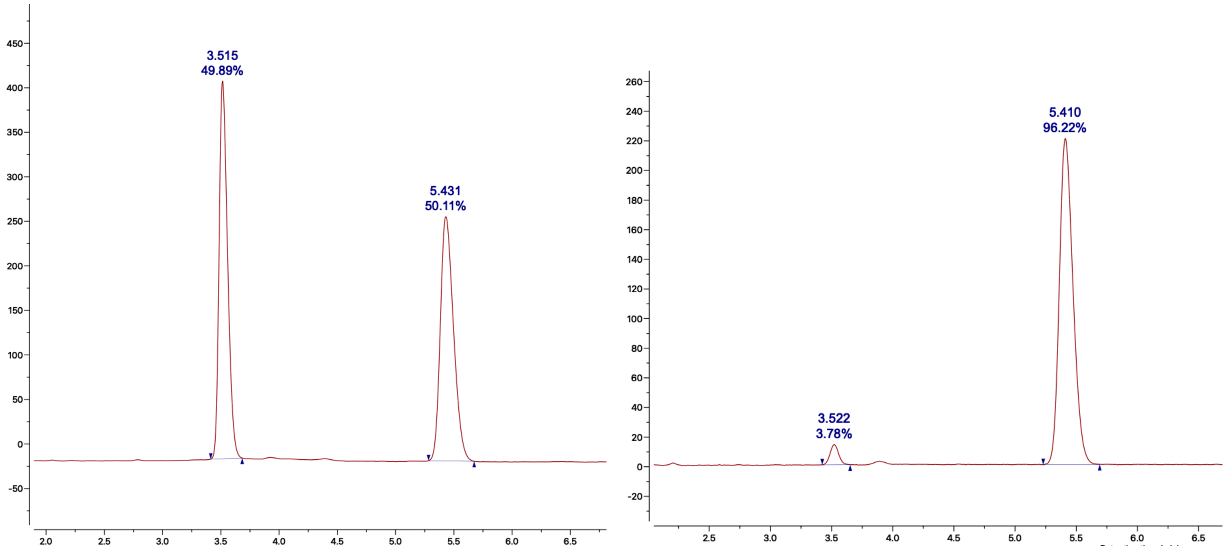

Figure S20. SFC spectrum of racemic 3o and (2*S*,3*S*) 3o

(2*S*,3*S*)-2-cyclopropyl-1-(4-methoxyphenyl)-3-(4-vinylphenyl)aziridine (3p)

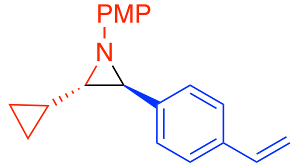

Chiral SFC conditions: Chiralcel-OJ-3, 2.5 mL/min, 254 nm, CO_2/MeOH, 97.5/2.5

Racemic 3p

#	Time/Min	Area%
**1**	5.542	50.41%
**2**	6.410	49.59%

(2*S*,3*S*) 3p, 96% *ee*

#	Time/Min	Area%
**1**	5.613	2.25%
**2**	6.445	97.75%

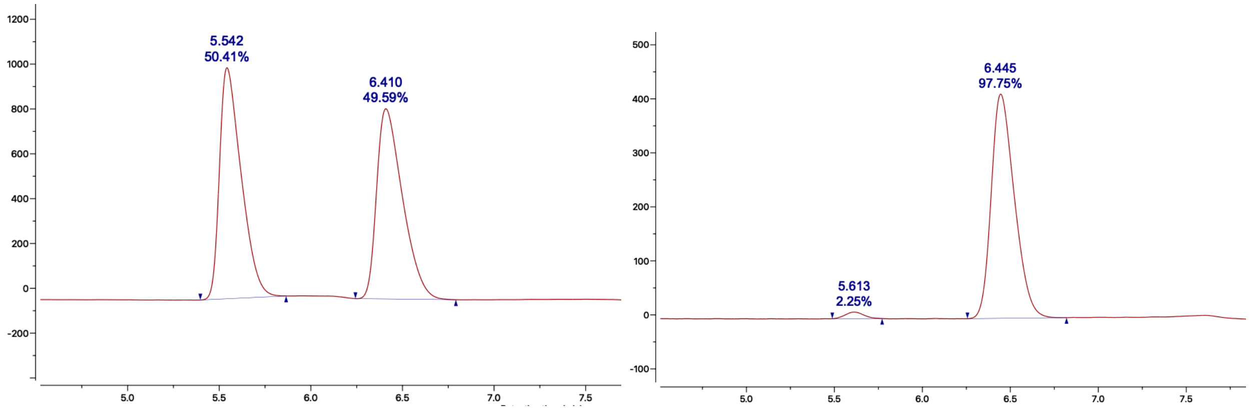

Figure S21. SFC spectrum of racemic 3p and (2*S*,3*S*) 3p

2-(3-((2*S*,3*S*)-3-cyclopropyl-1-(4-methoxyphenyl)aziridin-2-yl)phenyl)pyrimidine (3q)

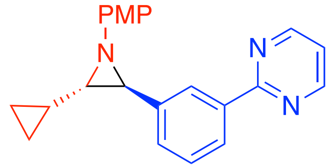

Chiral SFC conditions: Chiralcel-OJ-3, 2.5 mL/min, 254 nm, CO_2/MeOH, 95/5

Racemic 3q

#	Time/Min	Area%
**1**	9.917	48.33%
**2**	11.732	51.67%

(2*S*,3*S*) 3q, 73% *ee*

#	Time/Min	Area%
**1**	10.288	13.53%
**2**	12.048	86.47%

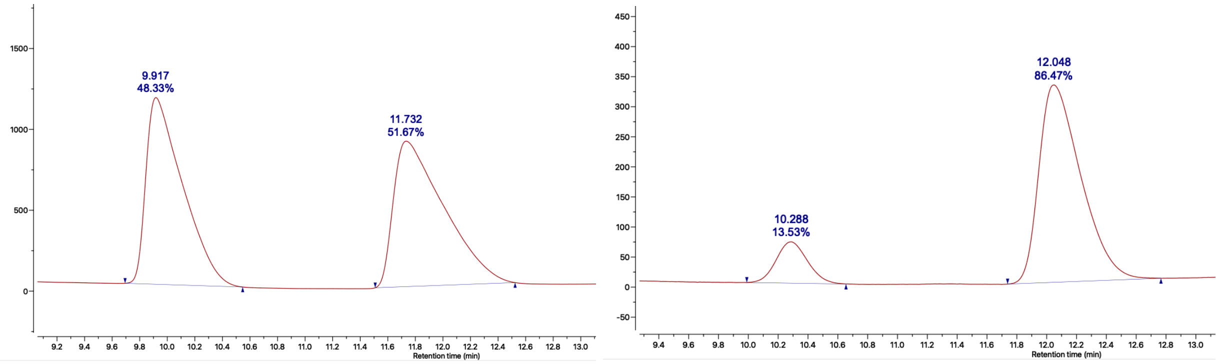

Figure S22. SFC spectrum of racemic 3q and (2*S*,3*S*) 3q

(2*S*,3*S*)-2-cyclopropyl-1-(4-methoxyphenyl)-3-vinylaziridine (3r)

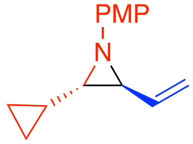

Chiral SFC conditions: Chiralcel-OJ-3, 2.5 mL/min, 254 nm, CO_2/MeOH, 97.5/2.5

Racemic 3r

#	Time/Min	Area%
**1**	1.374	50.34%
**2**	1.596	49.66%

(2*S*,3*S*) 3r, 89% *ee*

#	Time/Min	Area%
**1**	1.434	94.61%
**2**	1.672	5.39%

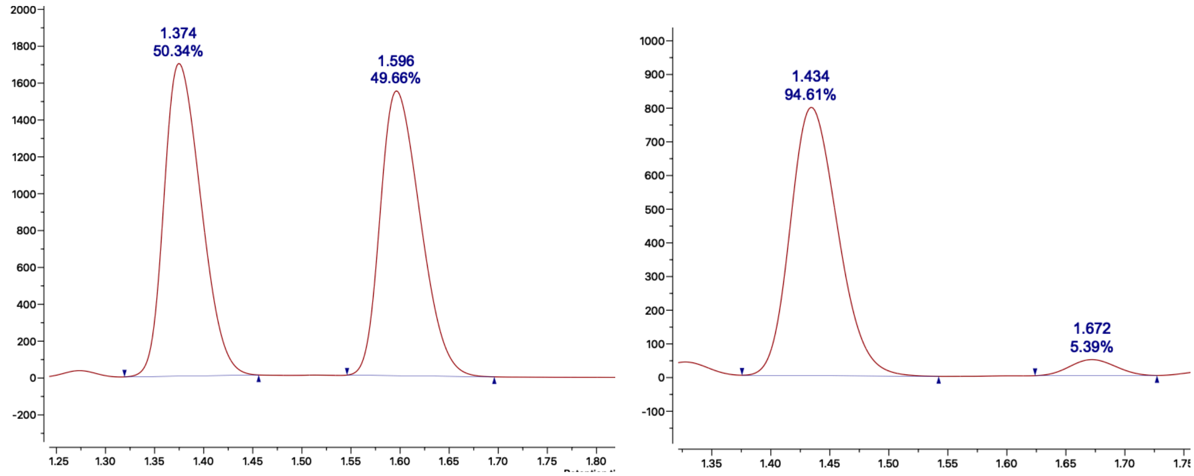

Figure S23. SFC spectrum of racemic 3r and (2*S*,3*S*) 3r

NMR spectra

400 MHz ^{1}H NMR spectra of **1a** in $CDCl_3$

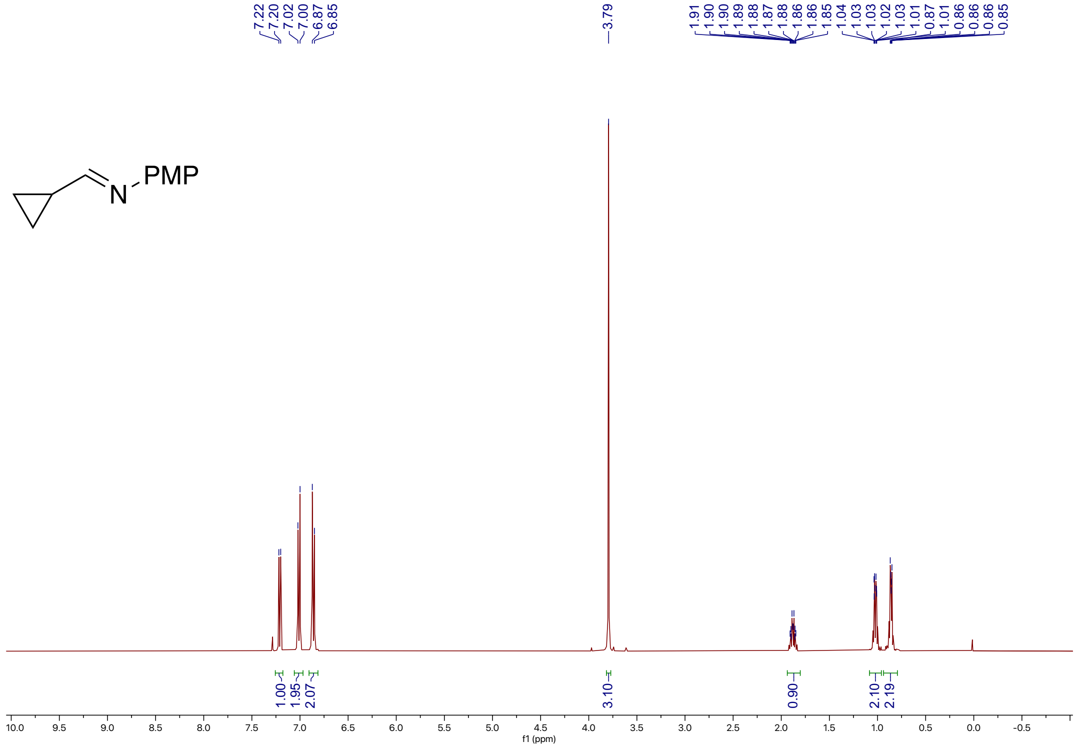

101 MHz $^{13}C\{^{1}H\}$ NMR spectrum of **1a** in $CDCl_3$

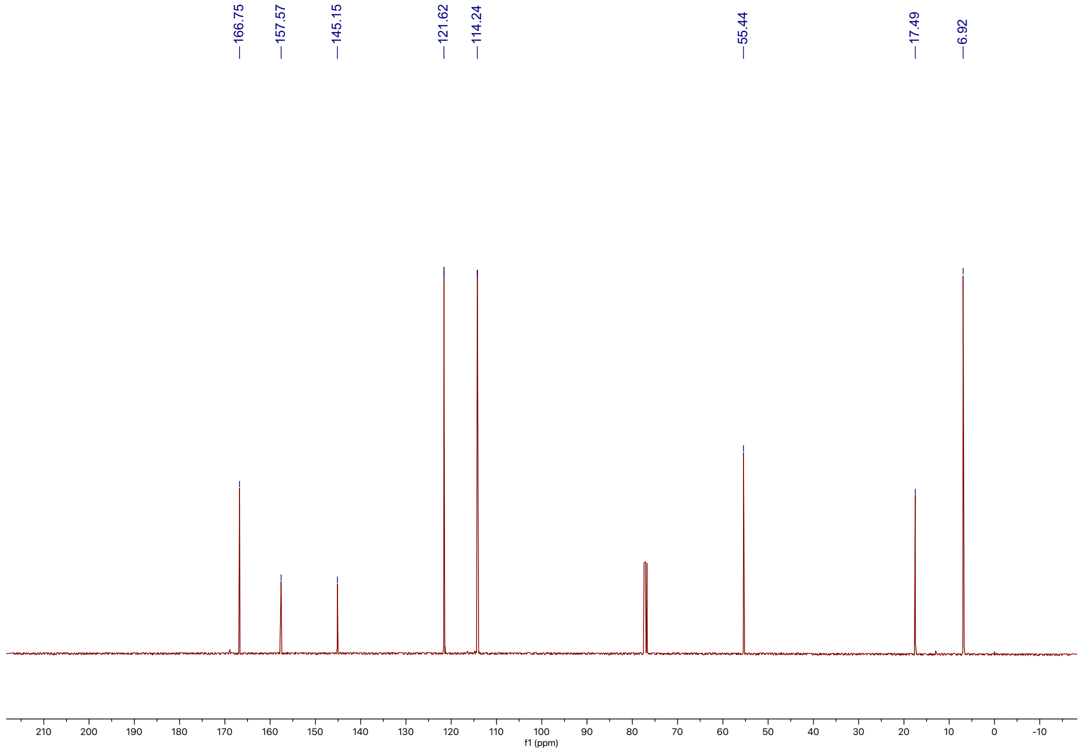

400 MHz ^{1}H NMR spectra of **S2** in $CDCl_3$

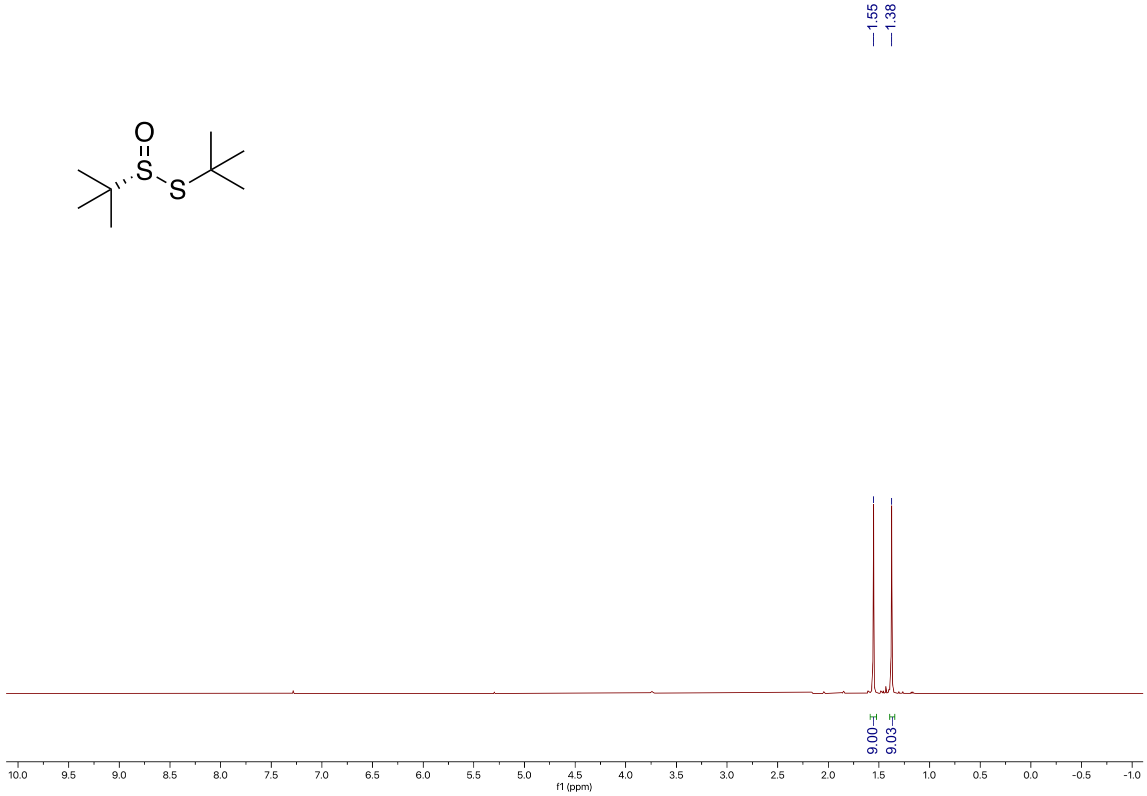

101 MHz $^{13}C\{^1H\}$ NMR spectrum of **S2** in $CDCl_3$

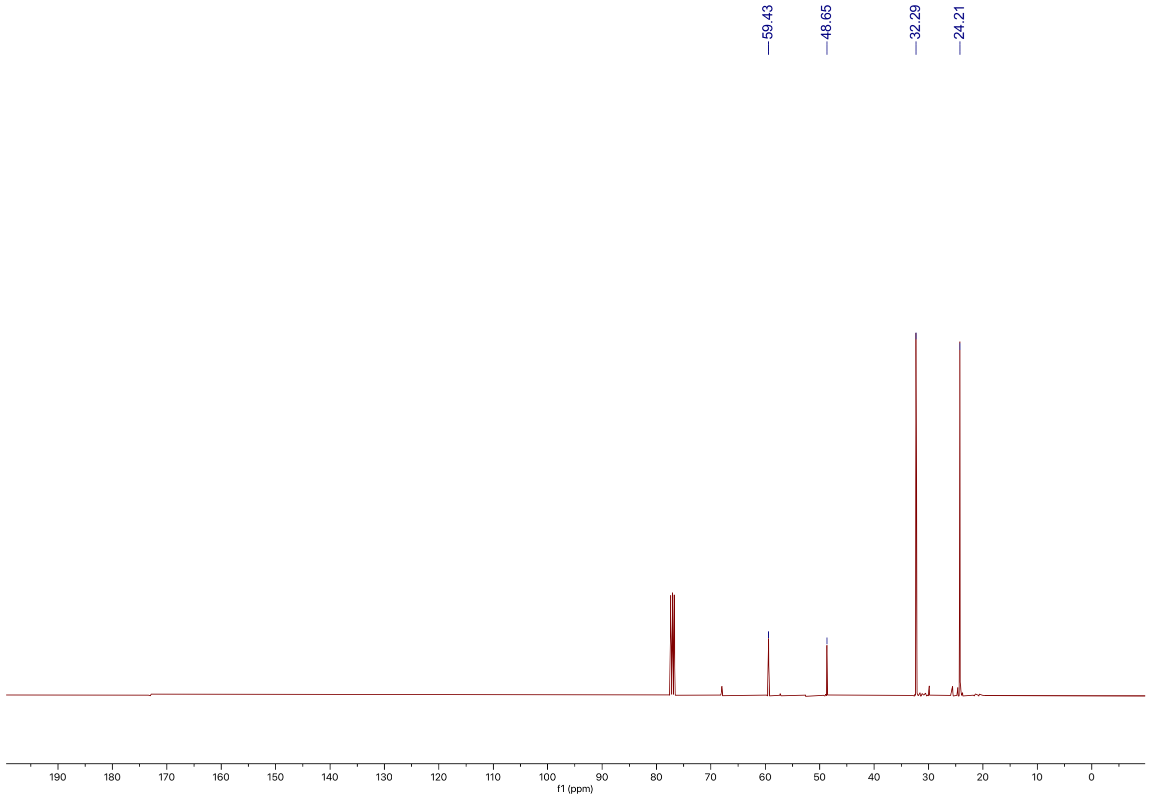

400 MHz ^{1}H NMR spectra of **S5** in $CDCl_3$

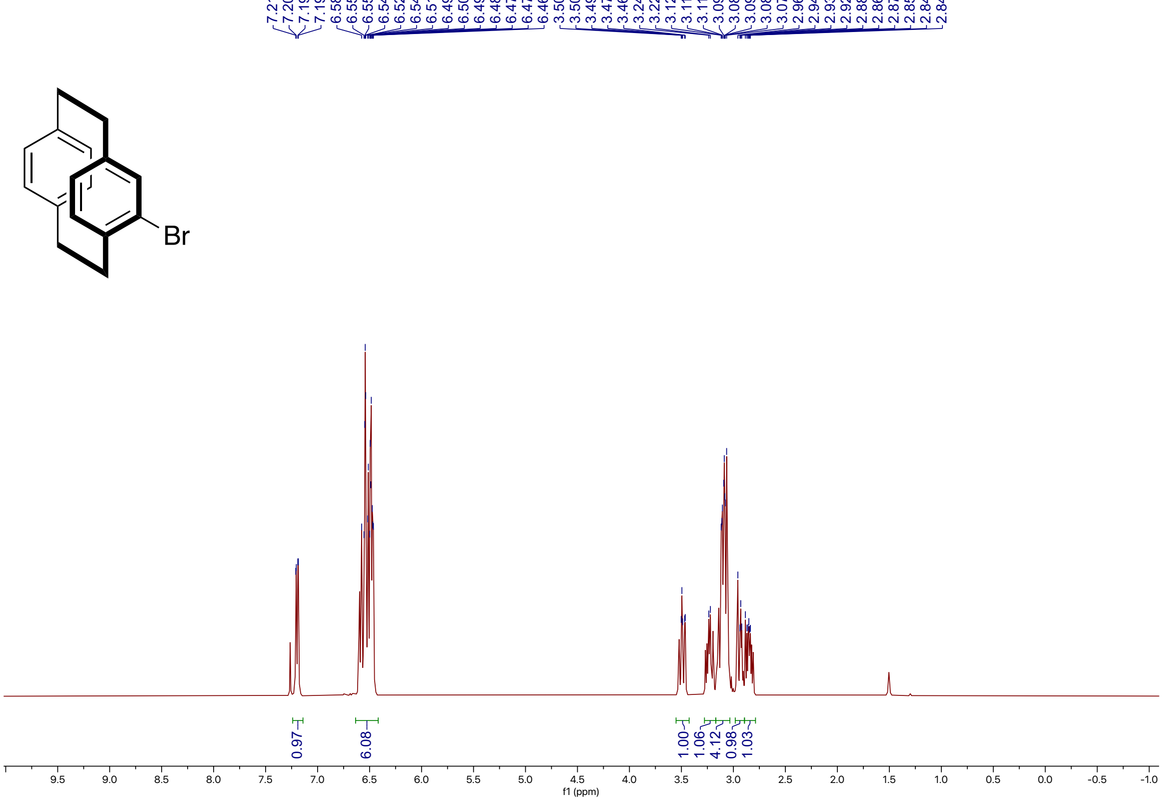

101 MHz $^{13}C\{^1H\}$ NMR spectrum of **S5** in $CDCl_3$

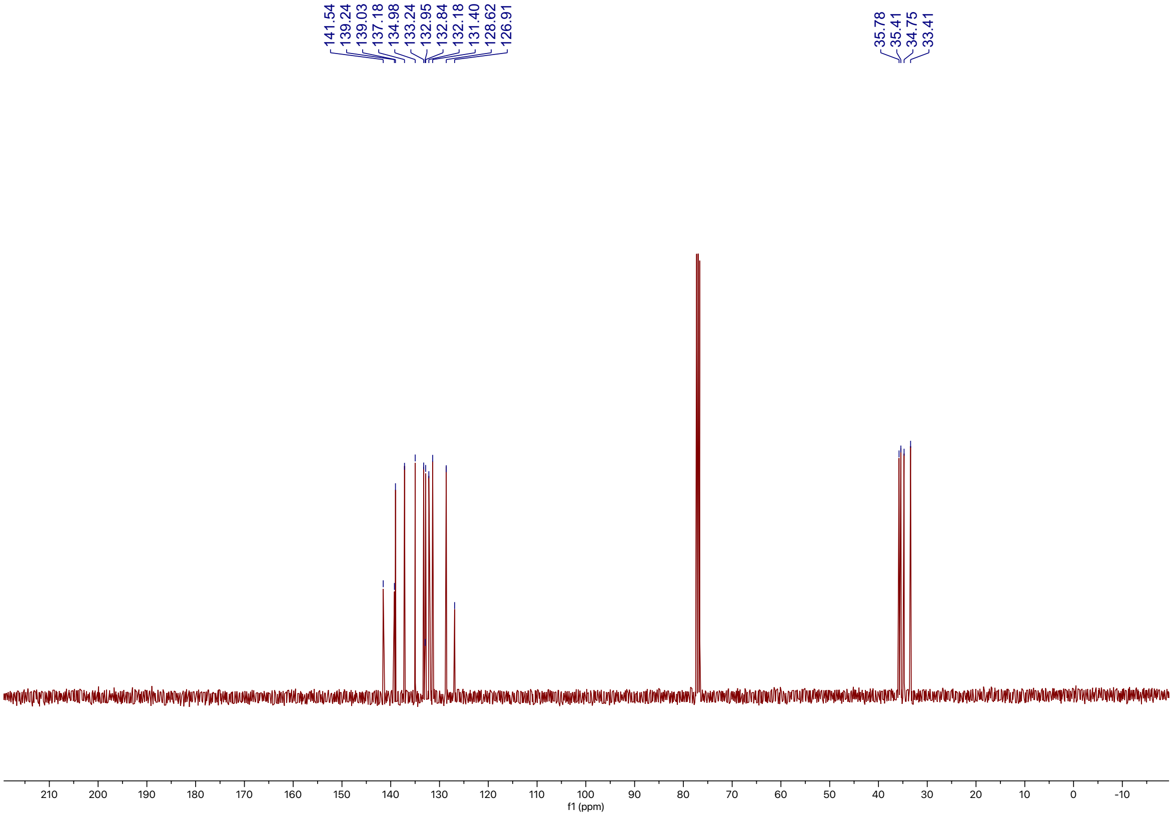

400 MHz ^{1}H NMR spectra of **[(*S*$_P$, *R*$_S$)-A1]** in $CDCl_3$

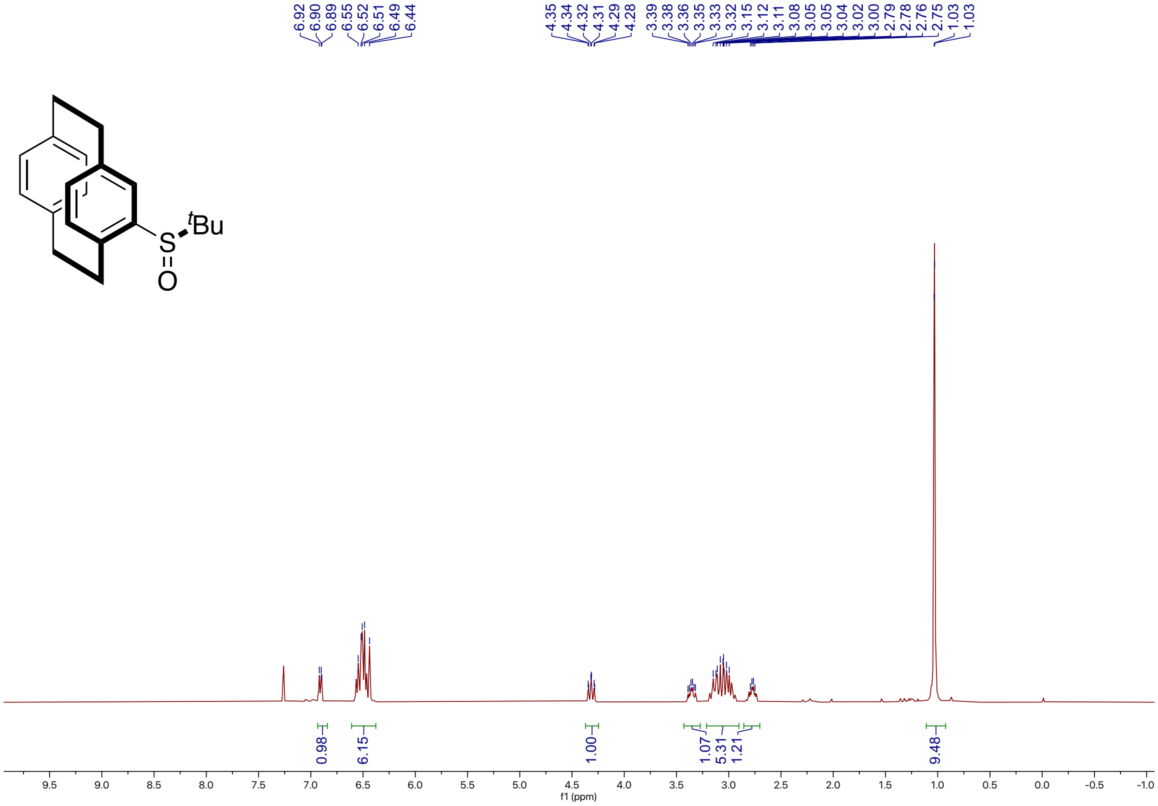

101 MHz $^{13}C\{^1H\}$ NMR spectrum of **[(*S*$_P$, *R*$_S$)-A1]** in $CDCl_3$

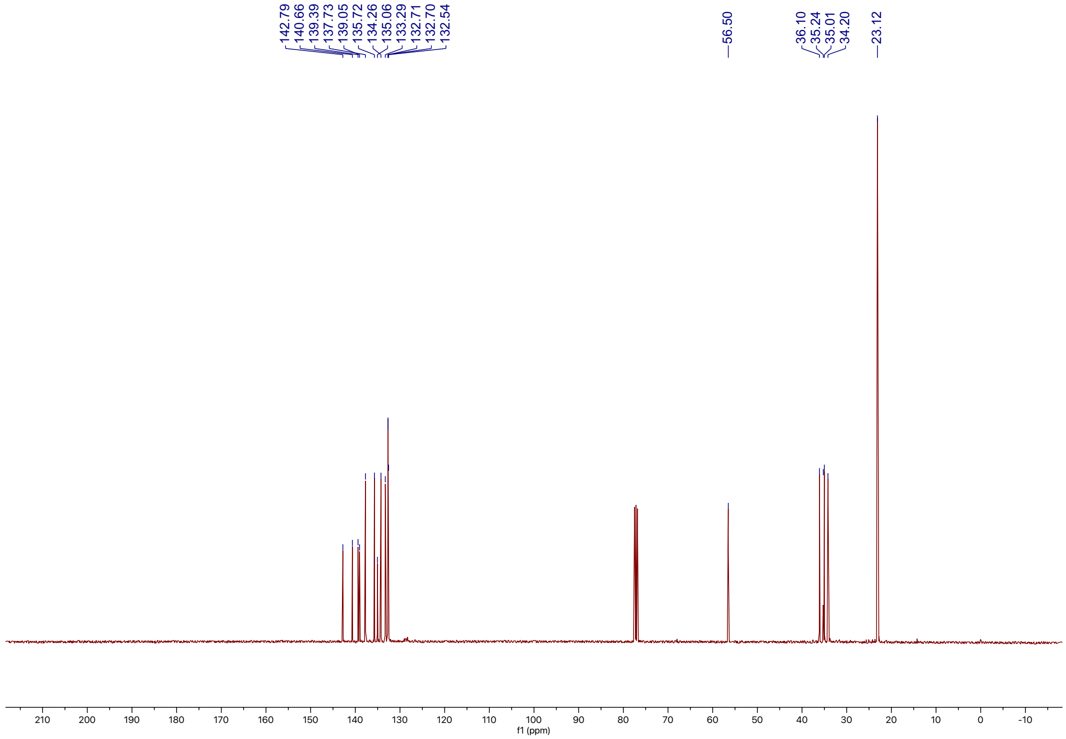

400 MHz ^{1}H NMR spectra of **[(*R*$_P$, *R*$_S$)-A1]** in $CDCl_3$

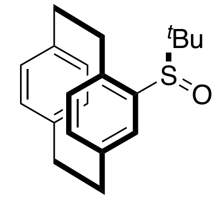

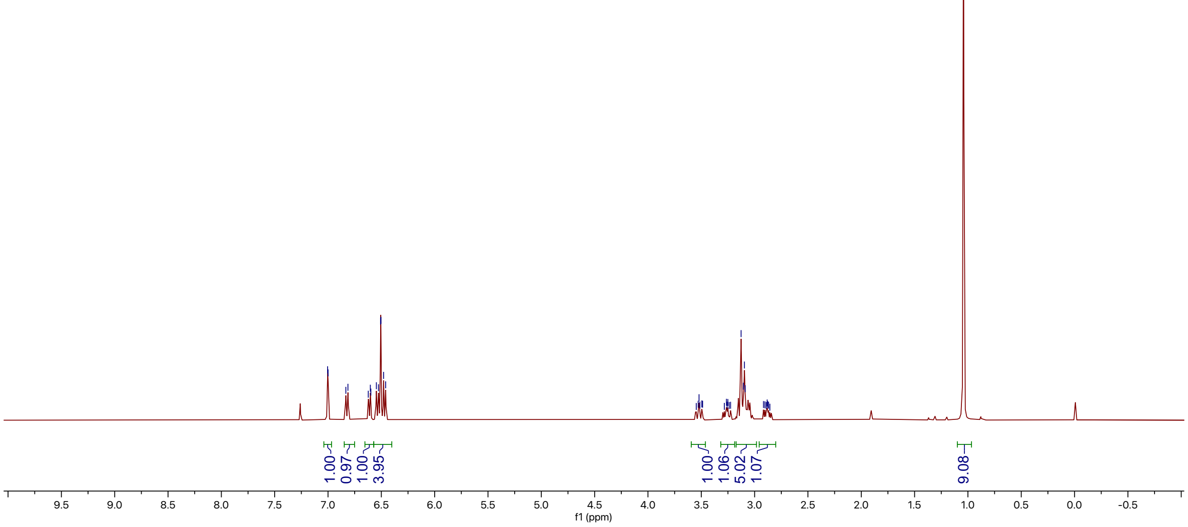

101 MHz ^{13}C{^{1}H} NMR spectrum of **[(*R*$_P$, *R*$_S$)-A1]** in $CDCl_3$

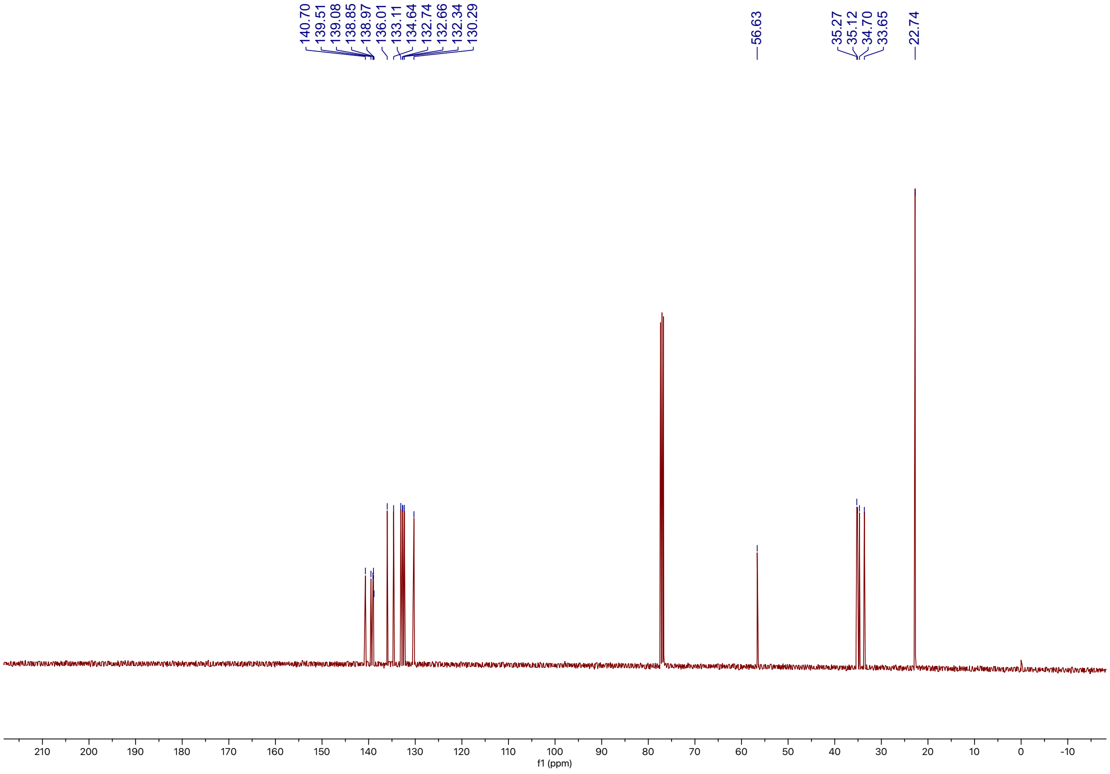

400 MHz ^{1}H NMR spectra of **S9** in $CDCl_3$

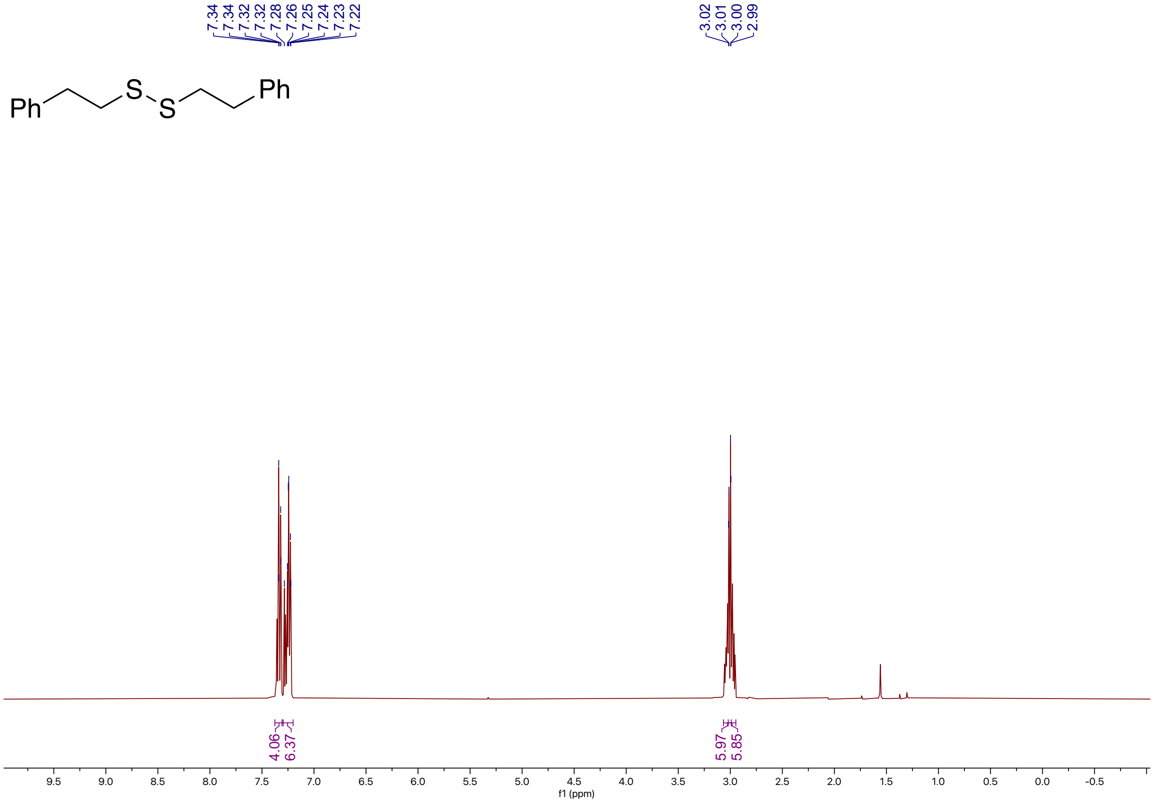

101 MHz $^{13}C\{^{1}H\}$ NMR spectrum of **S9** in $CDCl_3$

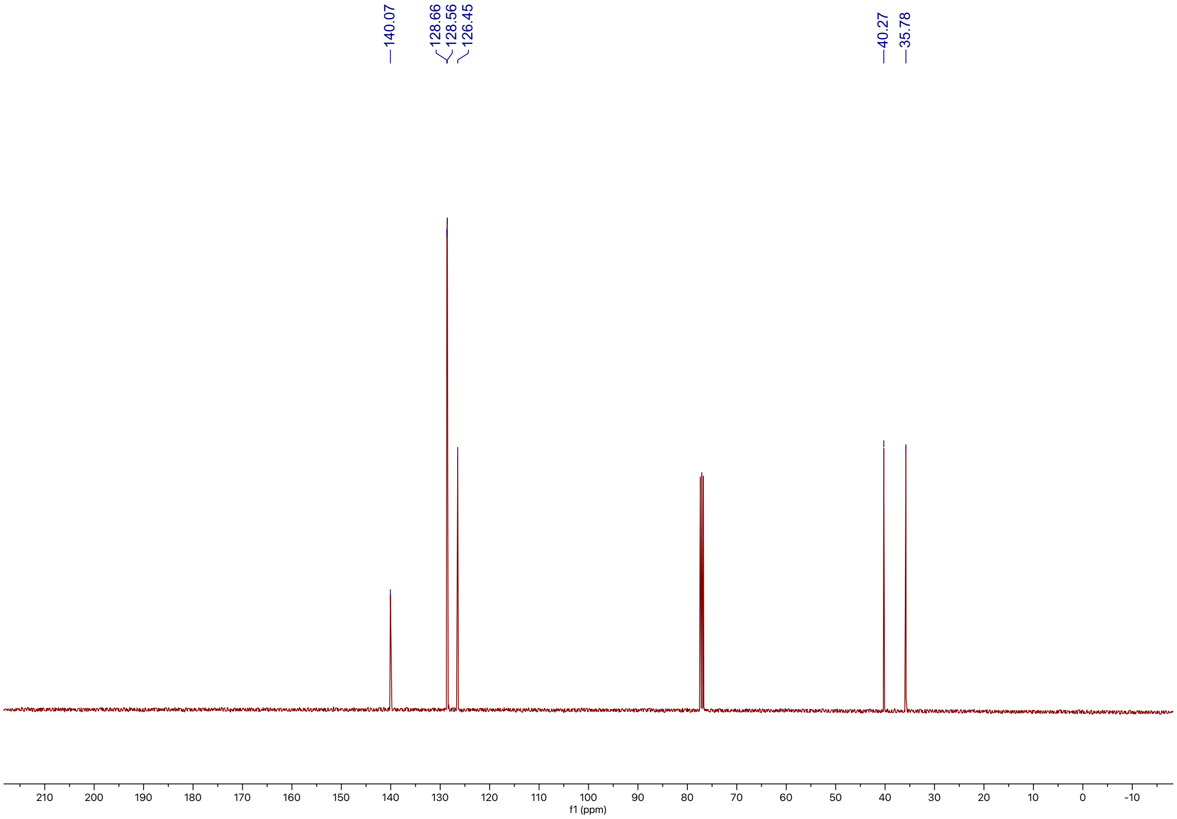

400 MHz ^{1}H NMR spectra of **S10** in CDCl$_3$

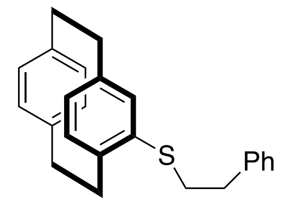

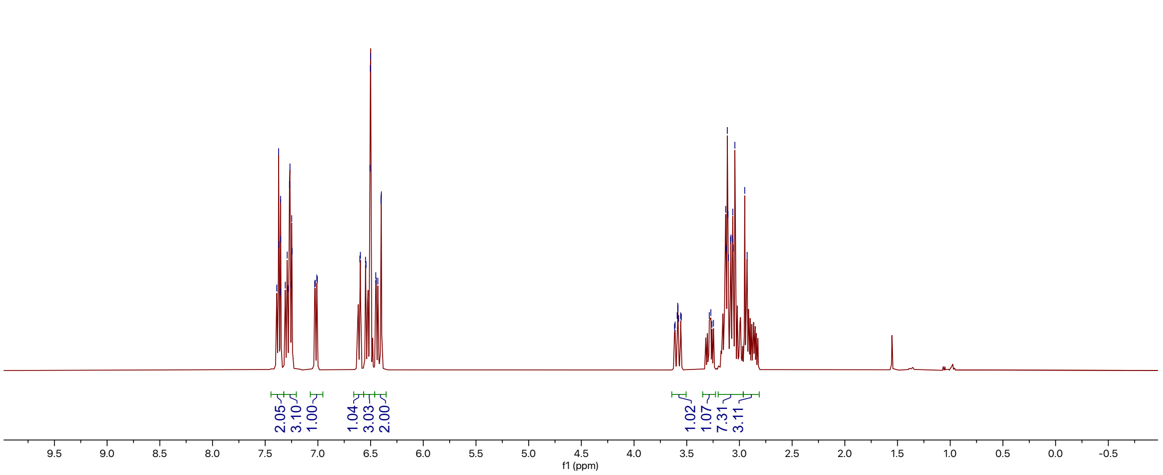

101 MHz ^{13}C{^{1}H} NMR spectrum of **S10** in CDCl$_3$

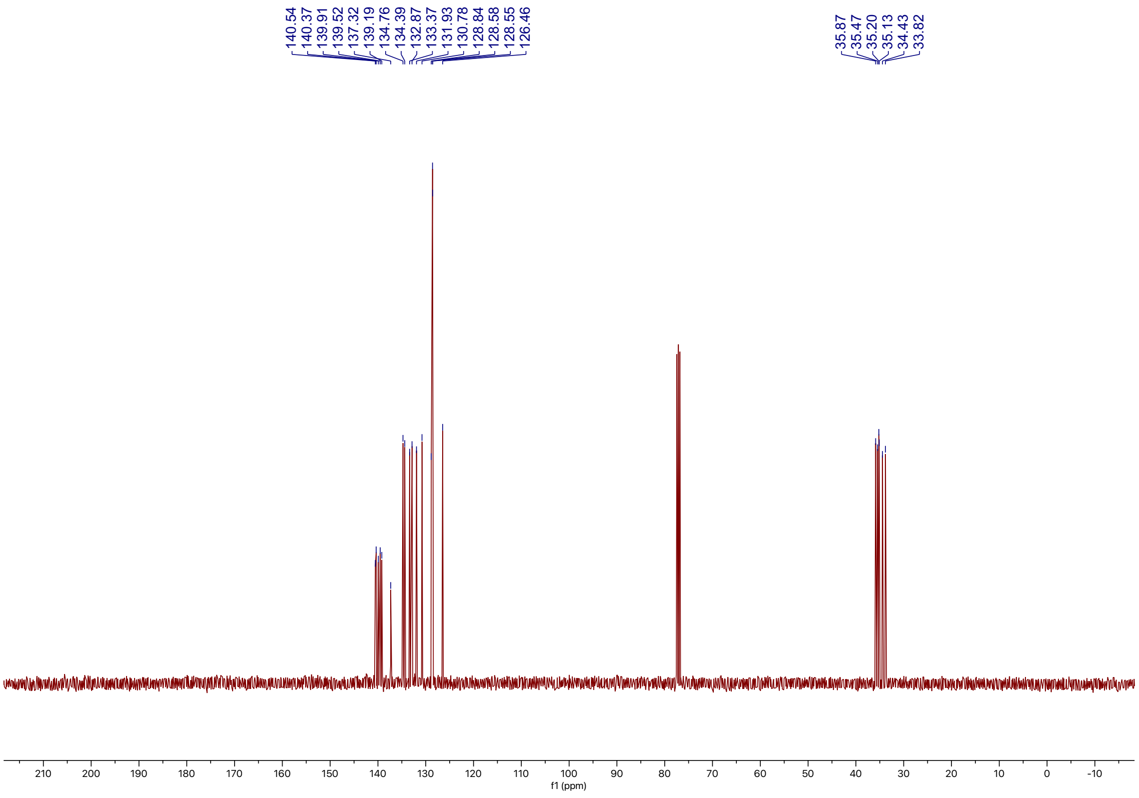

400 MHz ^{1}H NMR spectra of **A2-1** in $CDCl_3$

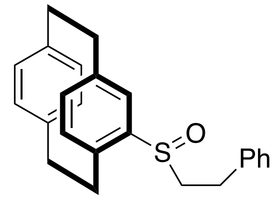

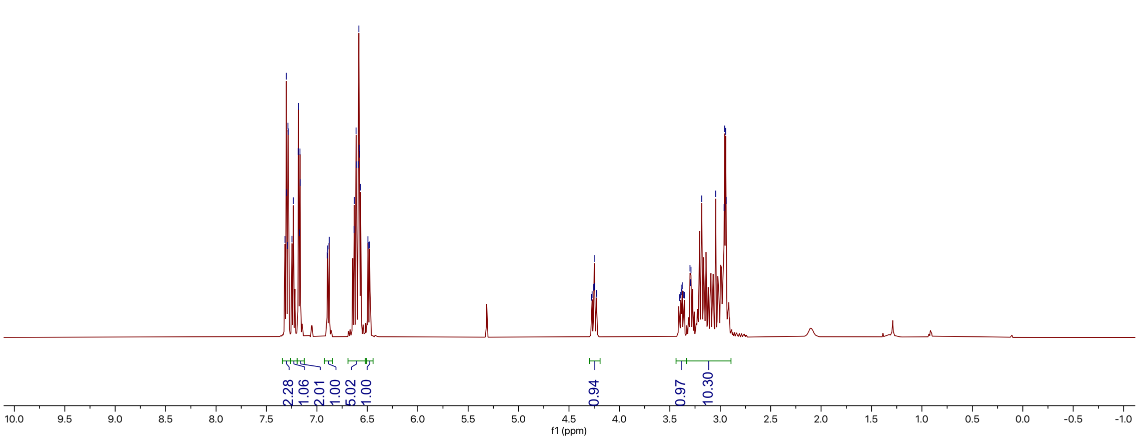

101 MHz $^{13}C\{^{1}H\}$ NMR spectrum of **A2-1** in $CDCl_3$

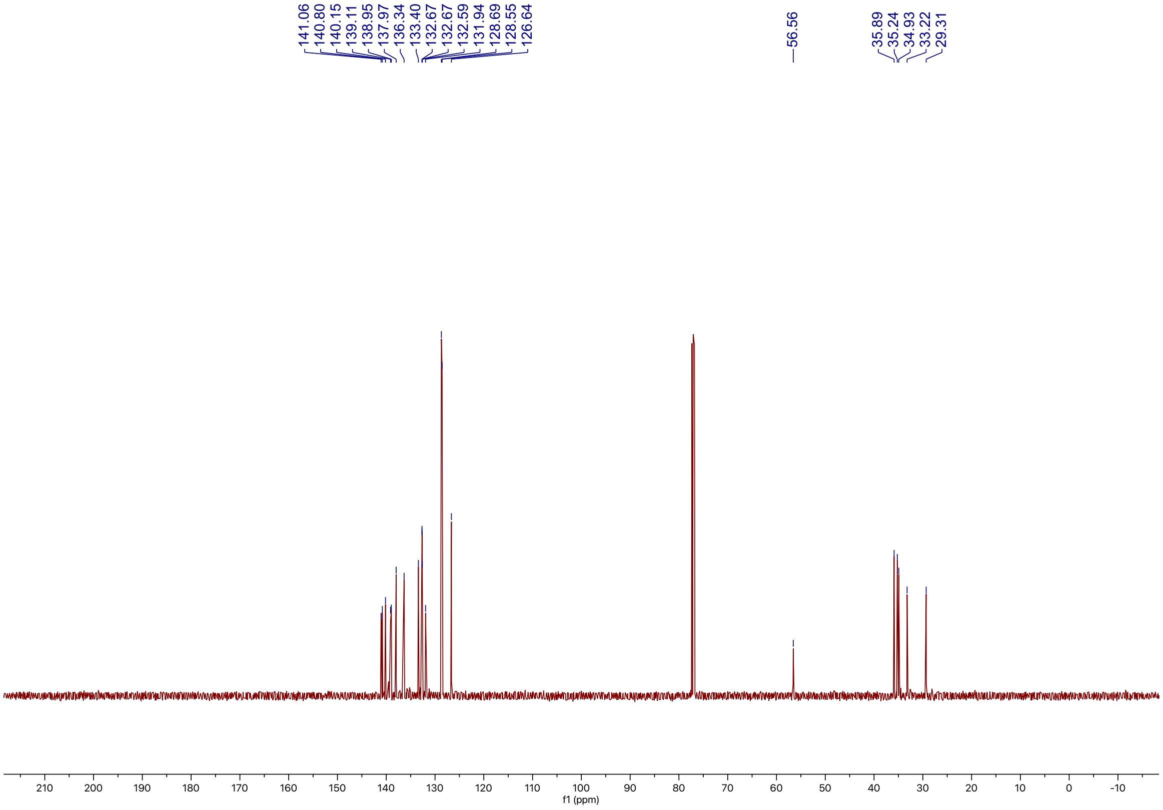

400 MHz ^{1}H NMR spectra of **A2-2** in $CDCl_3$

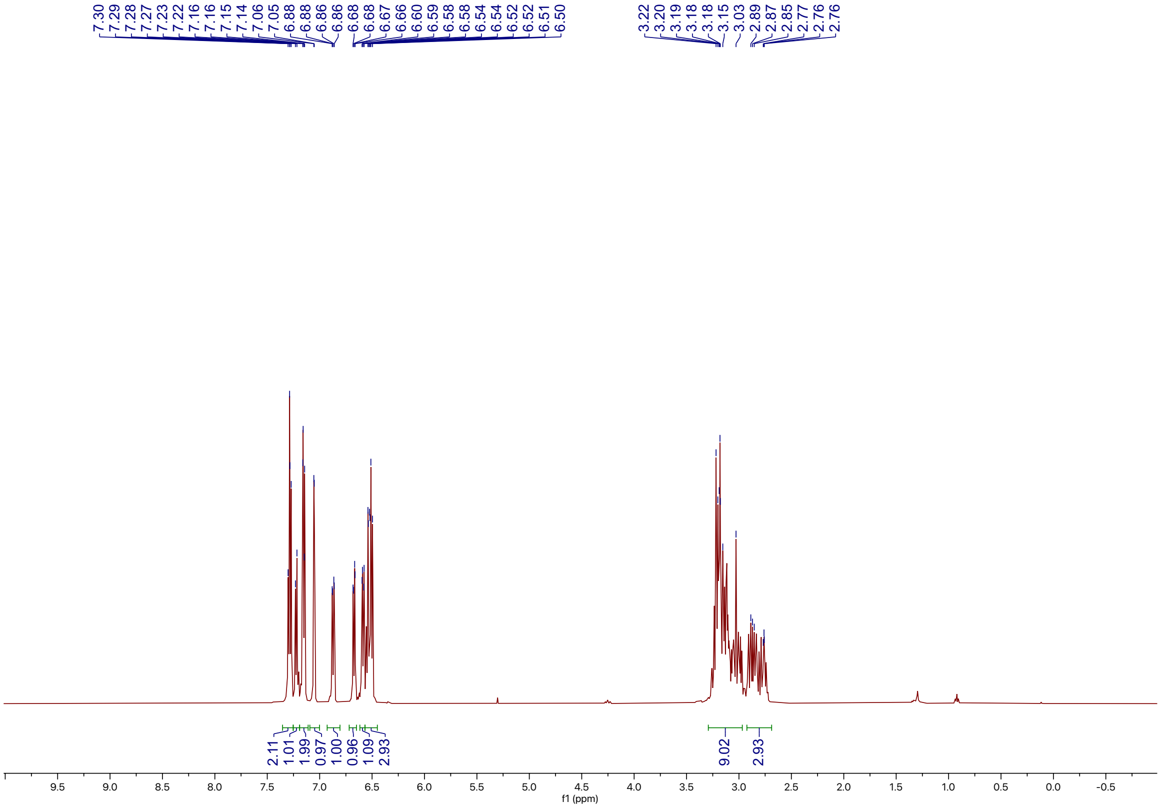

101 MHz $^{13}C\{^{1}H\}$ NMR spectrum of **A2-2** in $CDCl_3$

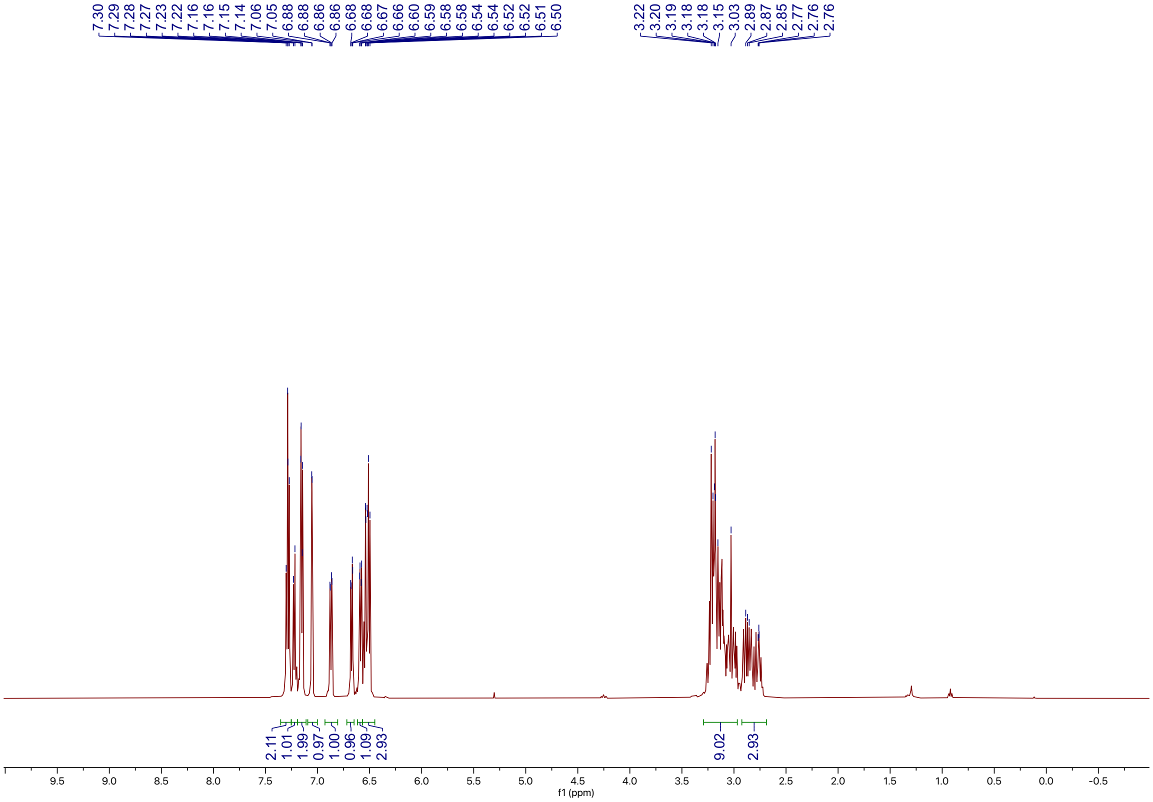

400 MHz ^{1}H NMR spectra of **3a** in $CDCl_3$

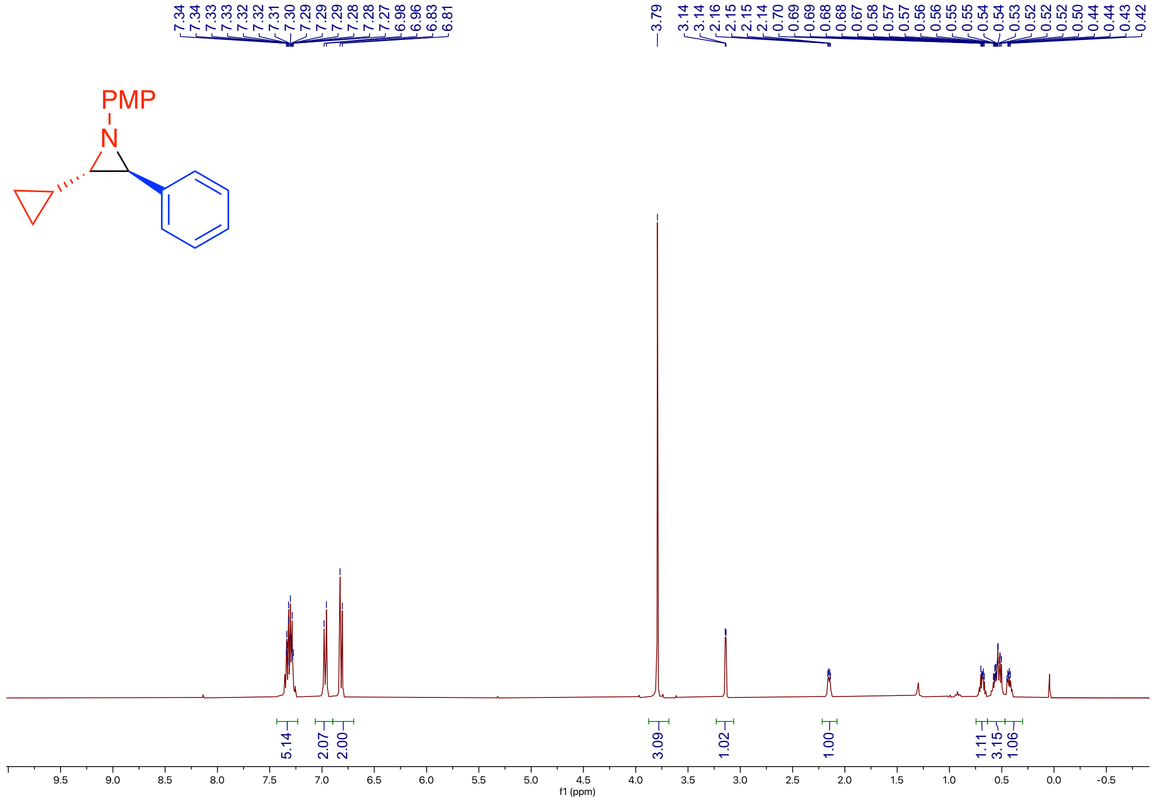

101 MHz $^{13}C\{^1H\}$ NMR spectrum of **3a** in $CDCl_3$

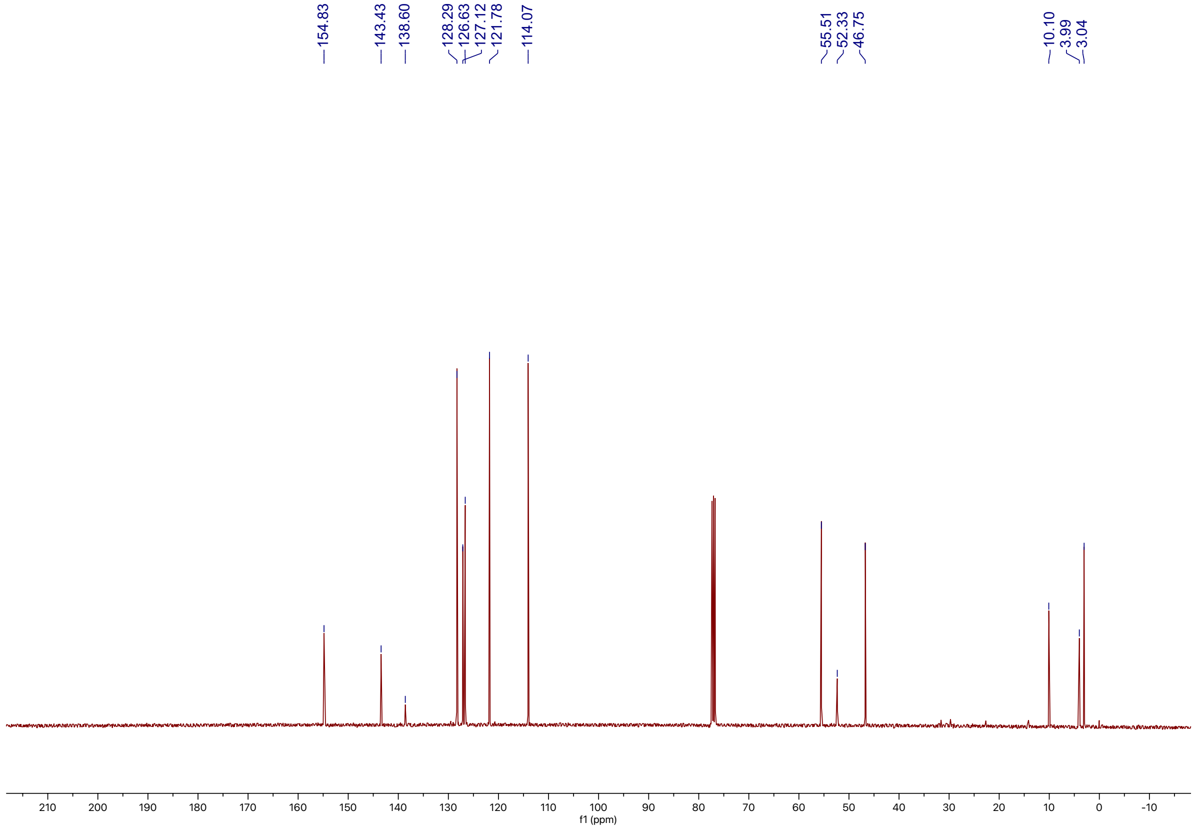

400 MHz ^{1}H NMR spectra of **3b** in $CDCl_3$

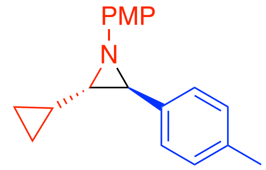

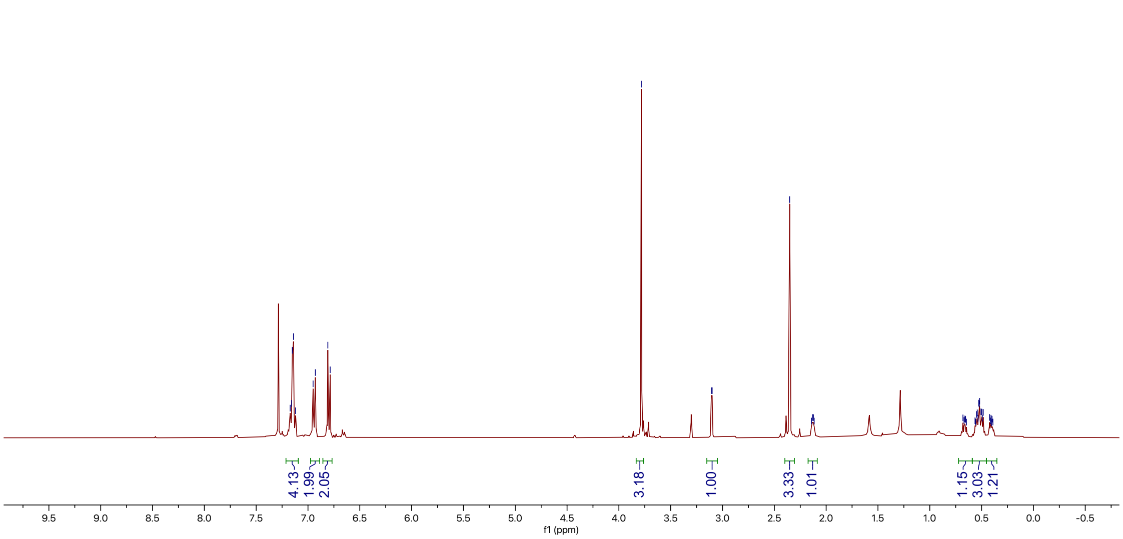

101 MHz $^{13}C\{^{1}H\}$ NMR spectrum of **3b** in $CDCl_3$

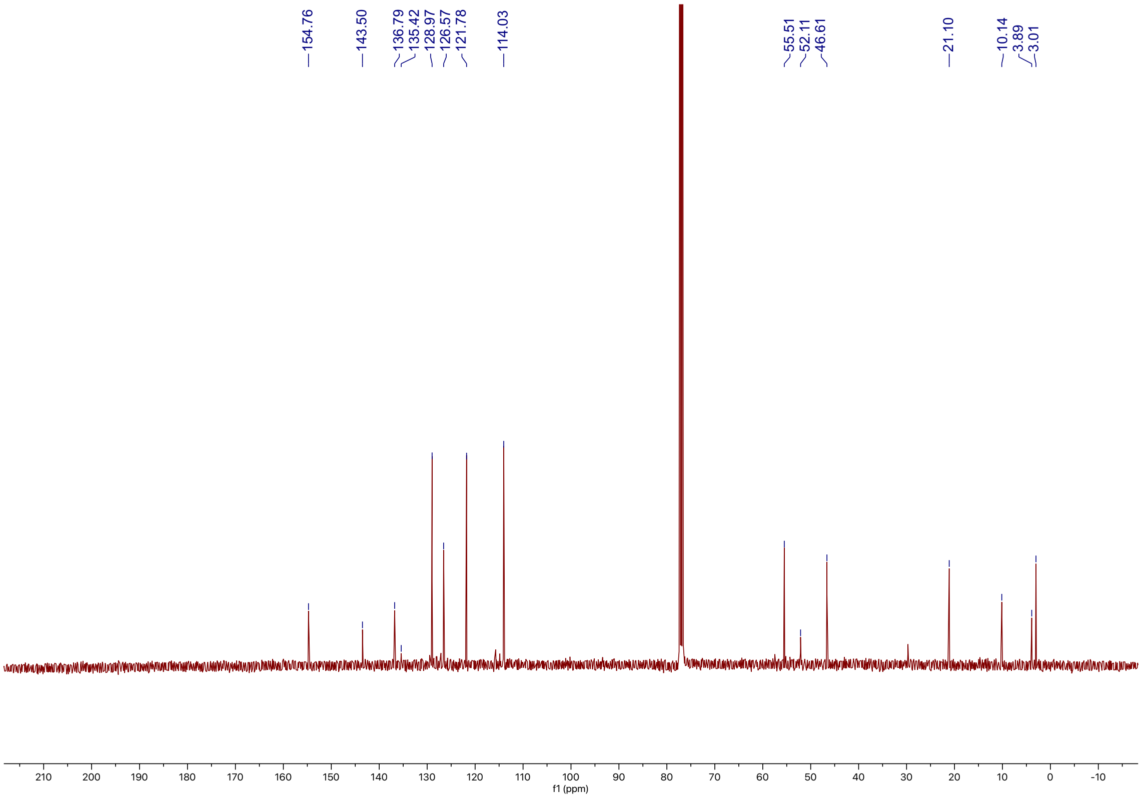

400 MHz ^{1}H NMR spectra of **3c** in $CDCl_3$

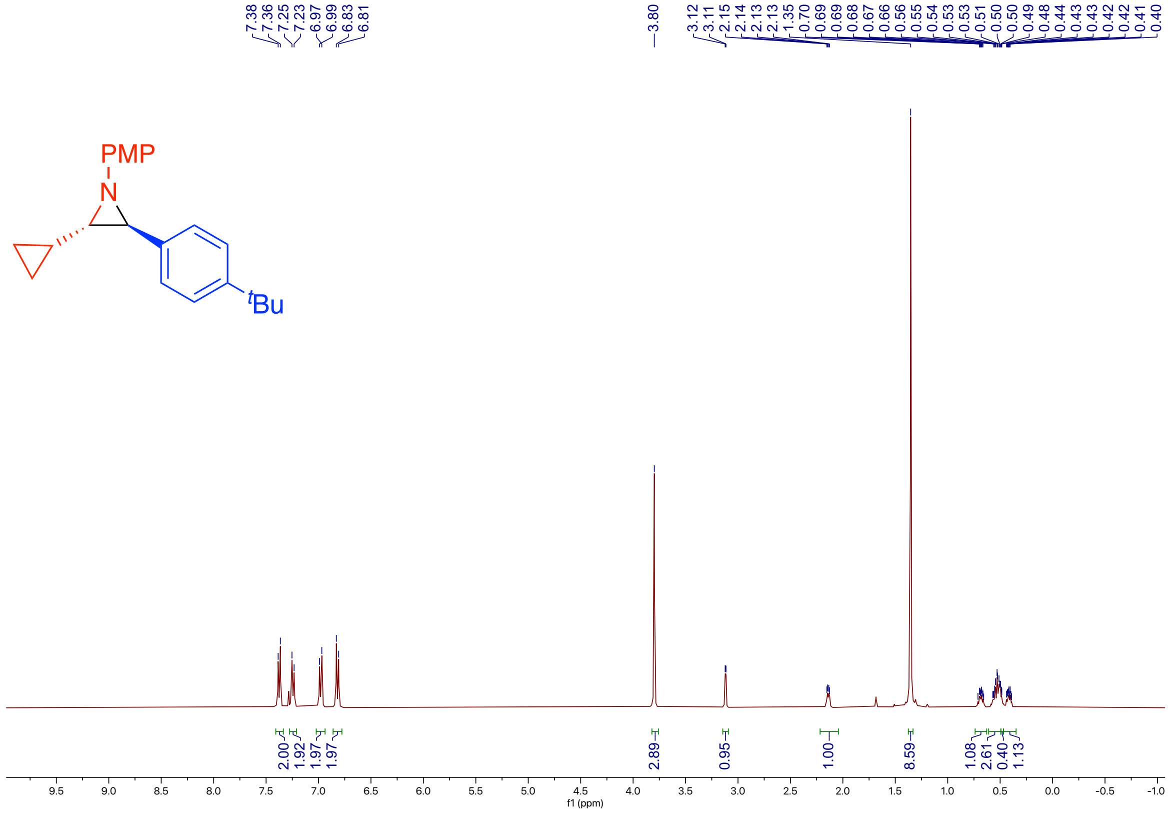

101 MHz $^{13}C\{^1H\}$ NMR spectrum of **3c** in $CDCl_3$

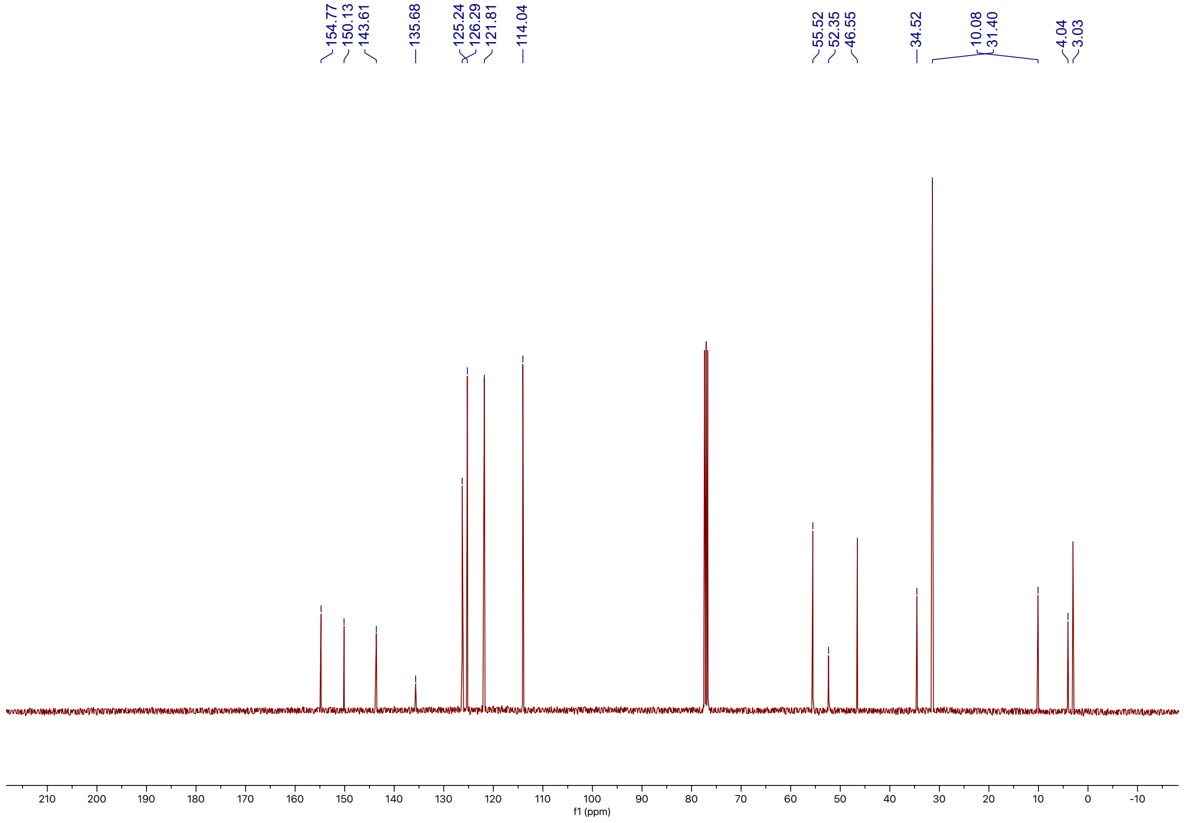

400 MHz ^{1}H NMR spectra of **3d** in $CDCl_3$

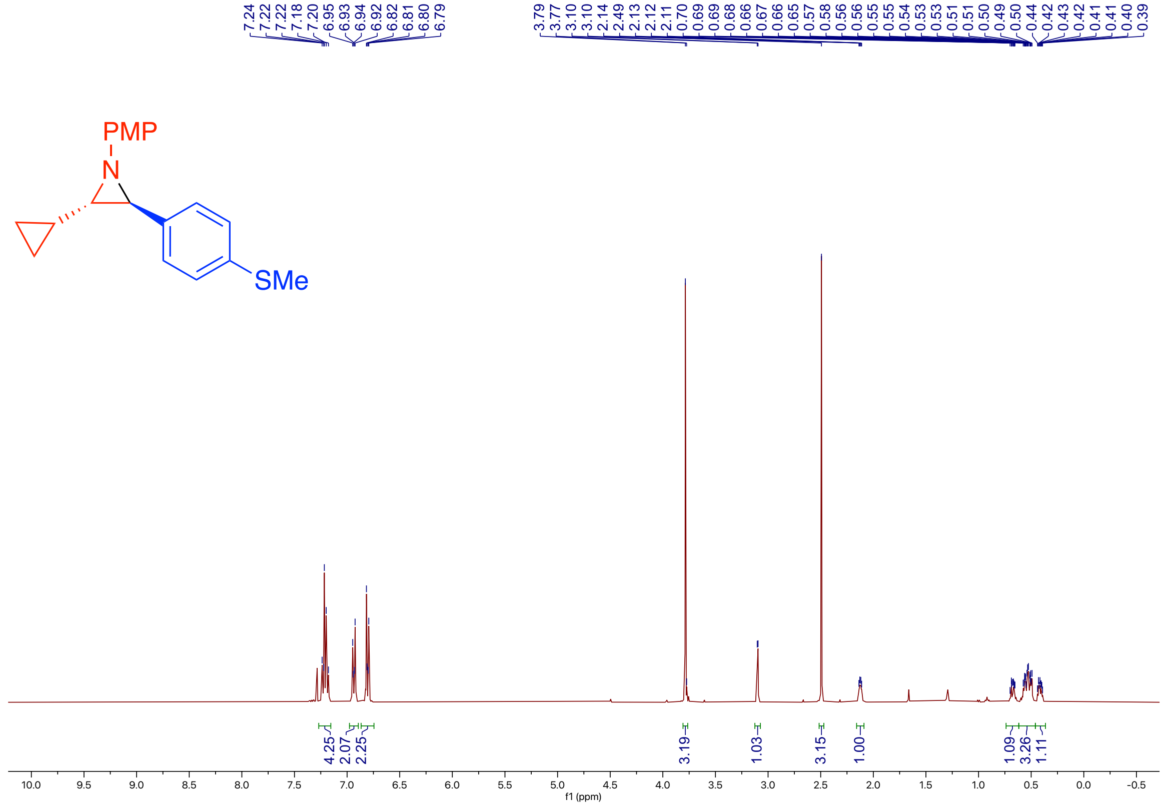

101 MHz ^{13}C{^{1}H} NMR spectrum of **3d** in $CDCl_3$

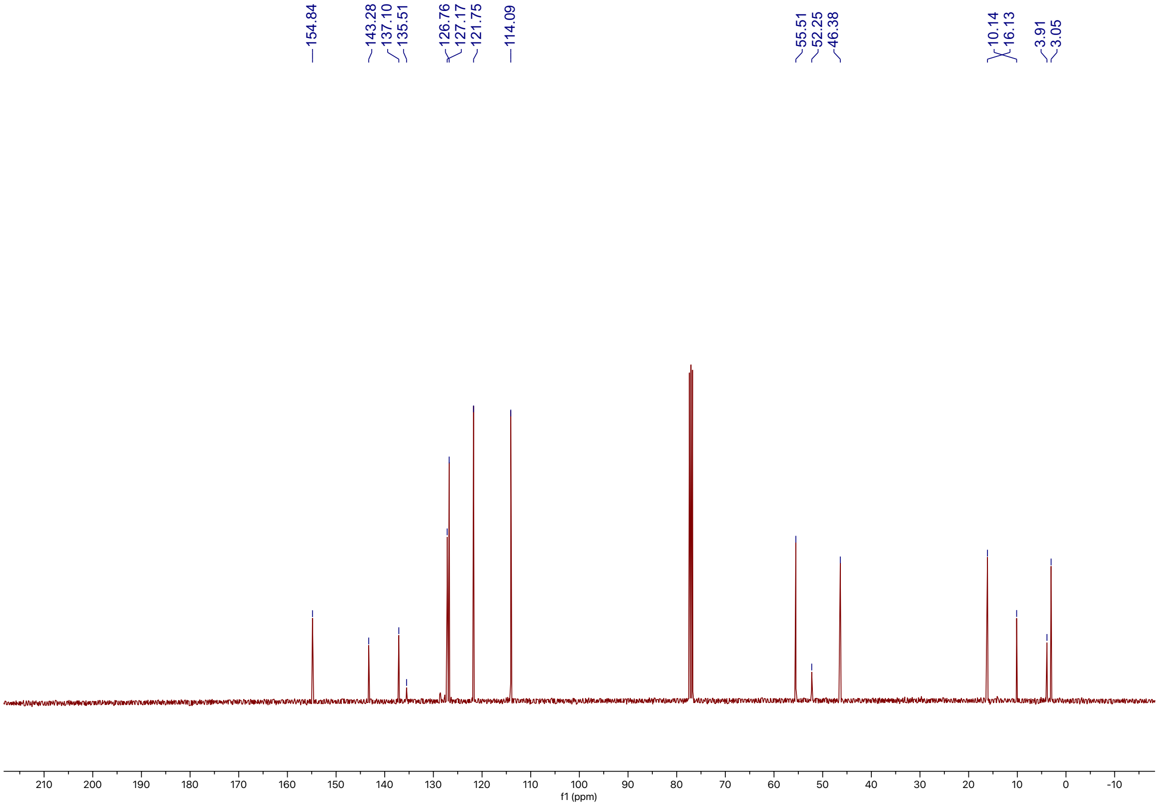

400 MHz ^{1}H NMR spectra of **3e** in $CDCl_3$

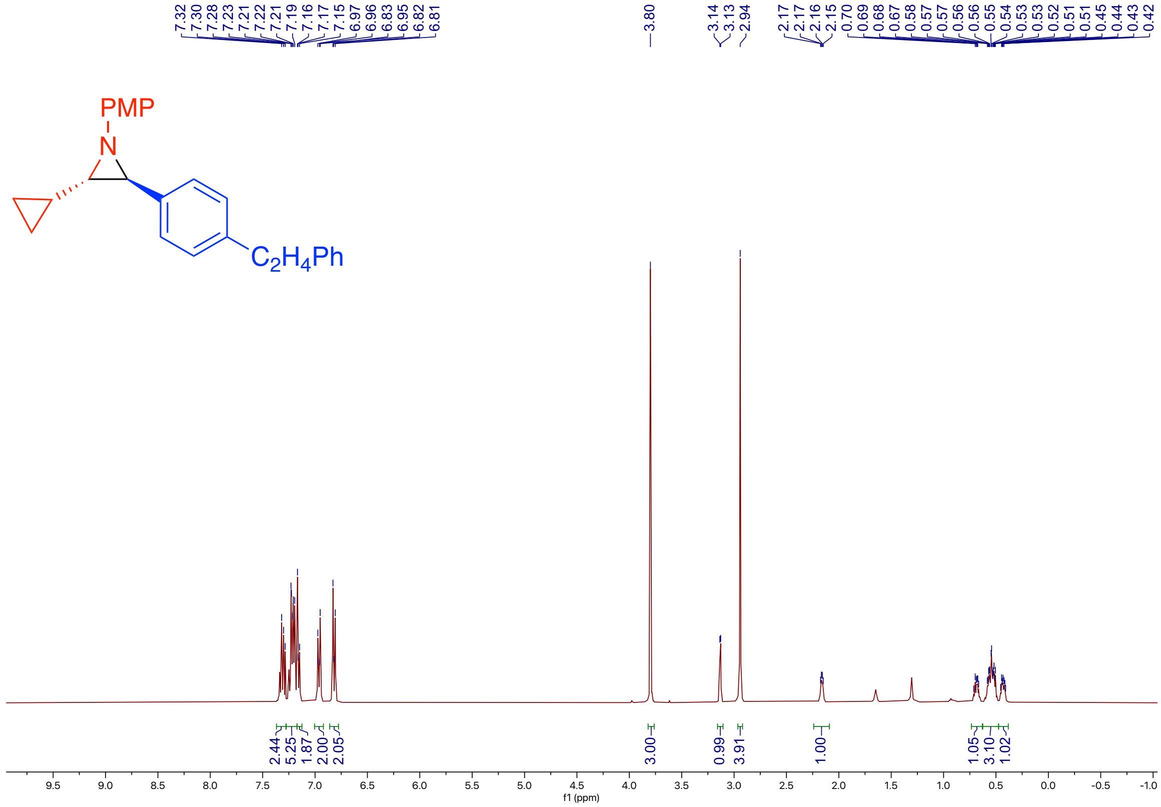

101 MHz $^{13}C\{^{1}H\}$ NMR spectrum of **3e** in $CDCl_3$

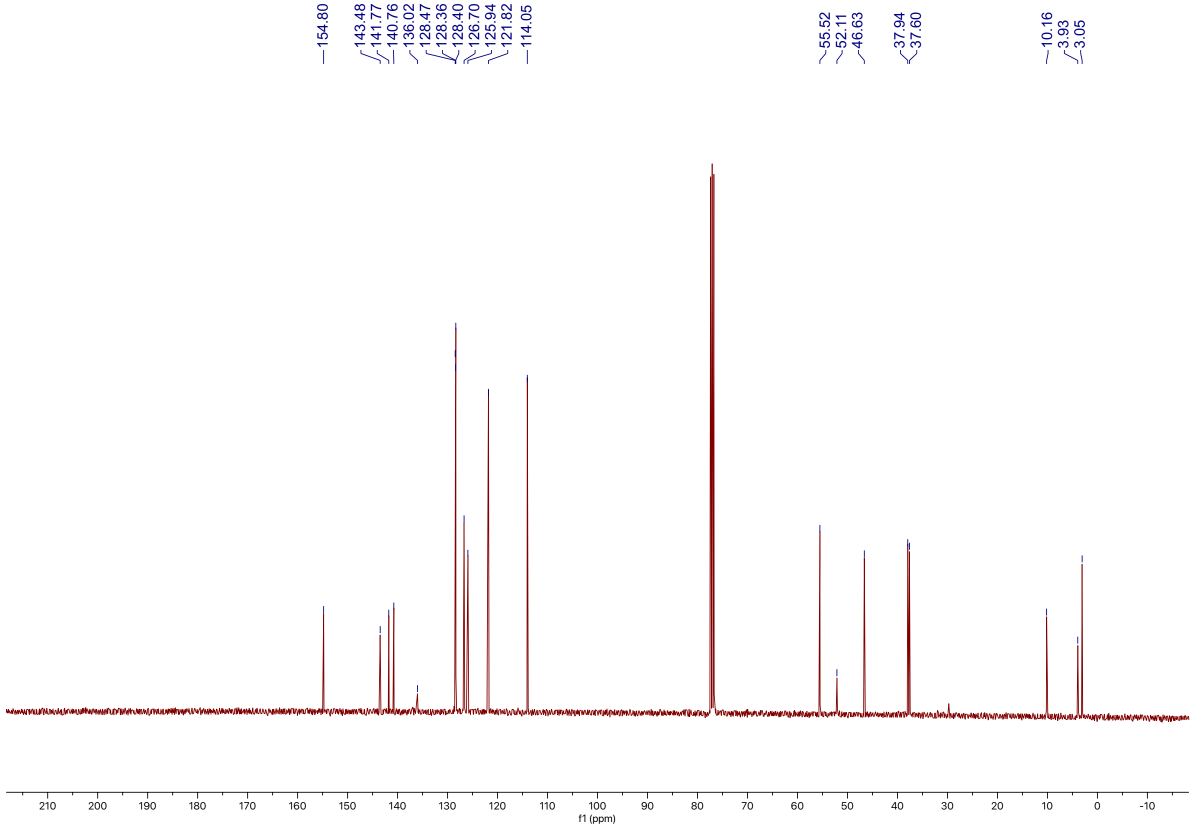

400 MHz ^{1}H NMR spectra of **3f** in $CDCl_3$

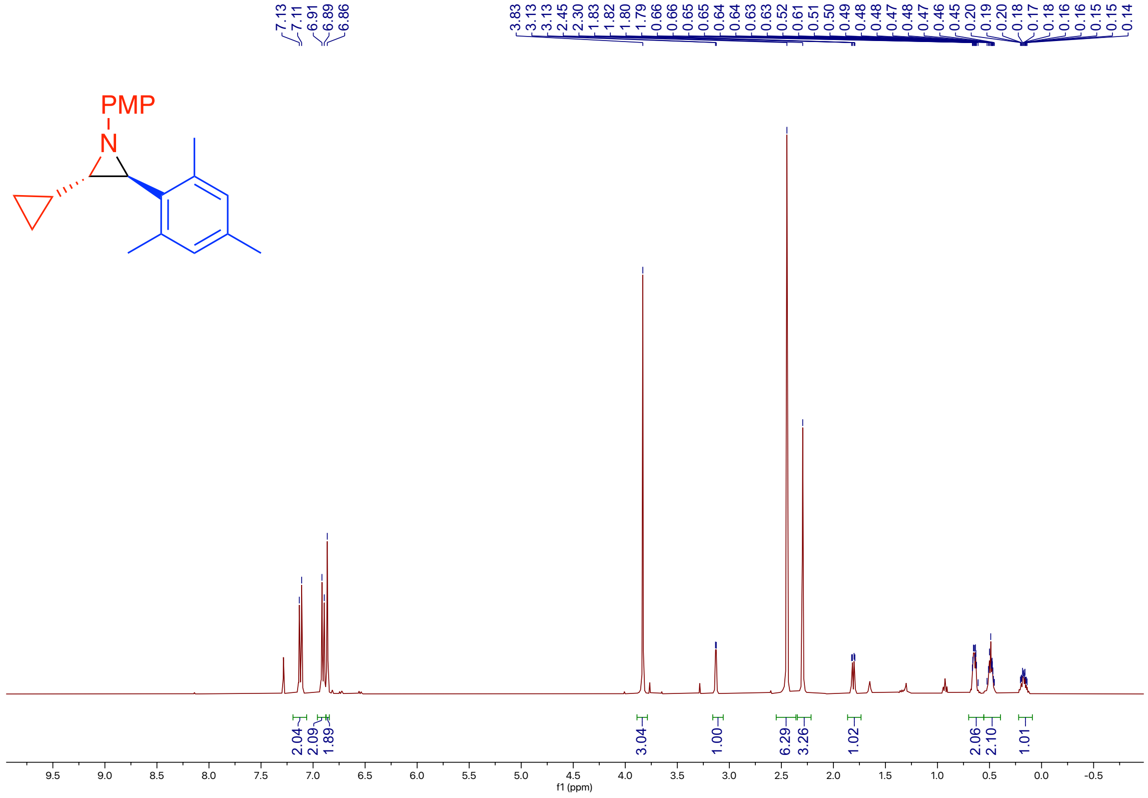

101 MHz $^{13}C\{^{1}H\}$ NMR spectrum of **3f** in $CDCl_3$

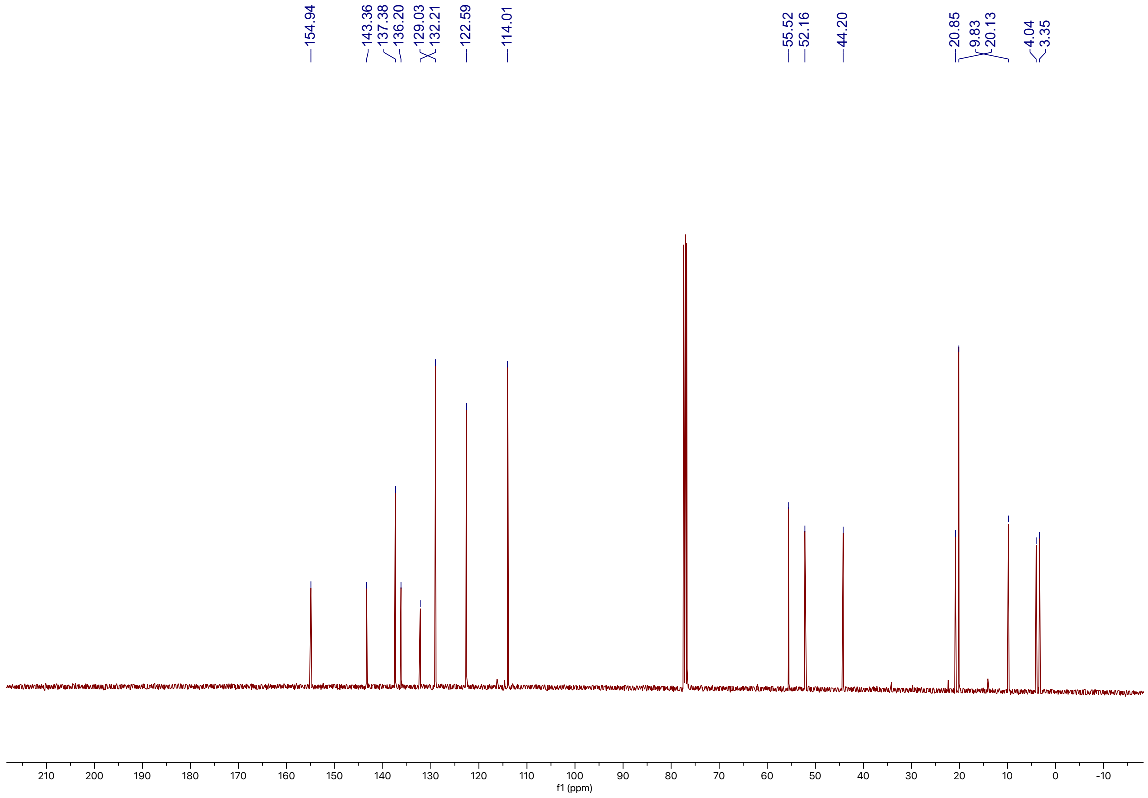

400 MHz ^{1}H NMR spectra of **3g** in $CDCl_3$

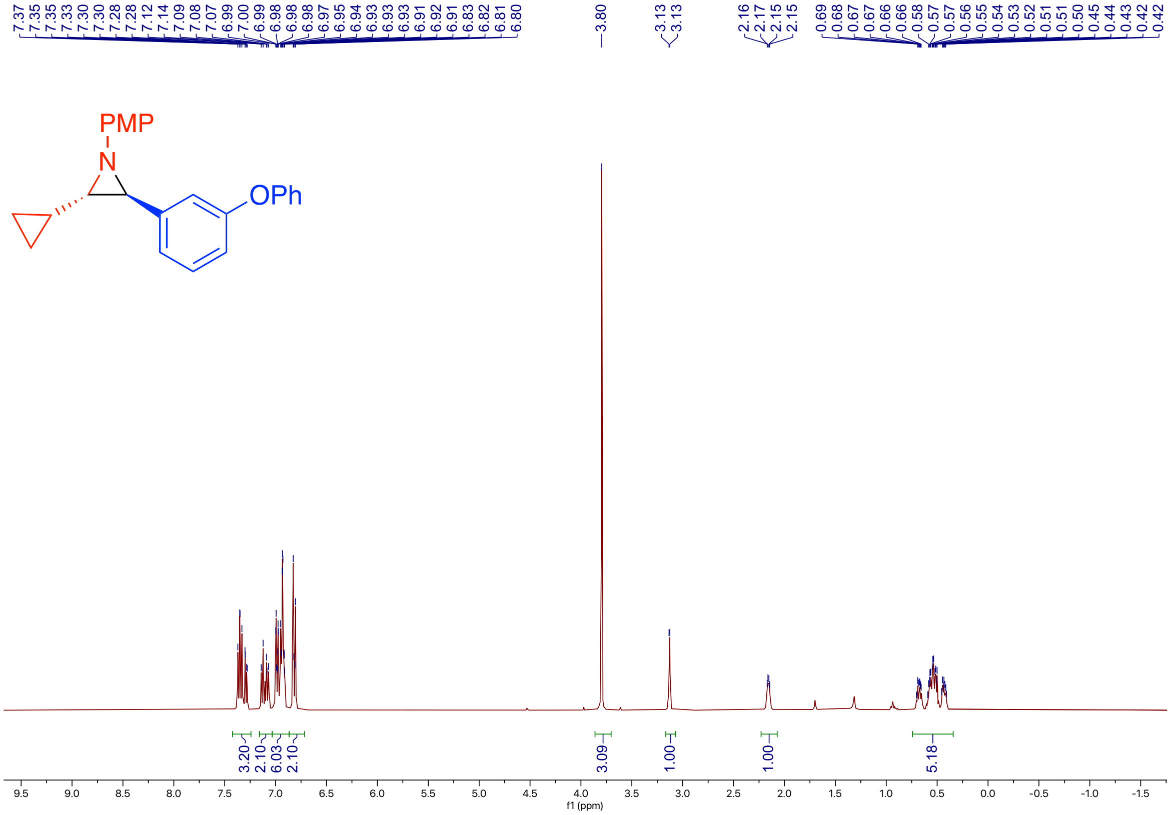

101 MHz $^{13}C\{^{1}H\}$ NMR spectrum of **3g** in $CDCl_3$

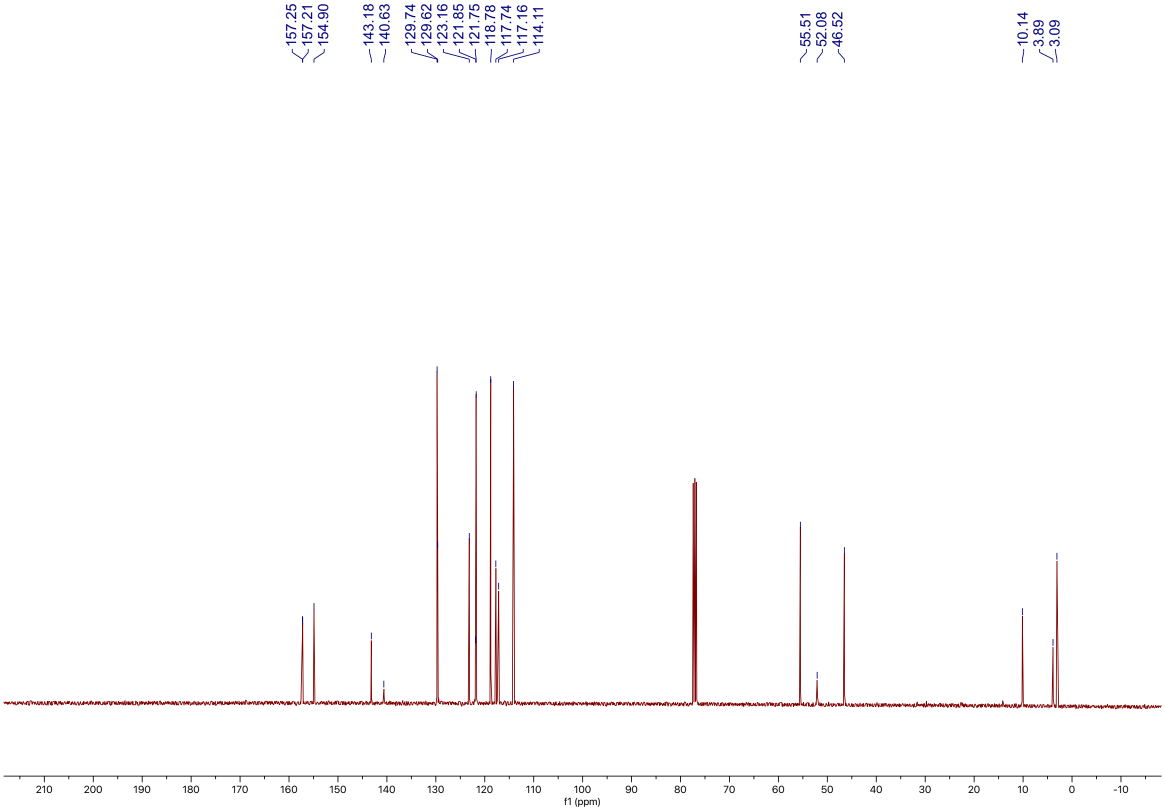

400 MHz ^{1}H NMR spectra of **3h** in $CDCl_3$

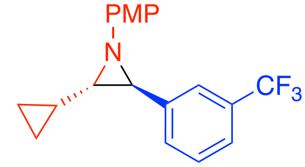

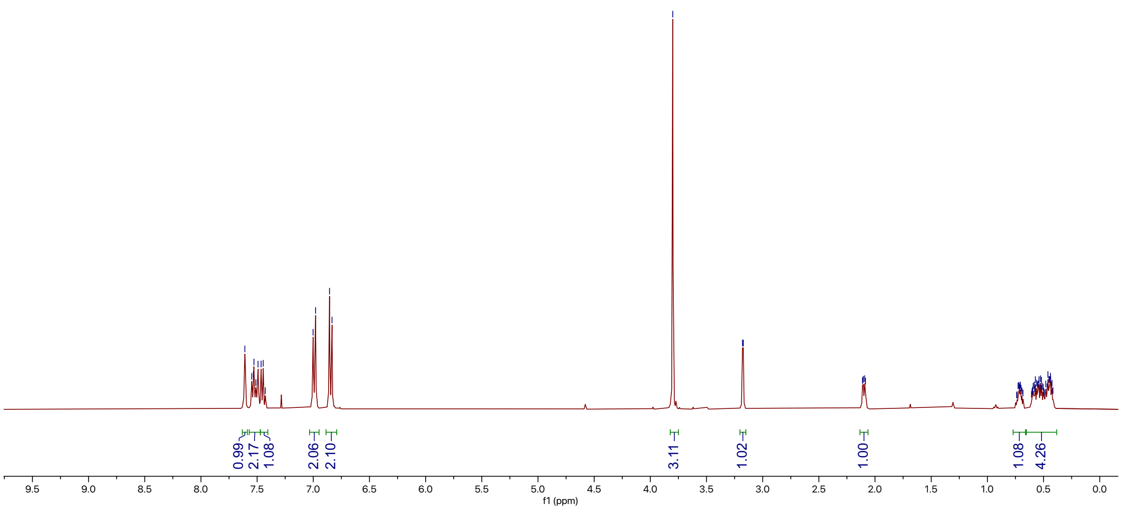

101 MHz $^{13}C\{^{1}H\}$ NMR spectrum of **3h** in $CDCl_3$

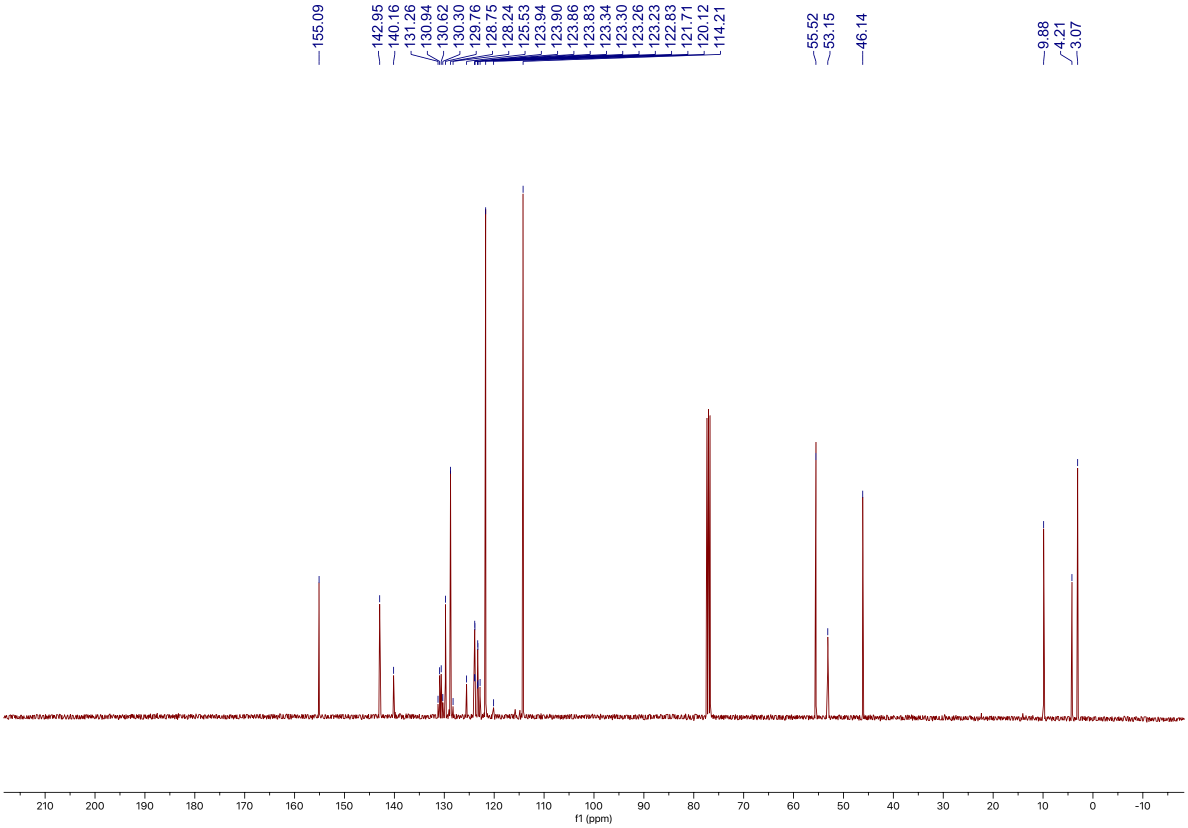

376 MHz $^{19}F\{^{1}H\}$ NMR spectrum of **3h** in $CDCl_3$

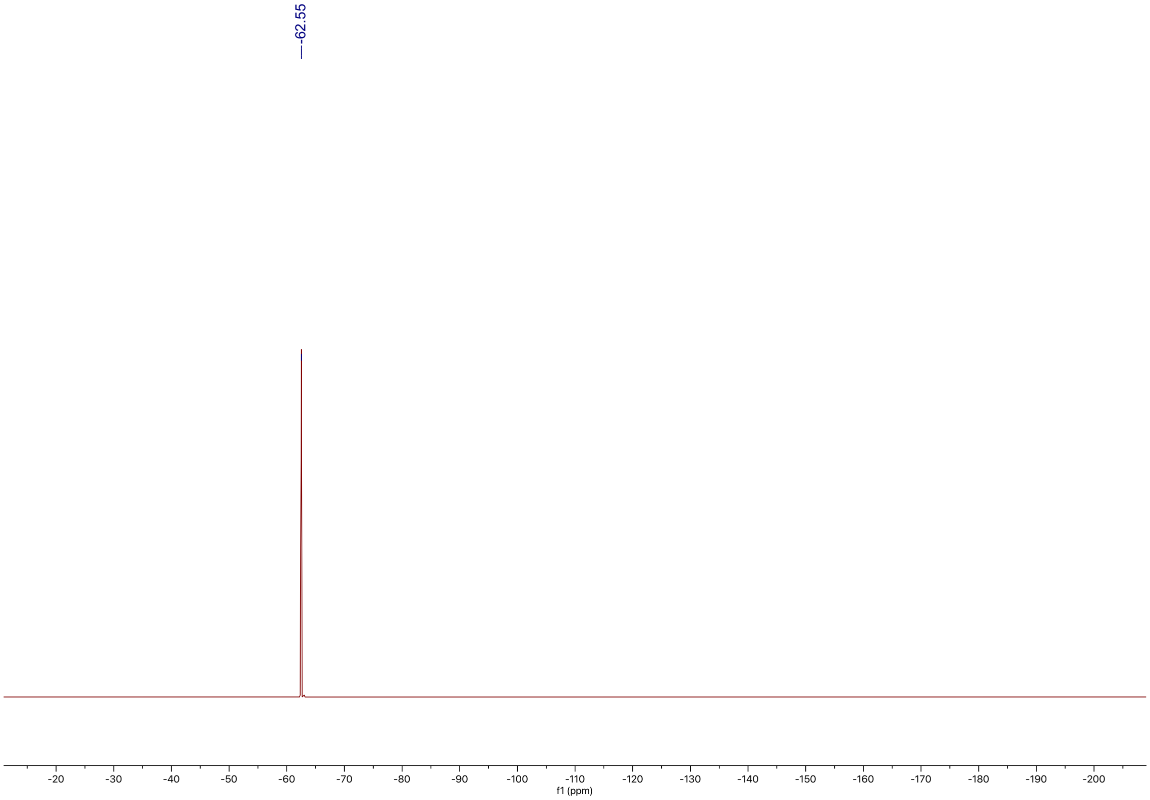

400 MHz ^{1}H NMR spectra of **3i** in $CDCl_3$

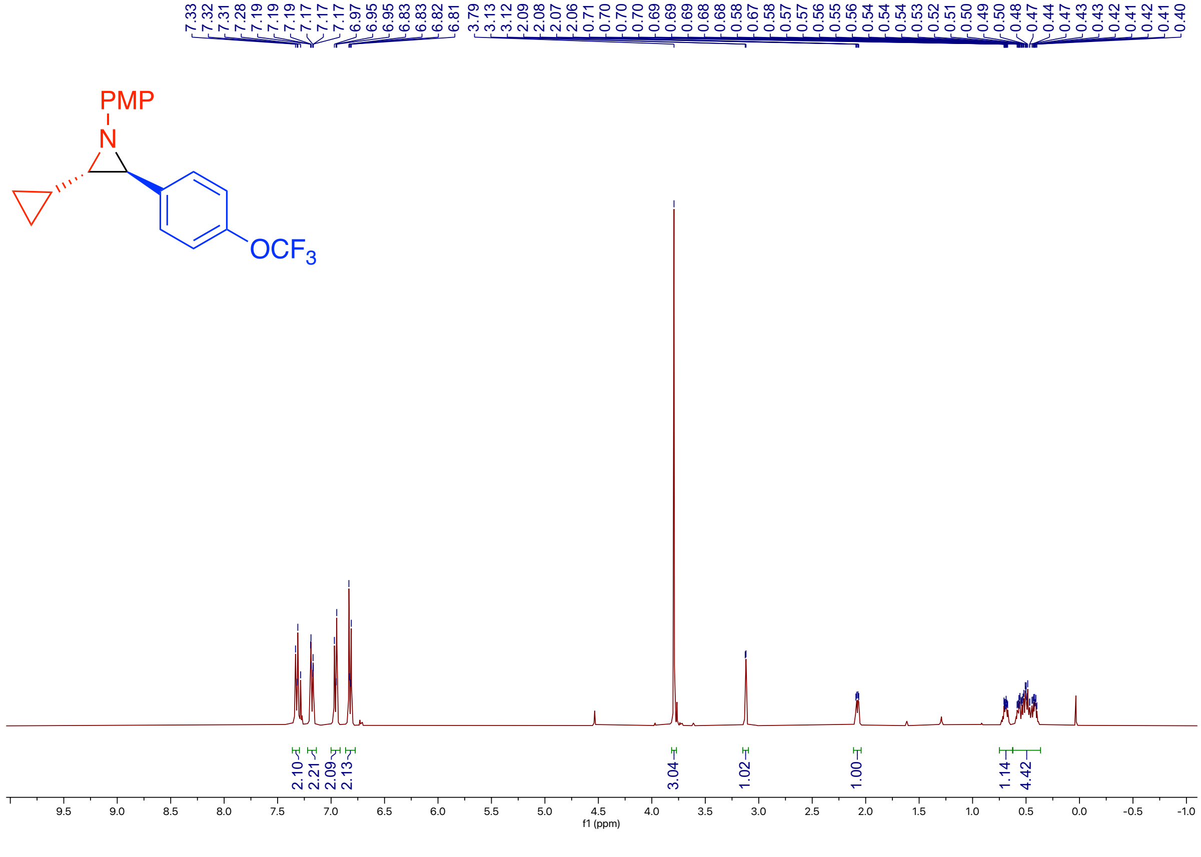

101 MHz $^{13}C\{^{1}H\}$ NMR spectrum of **3i** in $CDCl_3$

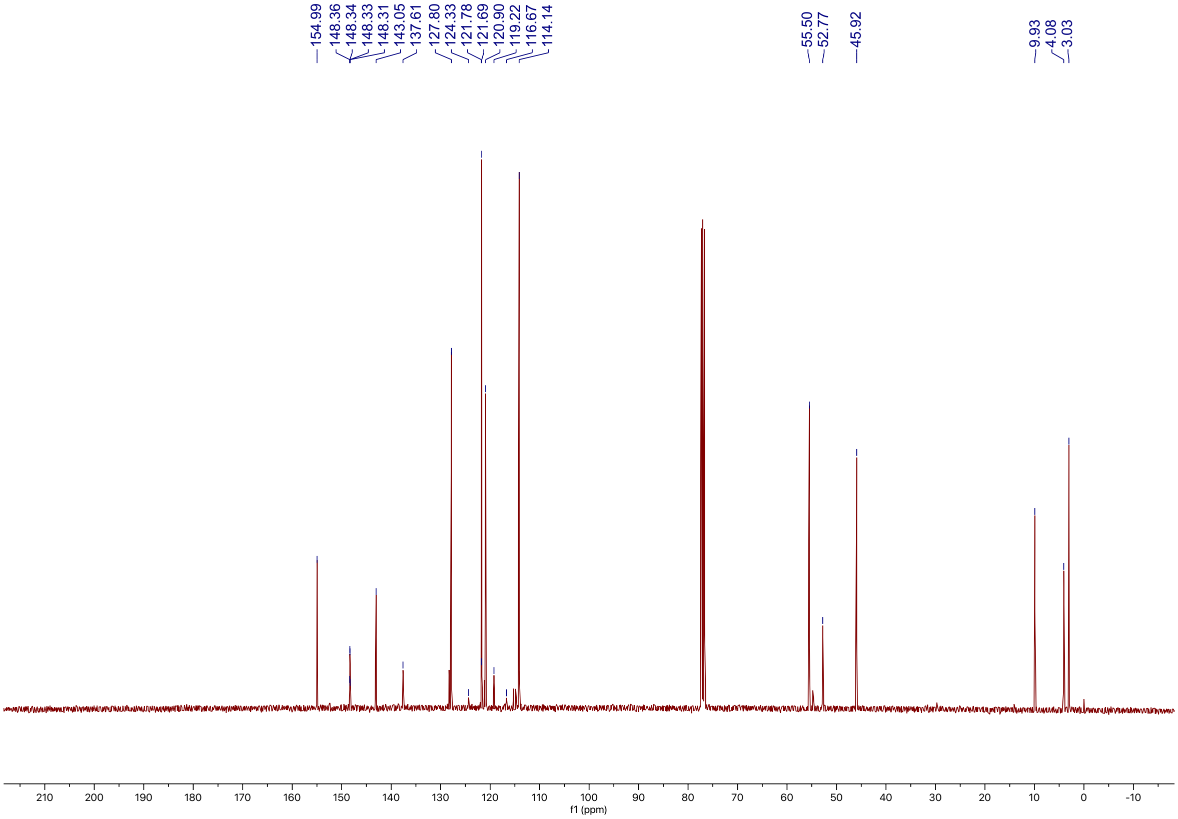

376 MHz $^{19}F\{^{1}H\}$ NMR spectrum of **3i** in $CDCl_3$

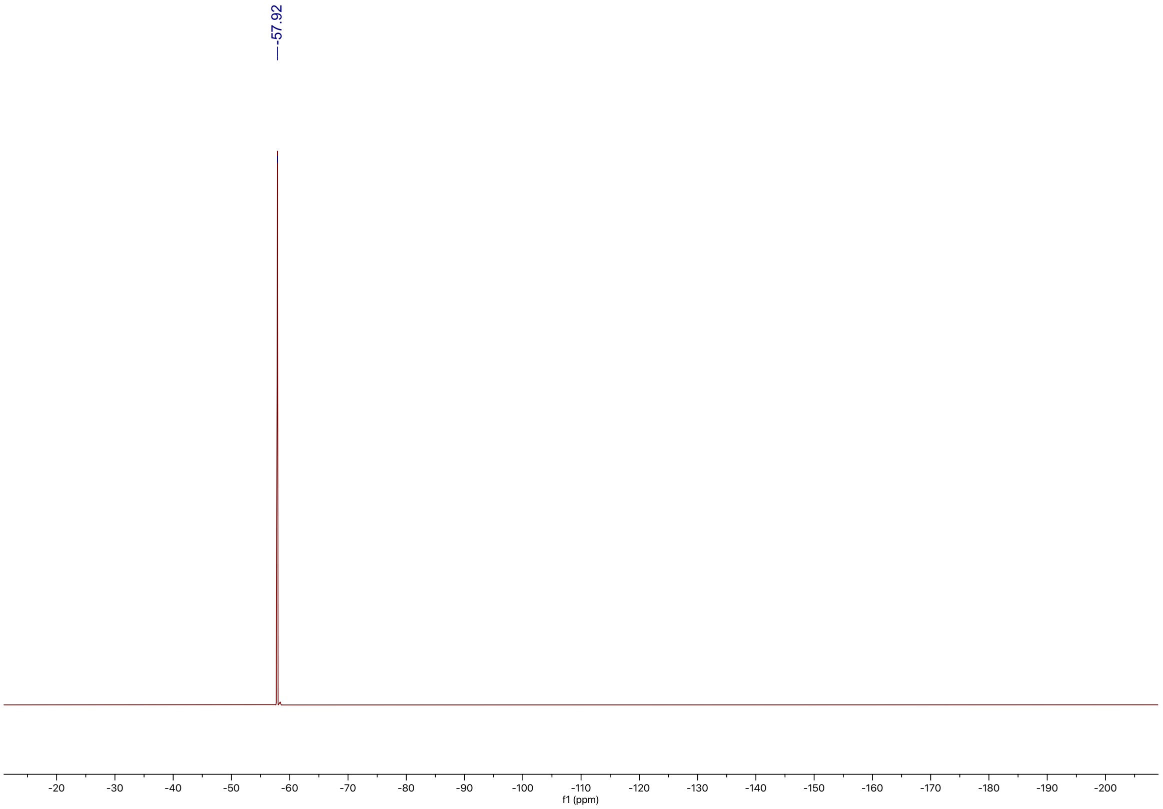

400 MHz ¹H NMR spectra of **3j** in $CDCl_3$

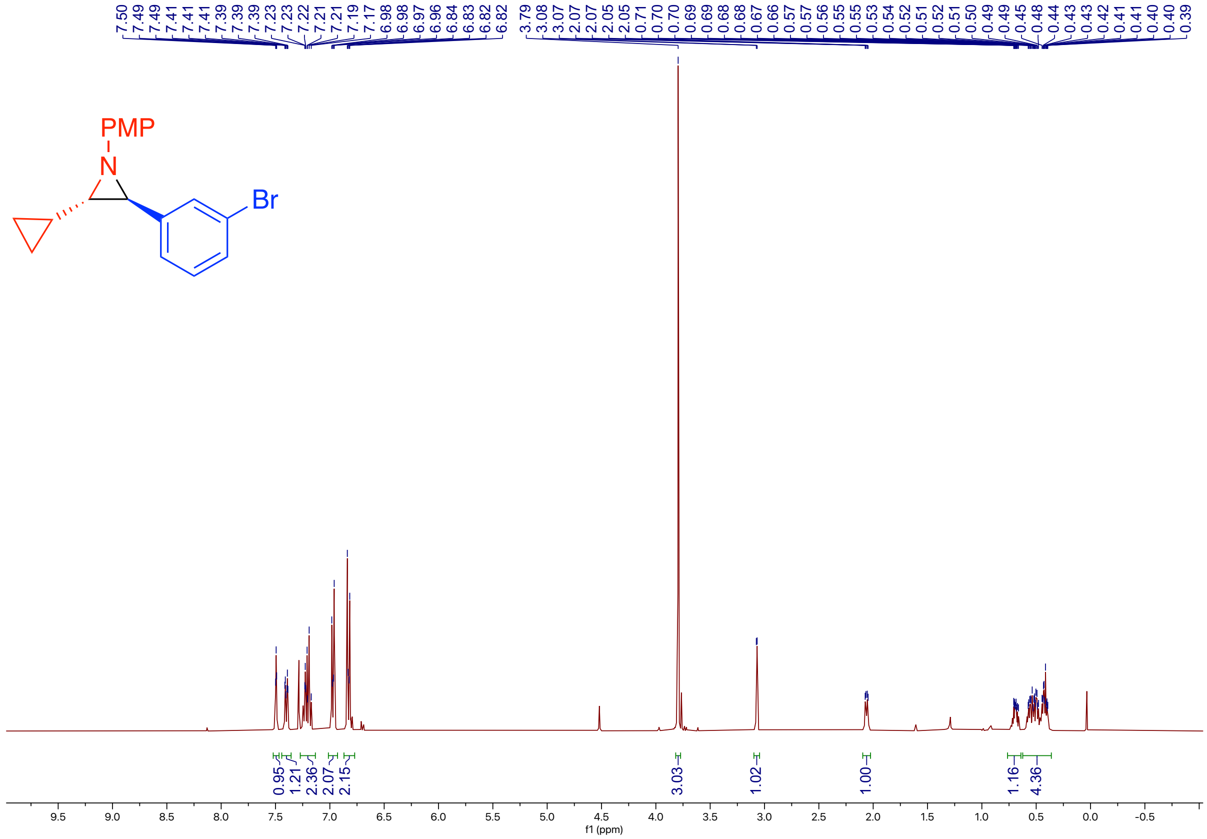

101 MHz ¹³C{¹H} NMR spectrum of **3j** in $CDCl_3$

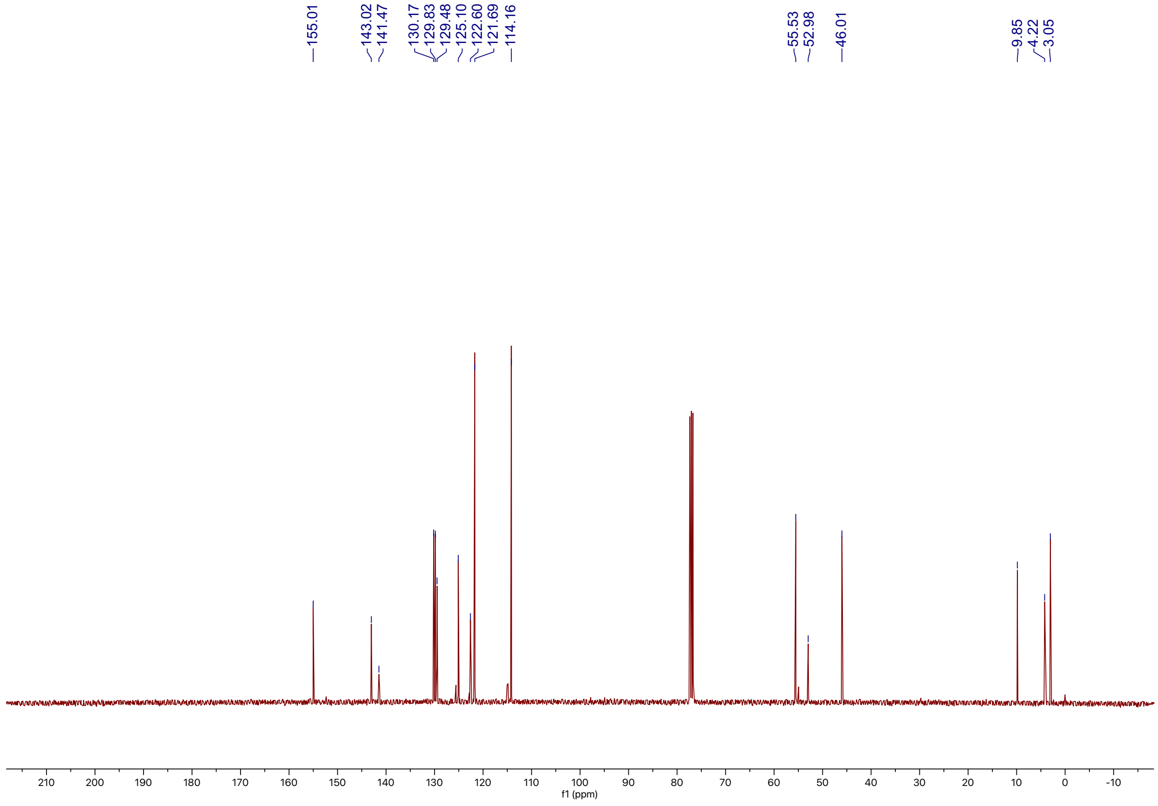

400 MHz ^{1}H NMR spectra of **3k** in $CDCl_3$

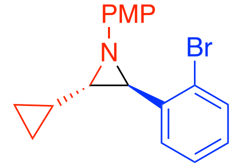

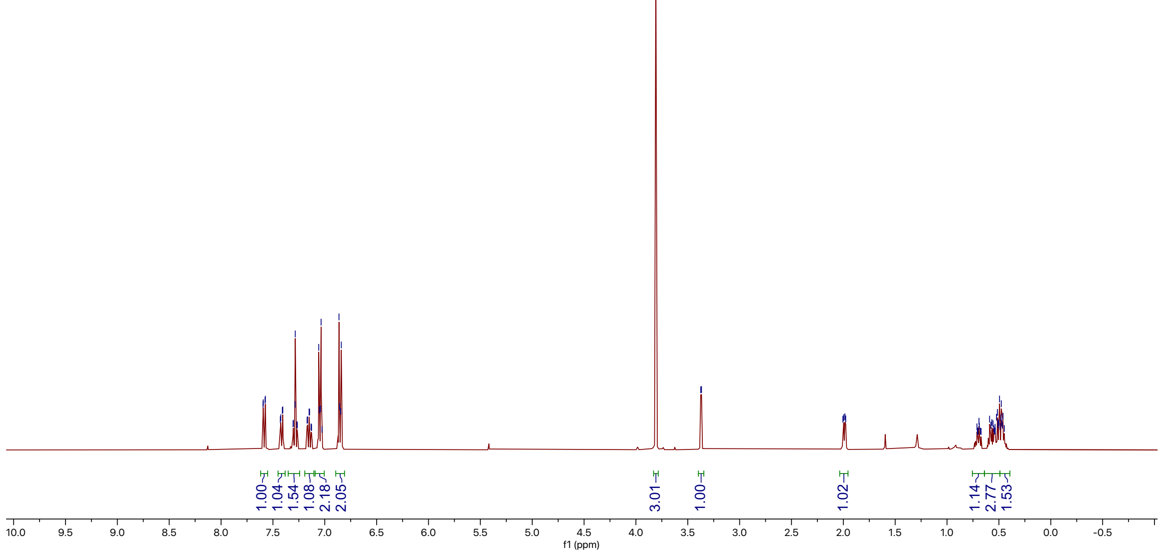

101 MHz ^{13}C{^{1}H} NMR spectrum of **3k** in $CDCl_3$

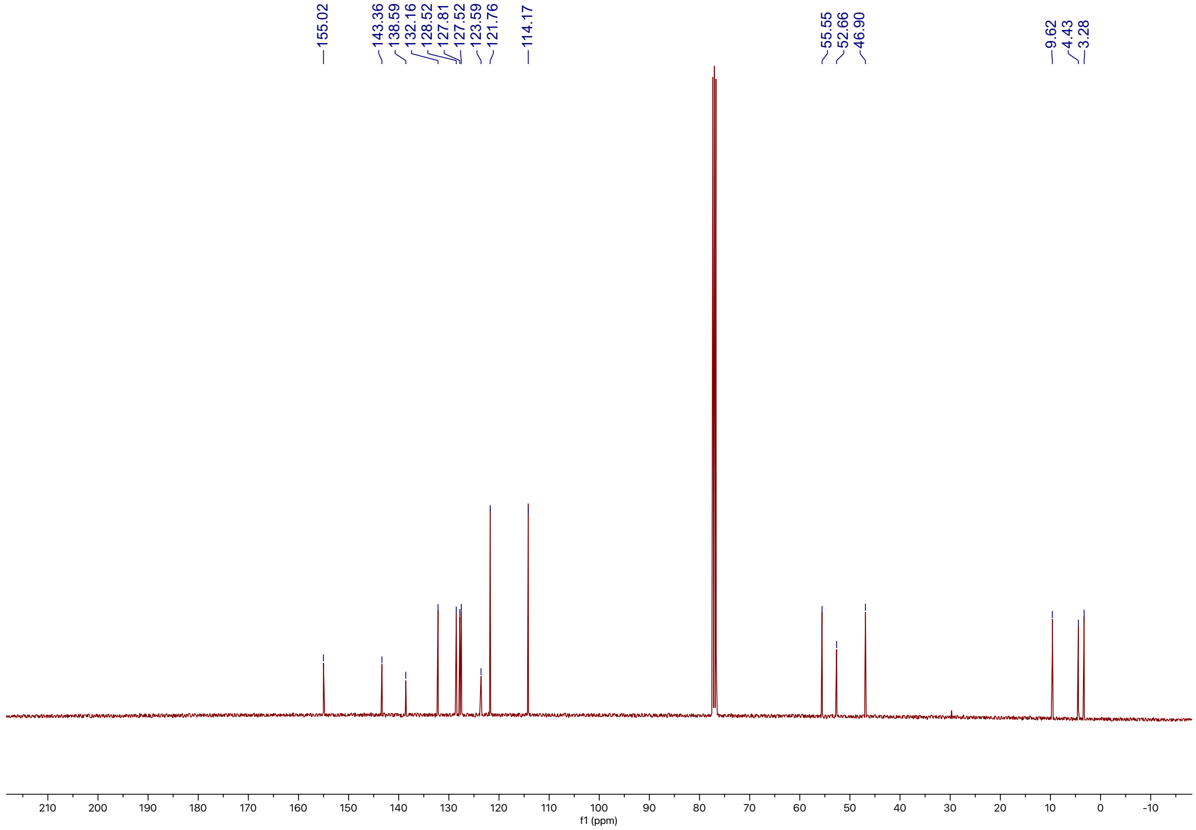

400 MHz ¹H NMR spectra of **3l** in CDCl₃

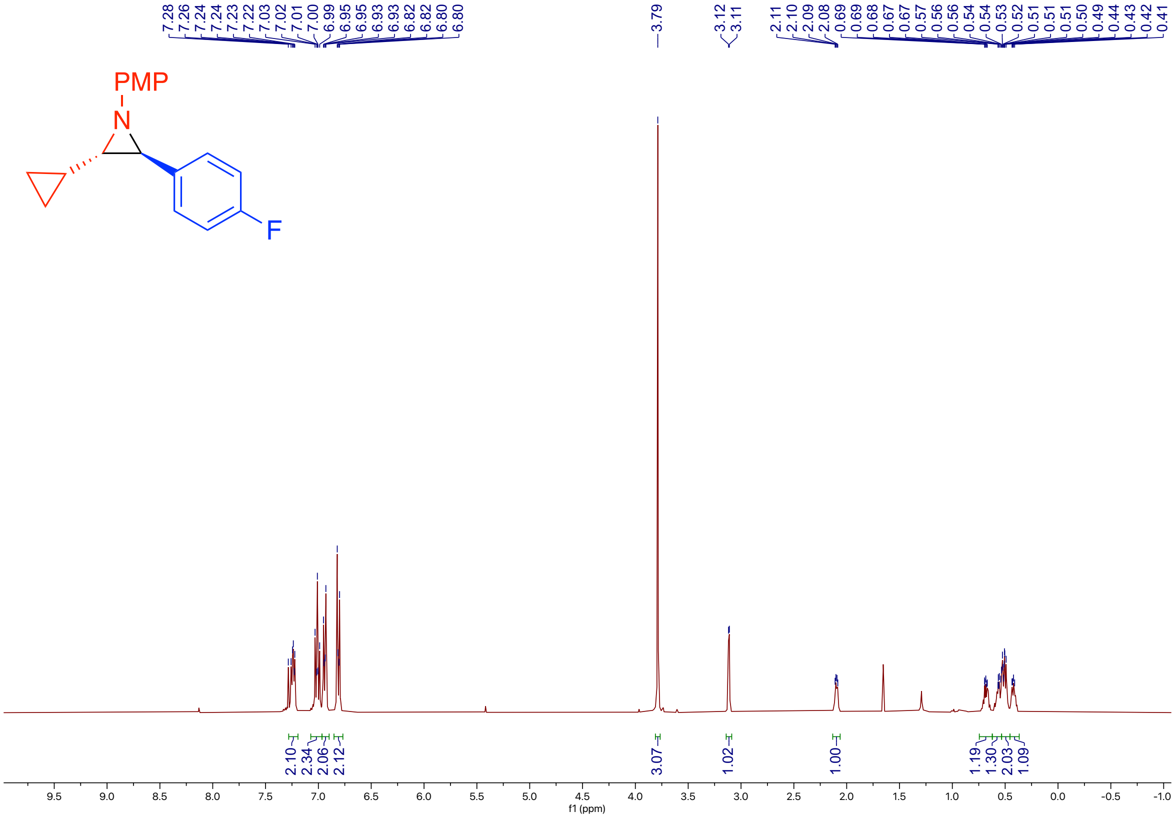

101 MHz ¹³C{¹H} NMR spectrum of **3l** in CDCl₃

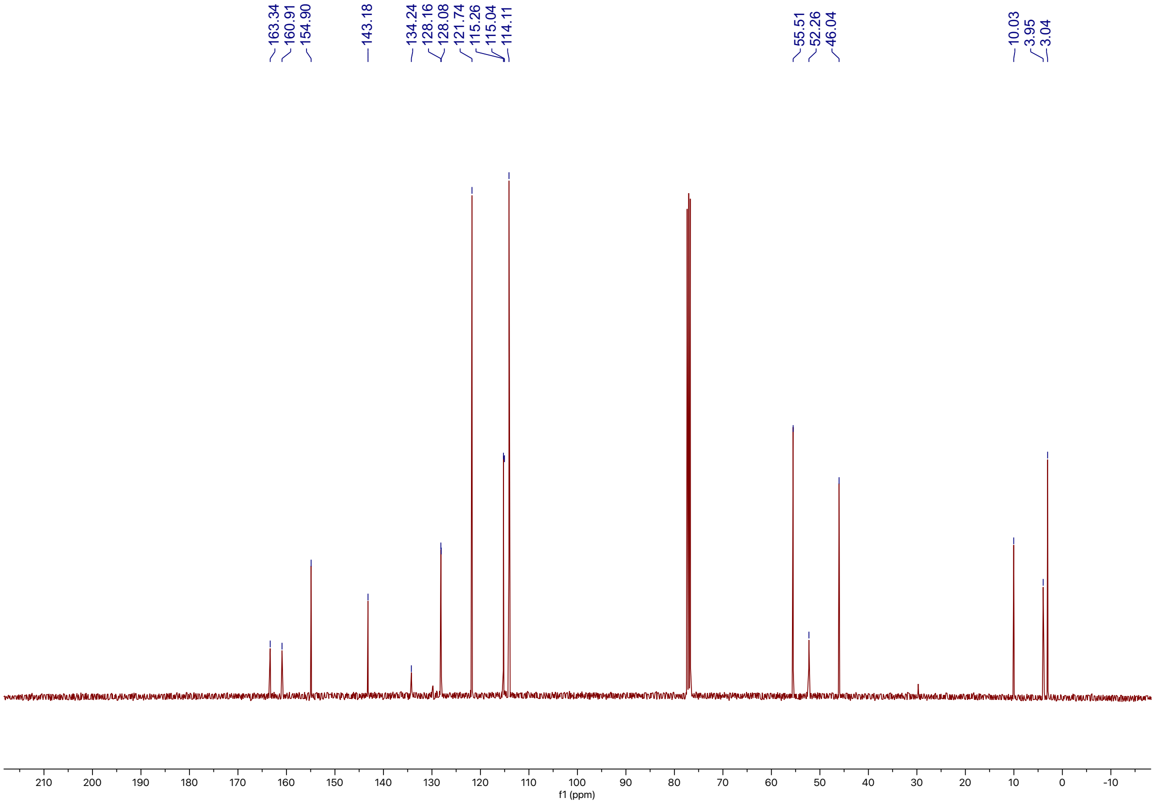

376 MHz $^{19}F\{^{1}H\}$ NMR spectrum of **3l** in $CDCl_3$

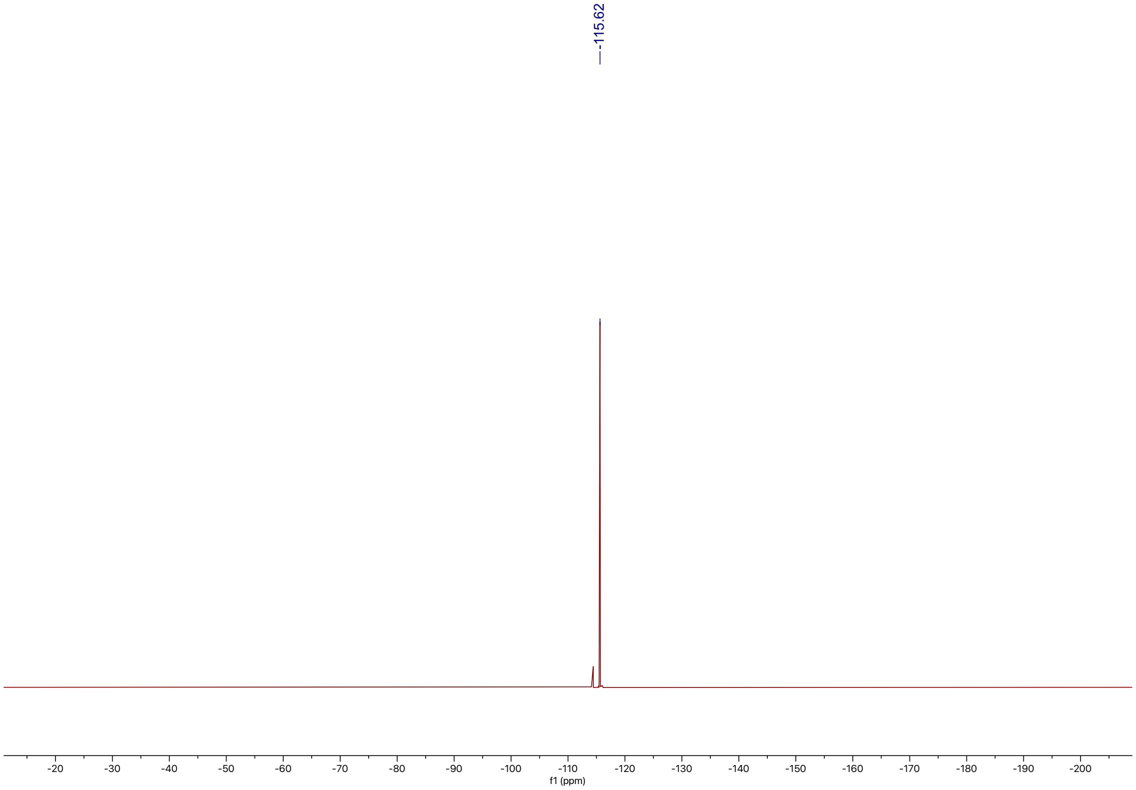

400 MHz ^{1}H NMR spectra of **3m** in $CDCl_3$

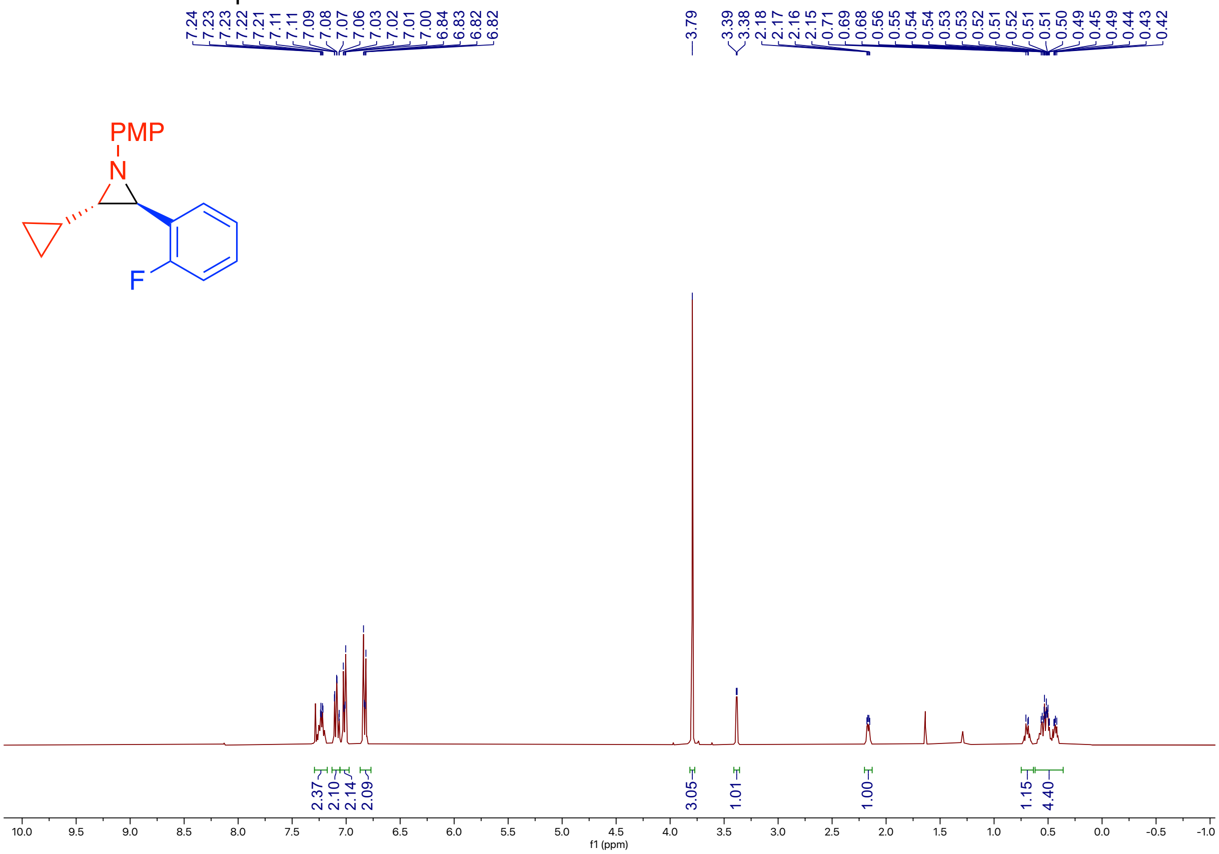

101 MHz $^{13}C\{^{1}H\}$ NMR spectrum of **3m** in $CDCl_3$

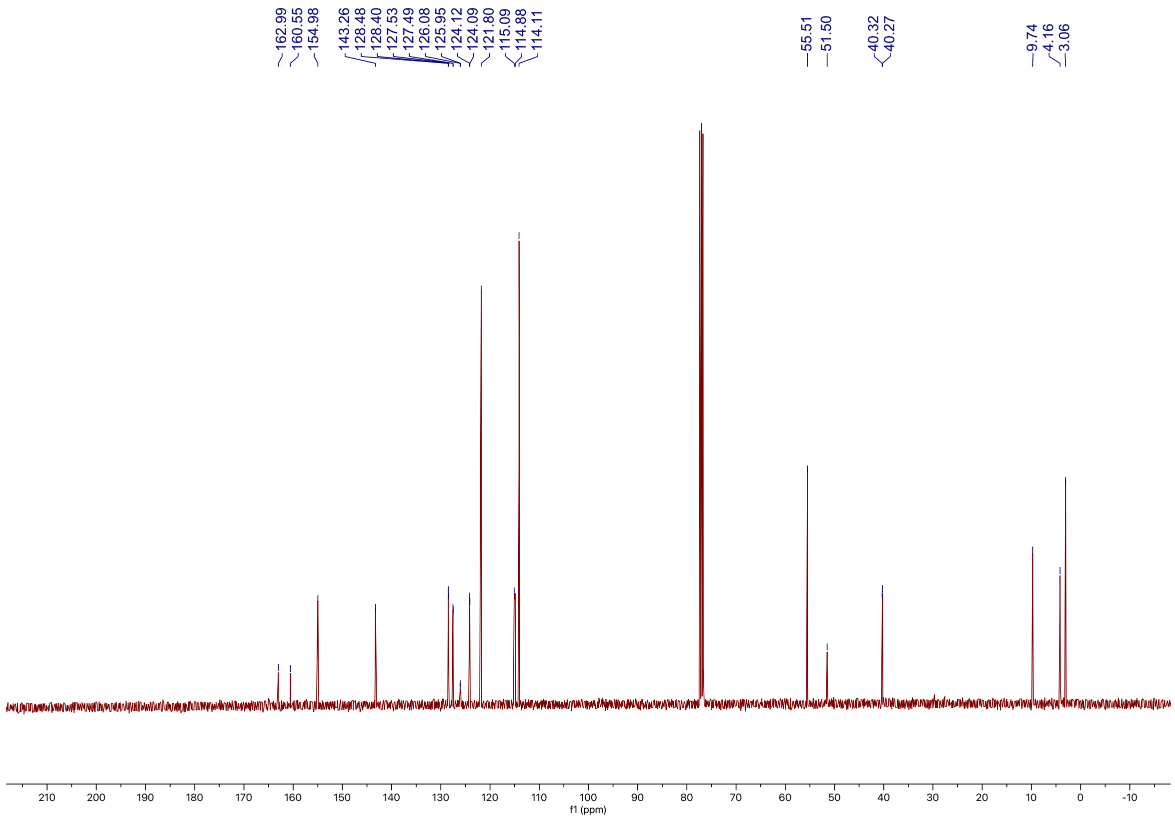

376 MHz $^{19}F\{^{1}H\}$ NMR spectrum of **3m** in $CDCl_3$

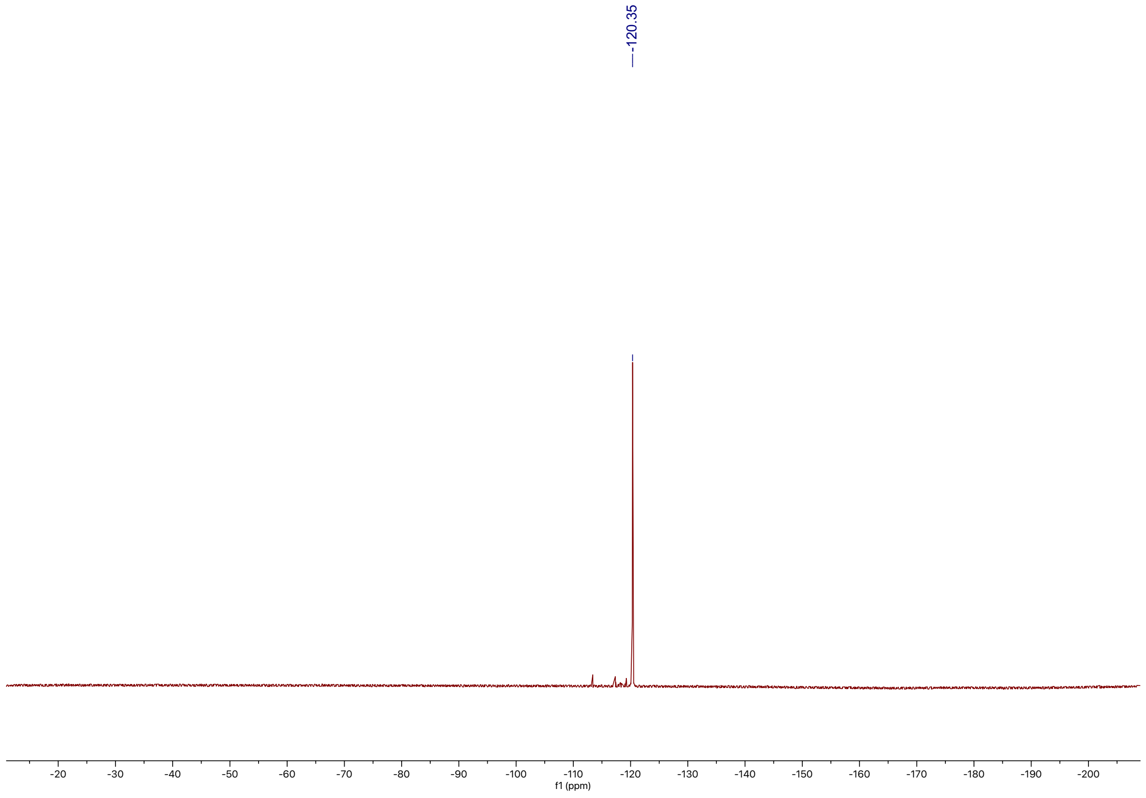

400 MHz ^{1}H NMR spectra of **3n** in $CDCl_3$

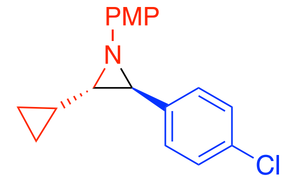

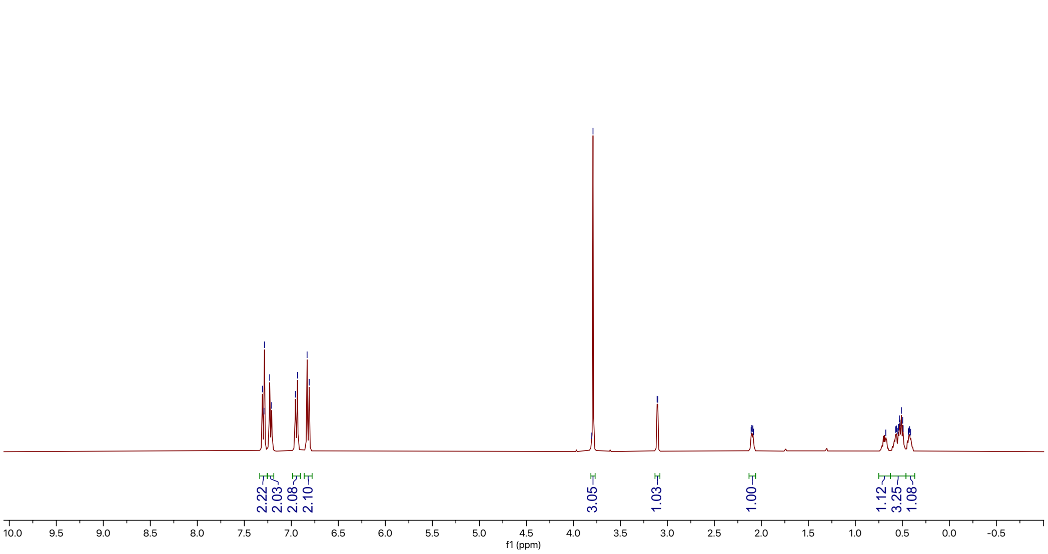

101 MHz ^{13}C{^{1}H} NMR spectrum of **3n** in $CDCl_3$

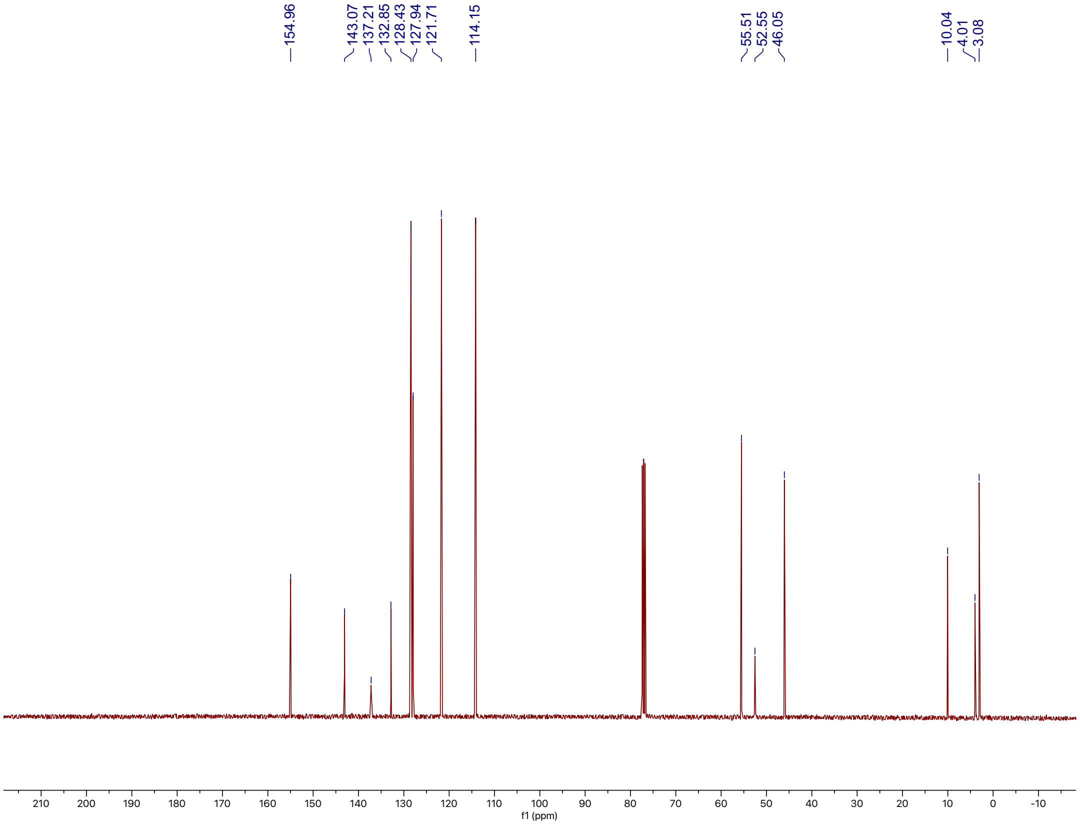

400 MHz ^{1}H NMR spectra of **3o** in $CDCl_3$

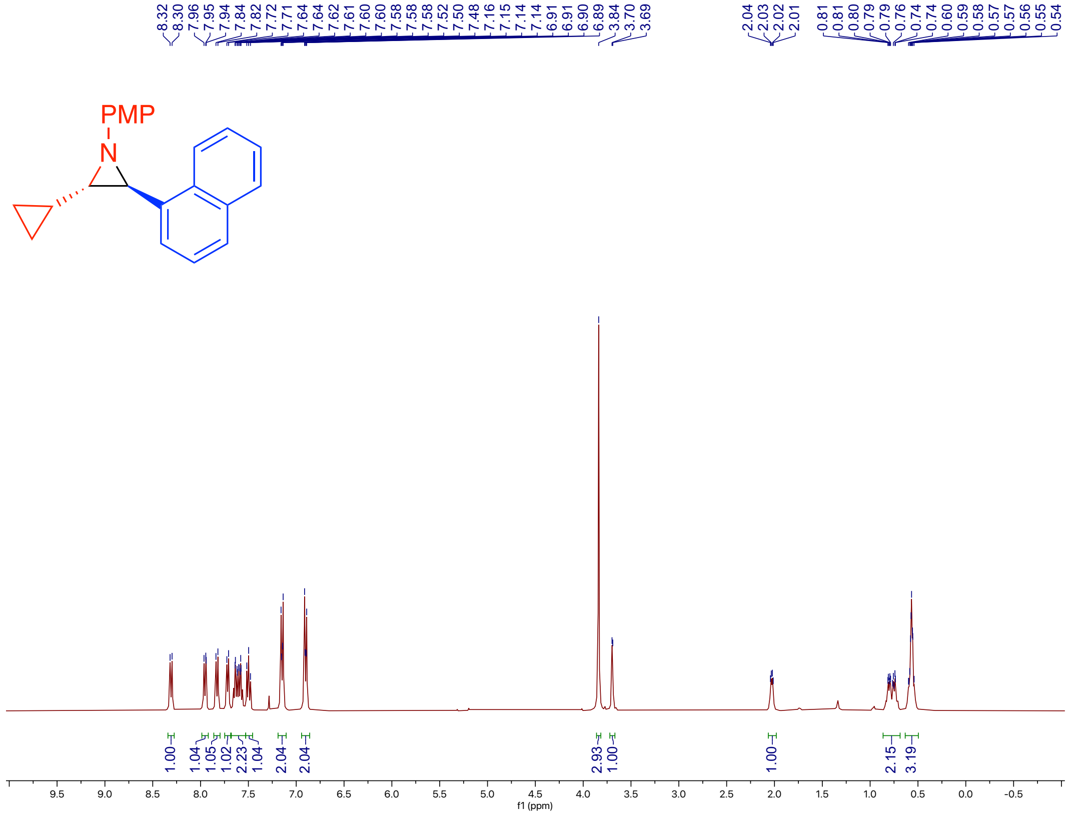

101 MHz $^{13}C\{^{1}H\}$ NMR spectrum of **3o** in $CDCl_3$

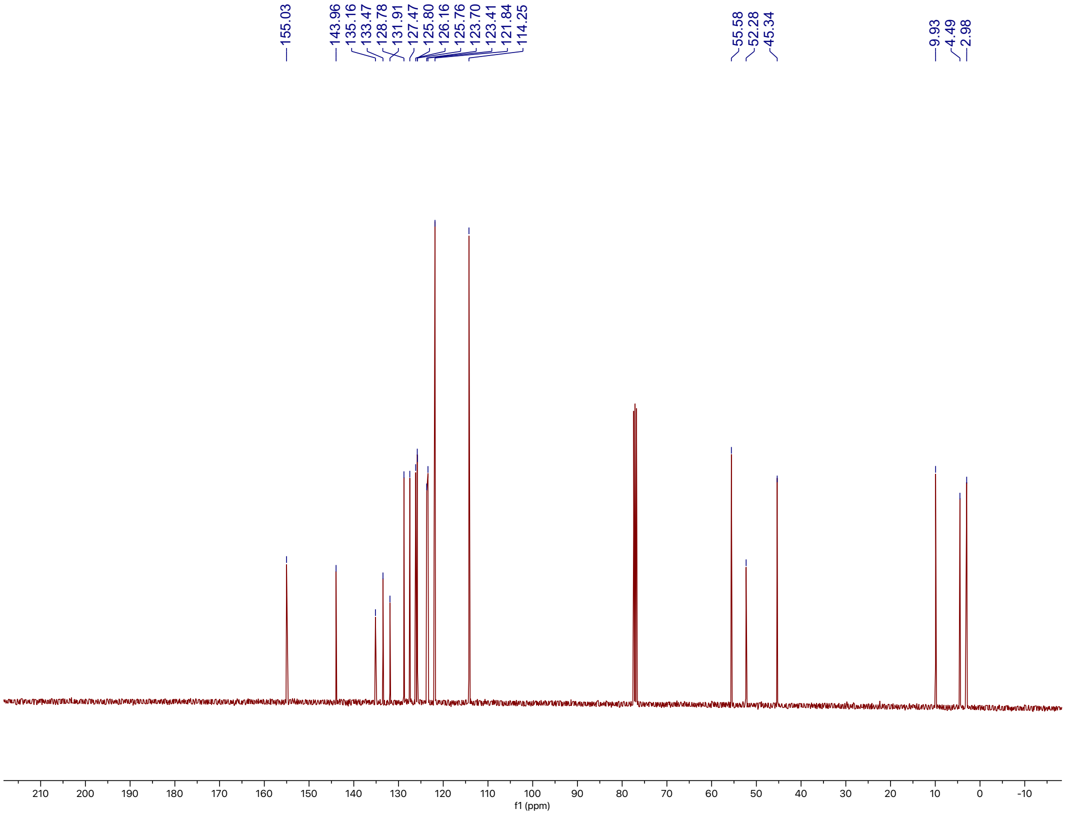

400 MHz ^{1}H NMR spectra of **3p** in CDCl$_3$

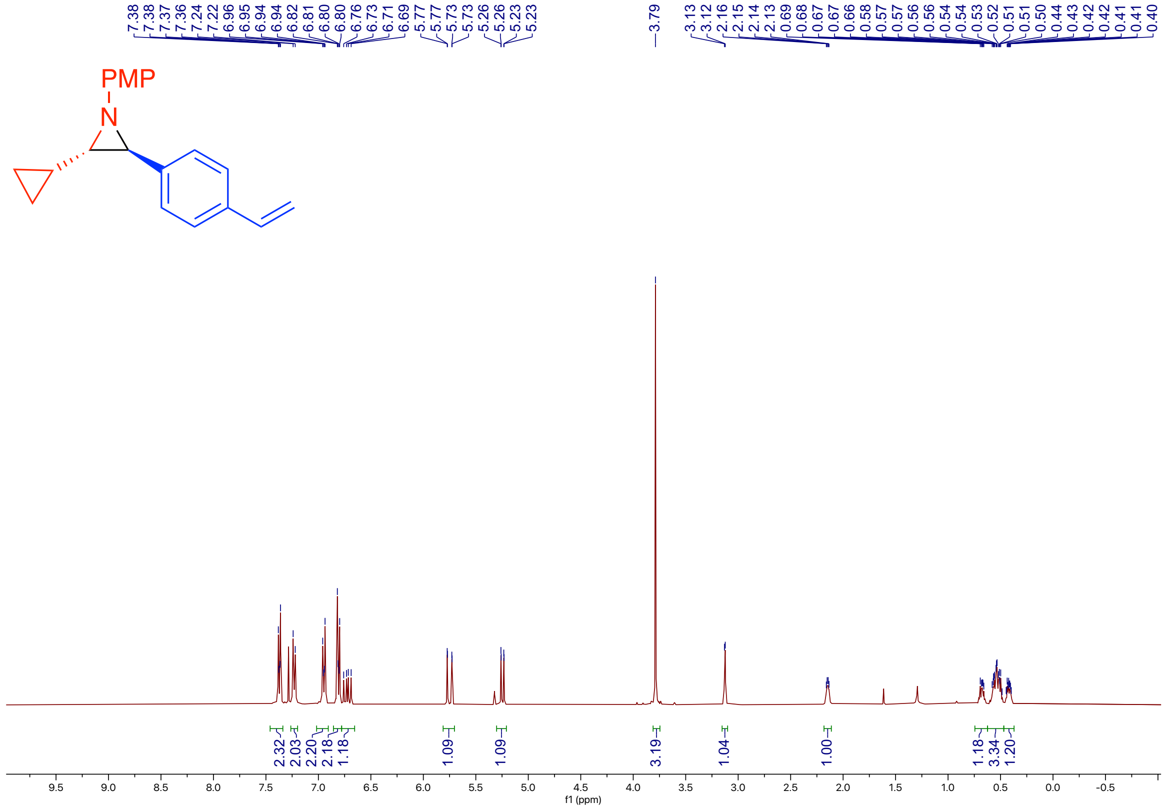

101 MHz ^{13}C{^{1}H} NMR spectrum of **3p** in CDCl$_3$

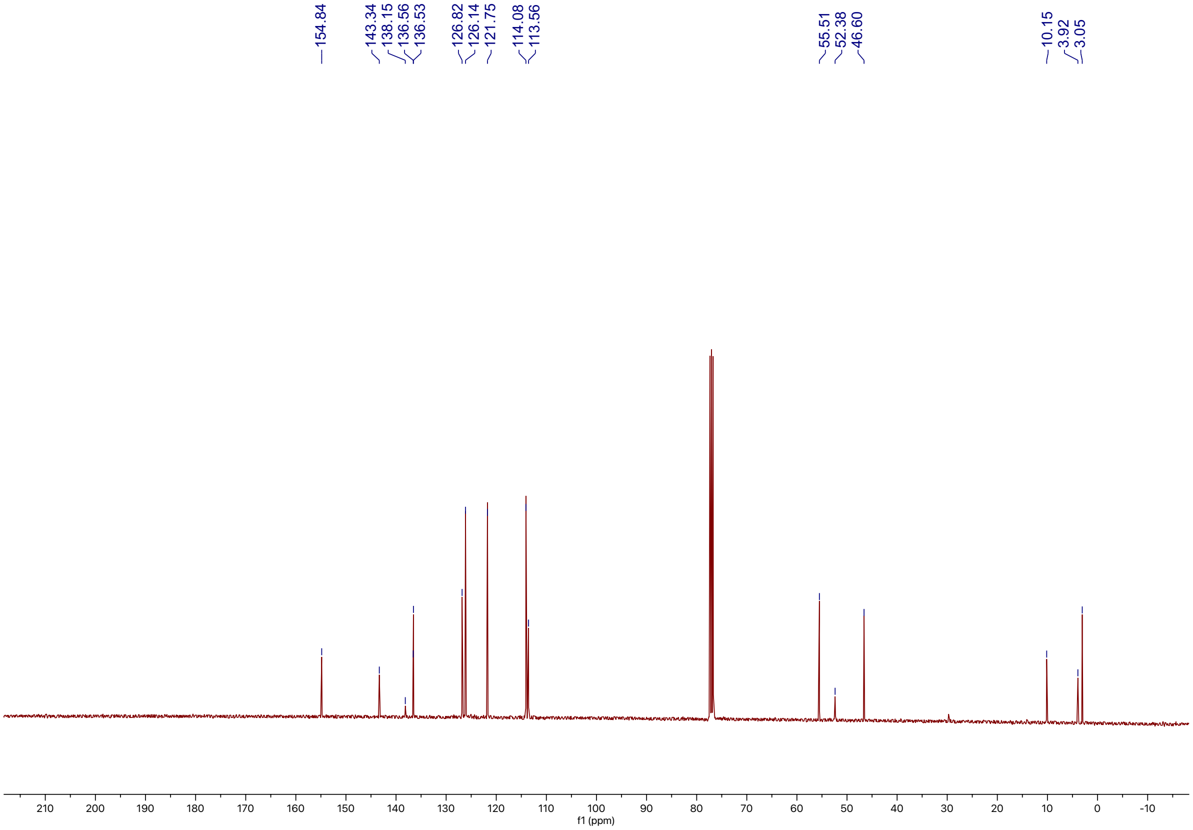

400 MHz ^{1}H NMR spectra of **3q** in $CDCl_3$

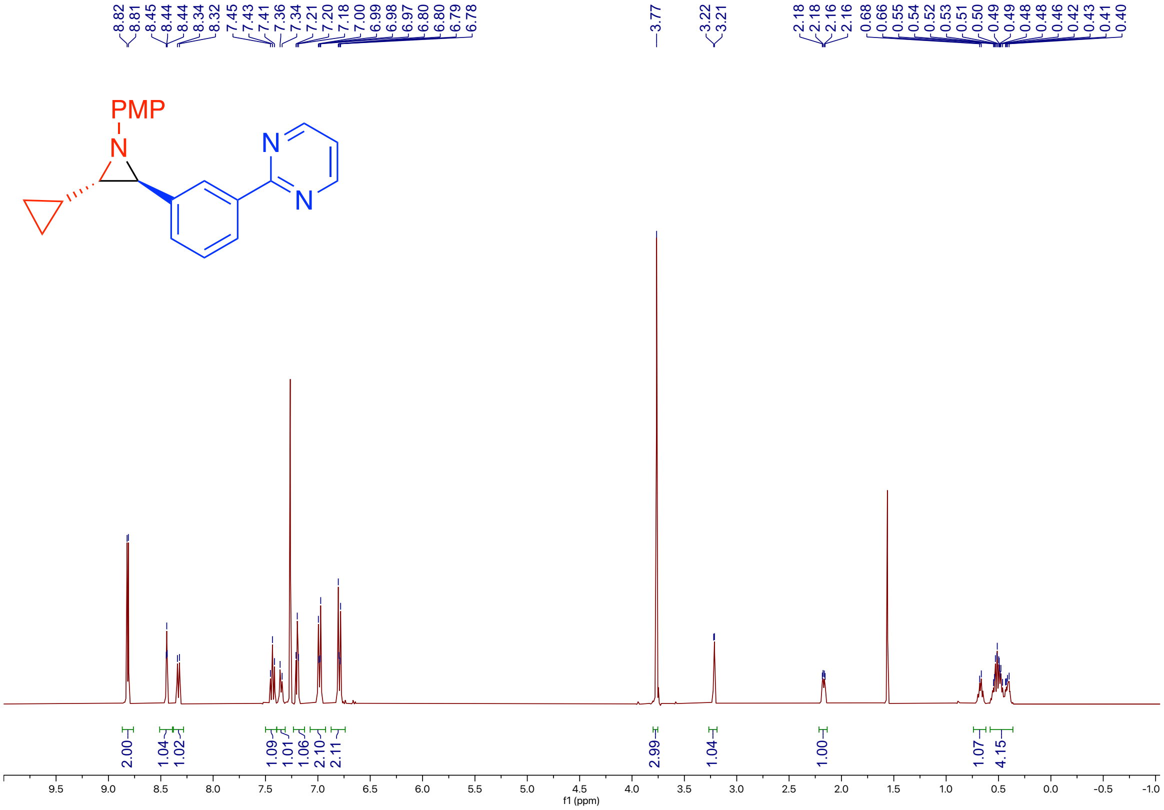

101 MHz ^{13}C{^{1}H} NMR spectrum of **3q** in $CDCl_3$

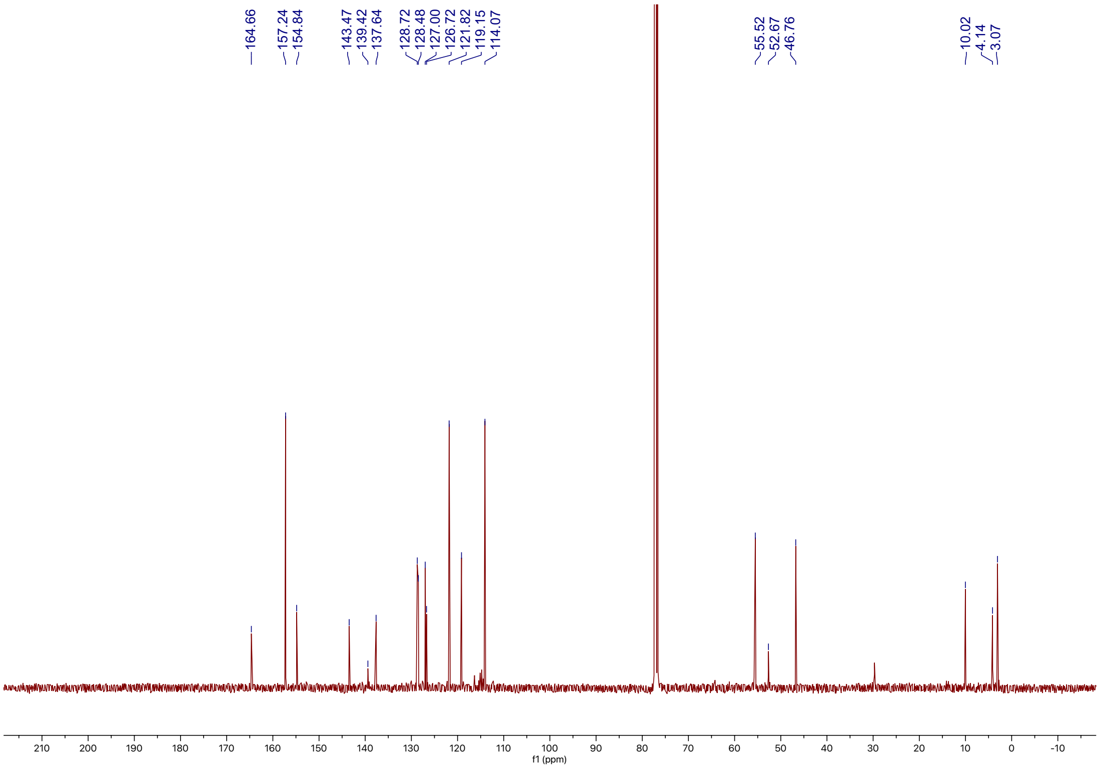

400 MHz ^{1}H NMR spectra of **3r** in $CDCl_3$

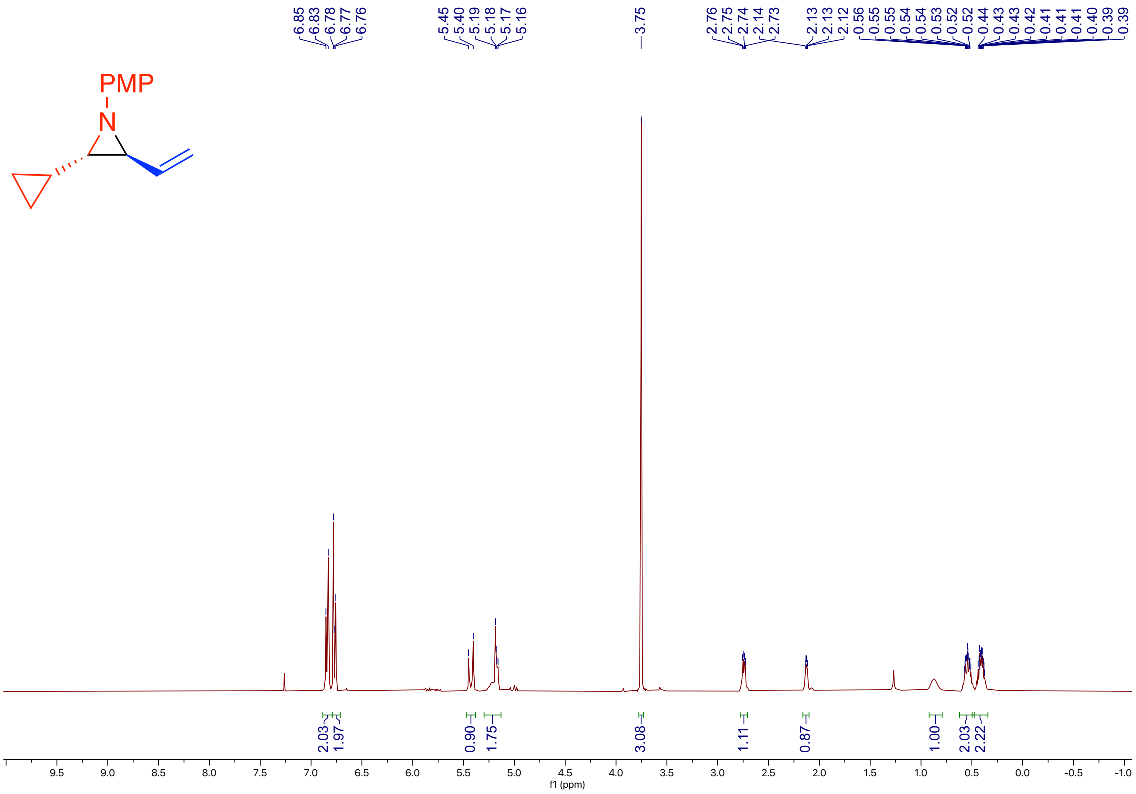

101 MHz $^{13}C\{^{1}H\}$ NMR spectrum of **3r** in $CDCl_3$

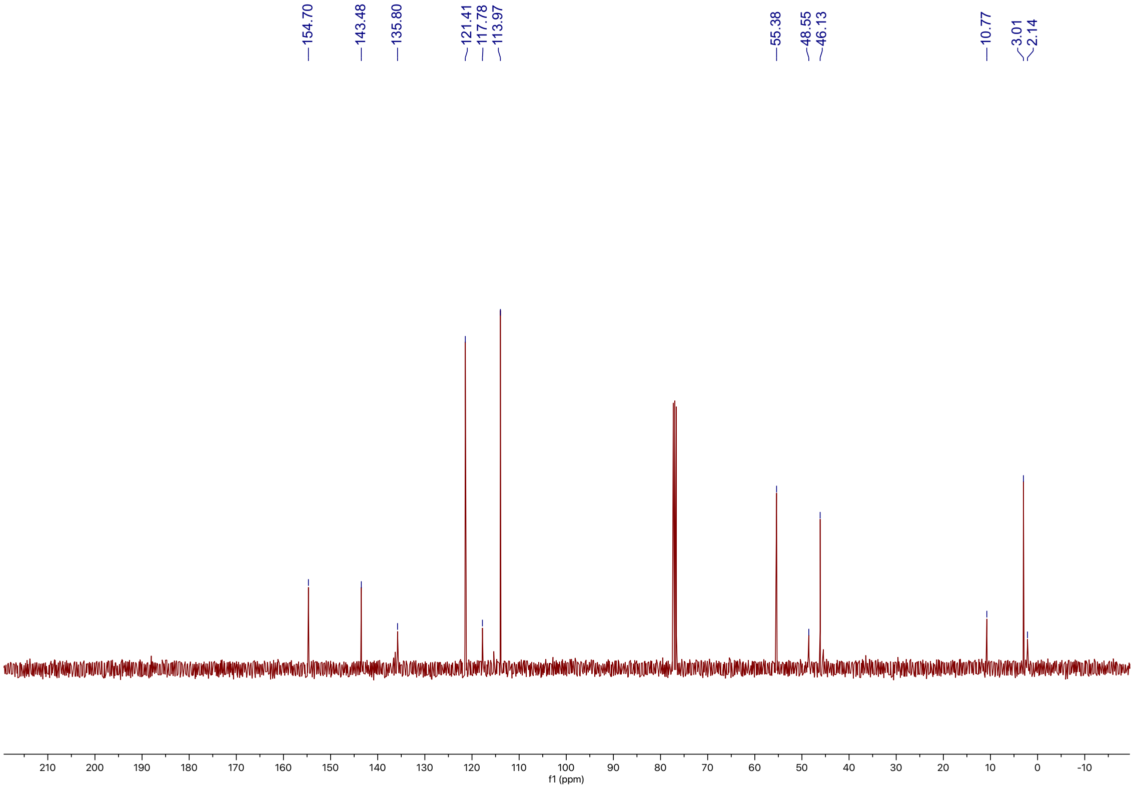

400 MHz ^{1}H NMR spectra of **3ga** in $CDCl_3$

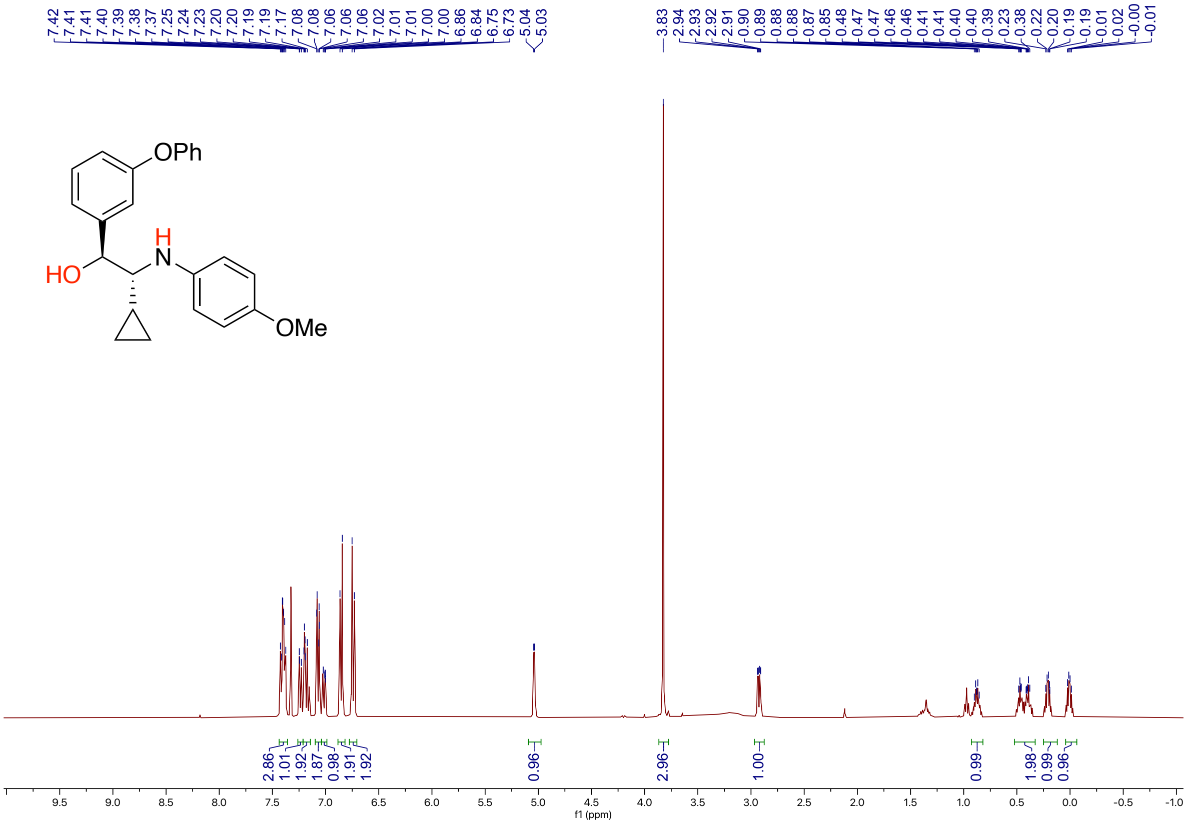

101 MHz ^{13}C{^{1}H} NMR spectrum of **3ga** in $CDCl_3$

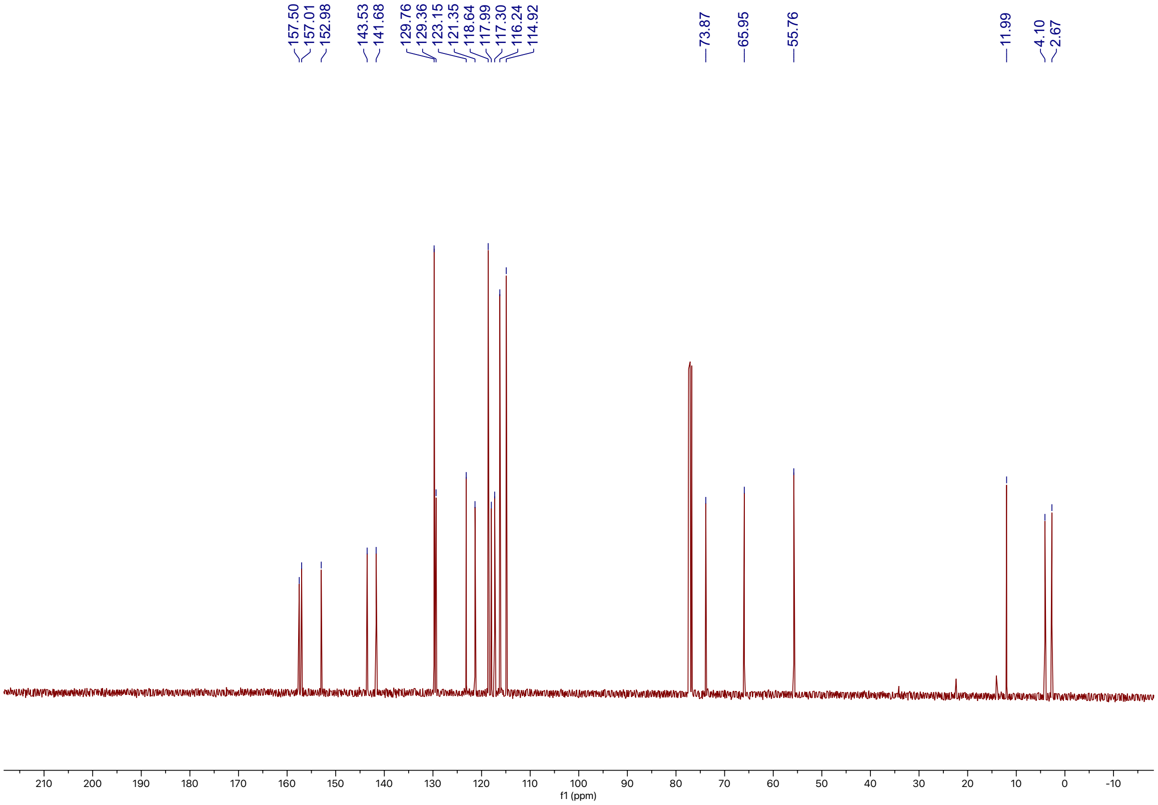

400 MHz ^{1}H NMR spectra of **3gb** in CDCl$_3$

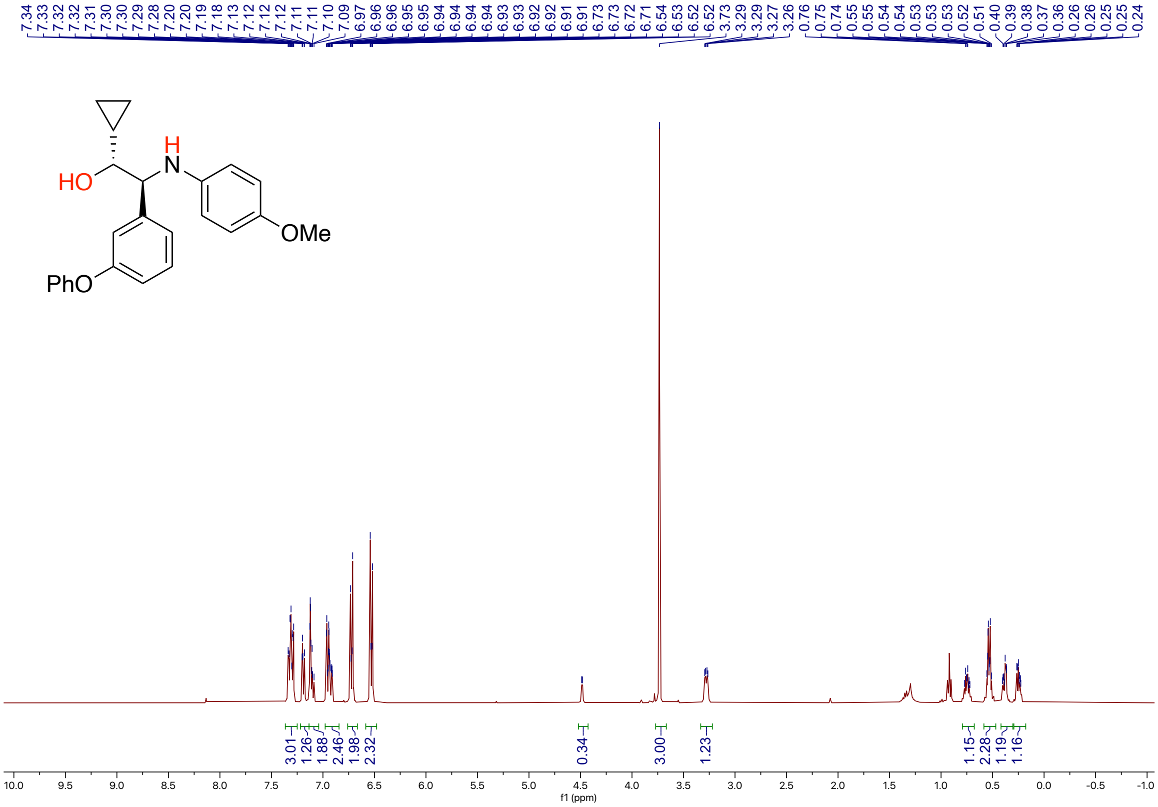

101 MHz ^{13}C{^{1}H} NMR spectrum of **3gb** in CDCl$_3$

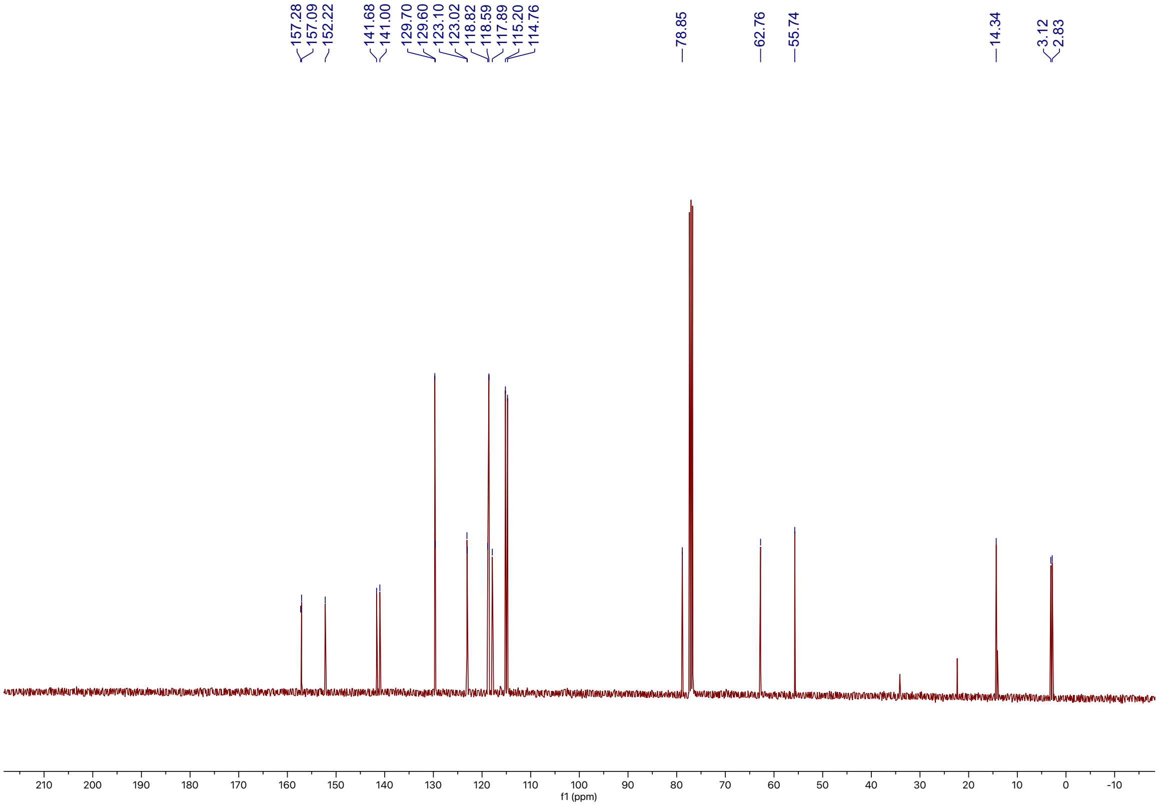

400 MHz ^{1}H NMR spectra of **7** in CDCl$_3$

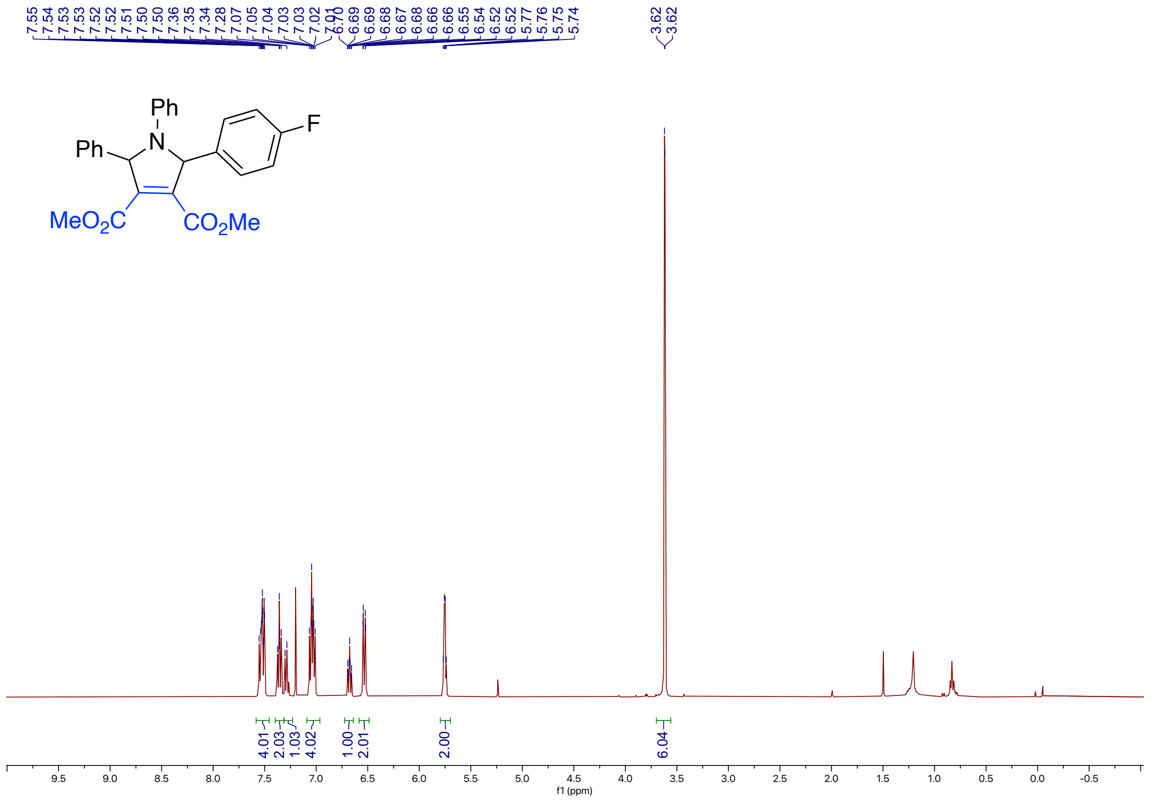

101 MHz $^{13}C\{^1H\}$ NMR spectrum of **7** in $CDCl_3$

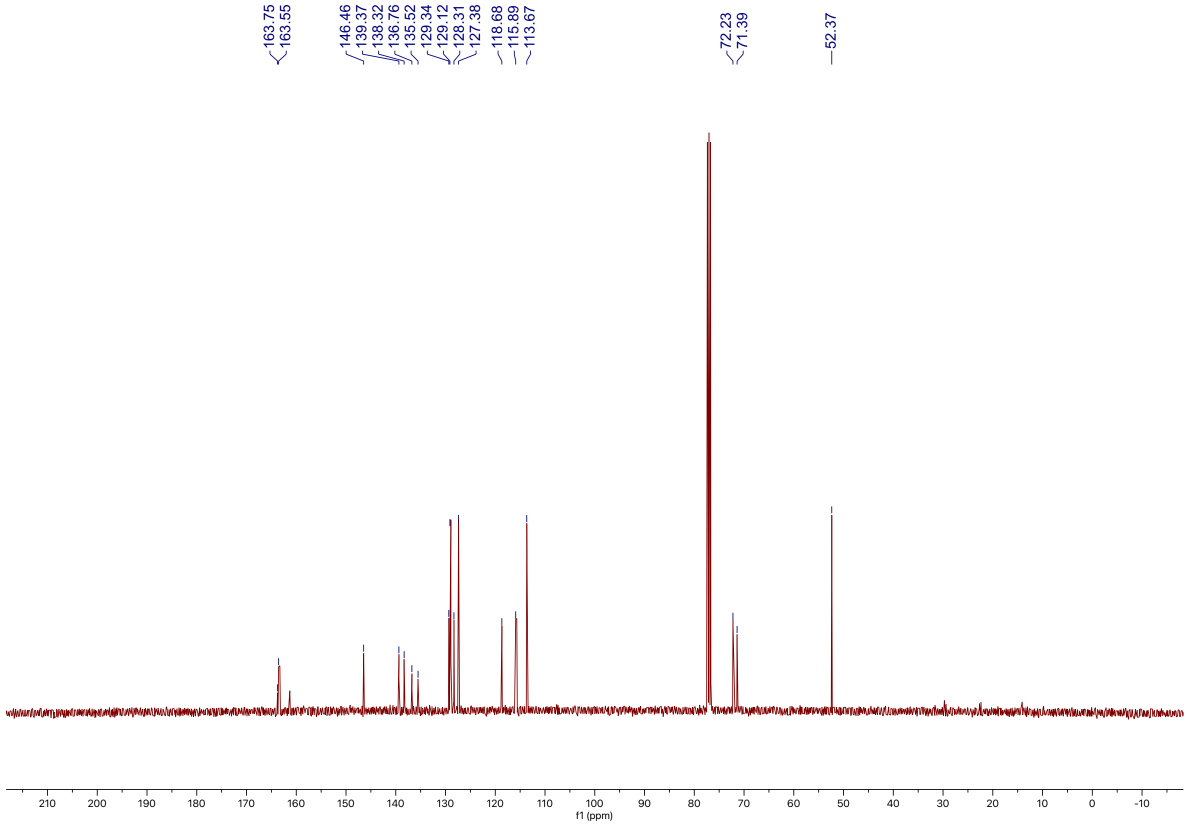

376 MHz $^{19}F\{^1H\}$ NMR spectrum of **7** in $CDCl_3$

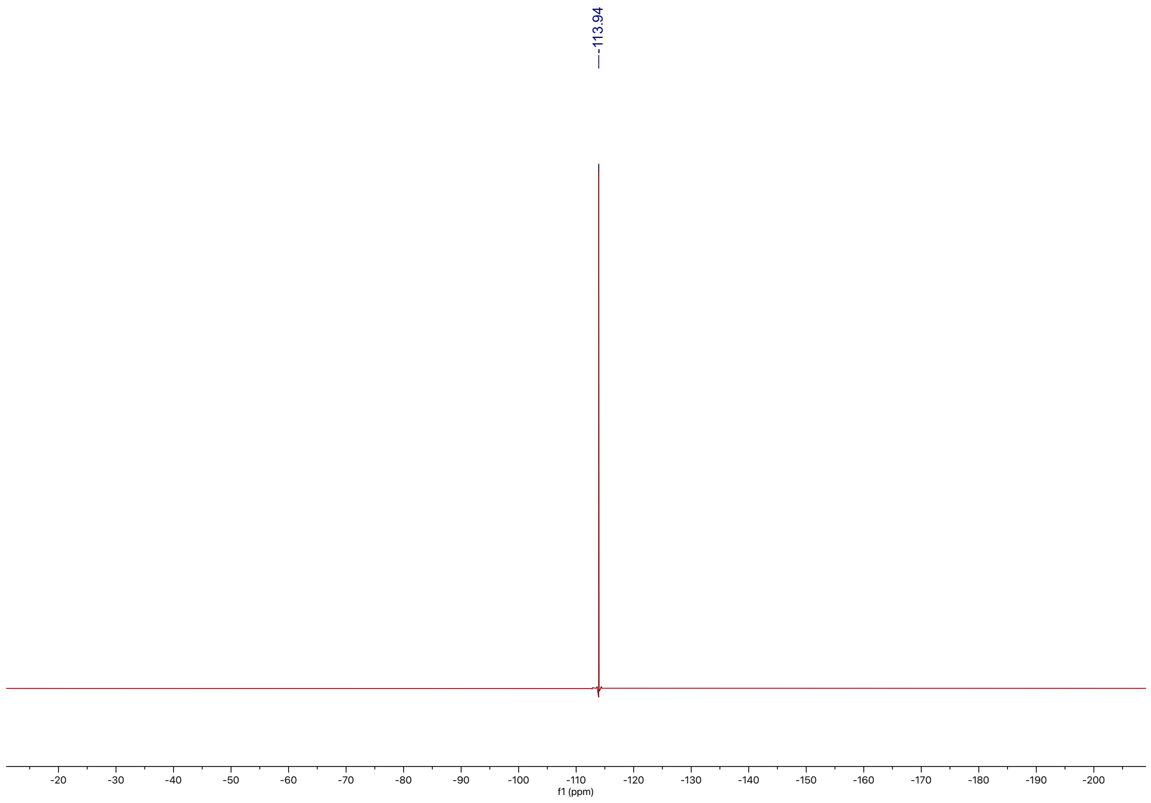
-113.94
-20
-30
-40
-50
-60
-70
-80
-90
-100
-110
-120
-130
-140
-150
-160
-170
-180
-190
-200
f1 (ppm)
